# Survival Response to Increased Ceramide Involves Metabolic Adaptation through Novel Regulators of Glycolysis and Lipolysis

**DOI:** 10.1371/journal.pgen.1003556

**Published:** 2013-06-20

**Authors:** Niraj K. Nirala, Motiur Rahman, Stanley M. Walls, Alka Singh, Lihua Julie Zhu, Takeshi Bamba, Eiichiro Fukusaki, Sargur M. Srideshikan, Greg L. Harris, Y. Tony Ip, Rolf Bodmer, Usha R. Acharya

**Affiliations:** 1 Program in Gene Function and Expression, University of Massachusetts Medical School, Worcester, Massachusetts, United States of America; 2 Program in Molecular Medicine, University of Massachusetts Medical School, Worcester, Massachusetts, United States of America; 3 Cell and Molecular Biology Program, San Diego State University, San Diego, California, United States of America; 4 Program in Bioinformatics and Integrative Biology, University of Massachusetts Medical School, Worcester, Massachusetts, United States of America; 5 Department of Biotechnology, Graduate School of Engineering, Osaka University, Osaka, Japan; 6 Laboratory of Cell and Developmental Signaling, National Cancer Institute, Frederick, Maryland, United States of America; 7 Development and Aging Program, Sanford-Burnham Medical Research Institute, La Jolla, California, United States of America; University of California San Francisco, United States of America

## Abstract

The sphingolipid ceramide elicits several stress responses, however, organisms survive despite increased ceramide but how they do so is poorly understood. We demonstrate here that the AKT/FOXO pathway regulates survival in increased ceramide environment by metabolic adaptation involving changes in glycolysis and lipolysis through novel downstream targets. We show that ceramide kinase mutants accumulate ceramide and this leads to reduction in energy levels due to compromised oxidative phosphorylation. Mutants show increased activation of Akt and a consequent decrease in FOXO levels. These changes lead to enhanced glycolysis by upregulating the activity of phosphoglyceromutase, enolase, pyruvate kinase, and lactate dehydrogenase to provide energy. A second major consequence of AKT/FOXO reprogramming in the mutants is the increased mobilization of lipid from the gut through novel lipase targets, CG8093 and CG6277 for energy contribution. Ubiquitous reduction of these targets by knockdown experiments results in semi or total lethality of the mutants, demonstrating the importance of activating them. The efficiency of these adaptive mechanisms decreases with age and leads to reduction in adult life span of the mutants. In particular, mutants develop cardiac dysfunction with age, likely reflecting the high energy requirement of a well-functioning heart. The lipases also regulate physiological triacylglycerol homeostasis and are important for energy metabolism since midgut specific reduction of them in wild type flies results in increased sensitivity to starvation and accumulation of triglycerides leading to cardiac defects. The central findings of increased AKT activation, decreased FOXO level and activation of phosphoglyceromutase and pyruvate kinase are also observed in mice heterozygous for ceramide transfer protein suggesting a conserved role of this pathway in mammals. These data reveal novel glycolytic and non-autonomous lipolytic pathways in response to increased ceramide for sustenance of high energy demanding organ functions like the heart.

## Author Summary

Ceramide belongs to an important class of lipids called sphingolipids. An increase in ceramide levels elicits several stress responses. We have used *Drosophila* mutated in ceramide kinase, an enzyme involved in metabolizing ceramide to understand how an organism adapts to stress imposed by ceramide. We find that in the mutants, increased ceramide decreases energy production via mitochondrial oxidative phosphorylation. The mutants compensate for decreased energy by upregulating glycolysis, a central pathway of intermediary metabolism. Another important mechanism that is activated in these ceramide kinase mutants is lipolysis, which is involved in breakdown of triglycerides for energy contribution. The mutants show increased hydrolysis of triglycerides in the gut, probably to provide sufficient energy for optimal performance of the heart, a high energy demanding organ. The upregulation of these pathways in the mutant result from increased activation of AKT and decreased FOXO. The modulation of glycolysis and lipolysis by AKT/FOXO is mediated by novel targets identified in this study. Failure of these adaptive mechanisms in aging flies lead to triglyceride accumulation, cardiac abnormalities, and ultimately a decrease in the lifespan of the mutant animals. The mechanisms identified here could have clinical implications in diseases associated with ceramide such as diabetes and obesity.

## Introduction

Energy balance is regulated by complex homeostatic control of lipid and glucose metabolism involving signaling pathways in multiple tissues and organs. Aberrant glucose and lipid metabolism arising from defects in these pathways accompanies a number of diseases including cardiovascular disorders, type II diabetes and metabolic syndrome associated with obesity and insulin resistance. Understanding regulatory mechanisms governing glucose and lipid metabolism is fundamental for identifying potential therapeutic targets for treatment of these metabolic diseases. Altered lipid metabolism is one of the important contributors to obesity and insulin resistance. Dyslipidemia in obesity is characterized by elevated free fatty acids, triglycerides (TAG), VLDL and decreased HDL cholesterol. Increased levels of free fatty acids and lipids such as diacylglycerol and ceramide mediate insulin resistance by redistribution of fat metabolites to tissues not suited for lipid storage [1], [2].

The sphingolipid, ceramide, is an integral component of cell membranes and also a bioactive lipid [3]. Ceramide elicits many cell-stress responses including apoptosis, senescence, inflammation, mitochondrial dysfunction and recent studies show that it can affect cellular metabolism. It can impair insulin stimulated glucose uptake, it serves as an intermediate linking glucocorticoids and saturated fatty acids to insulin resistance and has been implicated in the pathogenesis of lipotoxic cardiomyopathy [4]–[6]. Strategies that pharmacologically or genetically decrease ceramide have beneficial effects in reversing insulin resistance, preventing apoptosis of pancreatic β-cells and cardiomyocytes [4], [7].

Despite the involvement of ceramide in many stress responses, organisms develop and survive with increased ceramide levels in certain circumstances. For example, *Drosophila* mutants of ceramide kinase and brainwashing (mammalian homolog of alkaline ceramidase) are viable while mutants of ceramidase are lethal despite similar increase in ceramide levels [8]–[11]. Mice and *Arabidopsis* deficient in ceramide kinase and mice deficient in sphingomyelin synthase 1 and 2 are viable despite accumulation of ceramide [12]–[16]. A likely explanation is that these organisms implement adaptive responses that allow them to survive and maintain equilibrium in the face of stress due to increased ceramide. These adaptive mechanisms are of critical importance in understanding metabolic homeostasis, but have received limited attention especially in multi-cellular organisms. In this study, we address this issue using *Drosophila* loss of function mutation in ceramide kinase (CERK). Our earlier studies established CERK as an important regulator of phospholipase C (PLC) signaling and photoreceptor homeostasis [10]. A mutation in dCERK led to proteolysis of PLC, consequent loss of PLC activity and failure in light signal transduction. These defects were due to increased ceramide levels and not ceramide 1-phosphate (C1P) since levels of C1P did not change significantly in photoreceptors of *dcerk^1^* mutants.

In recent years, *Drosophila* has emerged as a useful organism to study metabolism [17]. The fly has organ systems such as the fat body (adipose tissue), oenocytes (hepatocyte-like cells), gut (gastrointestinal tract) and malphigian tubules (kidneys) that parallel those in mammals. Several studies highlight the conserved mechanisms of carbohydrate, lipid and energy homeostasis in flies and genes discovered in *Drosophila* have provided information relevant to human physiology including that of the heart [18]–[27].

We set out to globally discover what genes and metabolites change in ceramide kinase mutant flies (*dcerk^1^*) by gene expression and metabolic profiling. Integrating information from these analyses with genetic and biochemical experiments, we show that *dcerk^1^* mutants survive due to increased activation of AKT and decreased FOXO level. In the *dcerk^1^* mutants, increase in ceramide leads to decreased ATP level due to compromised mitochondrial oxidative phosphorylation. Metabolic reprogramming to ceramide involves AKT induced glycolytic utilization of glucose through activation of phosphoglycerate mutase and also utilization of triglycerides by activation of two intestine specific lipases, CG8093 and CG6277 in *dcerk^1^*. These are novel downstream targets of AKT/FOXO in glycolysis and lipolysis. With age, these compensatory mechanisms fail leading to abnormal cardiac function and decreased life span of the mutants. Gut specific reduction in these lipases in both wild type and mutant flies results in TAG accumulation, increased sensitivity to starvation and cardiac dysfunction. Using mice heterozygous for ceramide transfer protein (CERT) as a second model of ceramide increase, we substantiate AKT activation, decrease in FOXO level and activation of glycolytic genes, phosphoglycerate mutase and pyruvate kinase in a mammalian system [28]. We have thus discovered a novel connection in the PI3K-AKT-FOXO pathway for survival and metabolic adaptation to a ceramide environment that participates in energy response involving phosphoglycerate mutase and gut specific lipases, CG8093 and CG6277 as physiological targets.

## Results

### *dcerk^1^* mutants exhibit metabolic reprogramming

**Figure 1:**
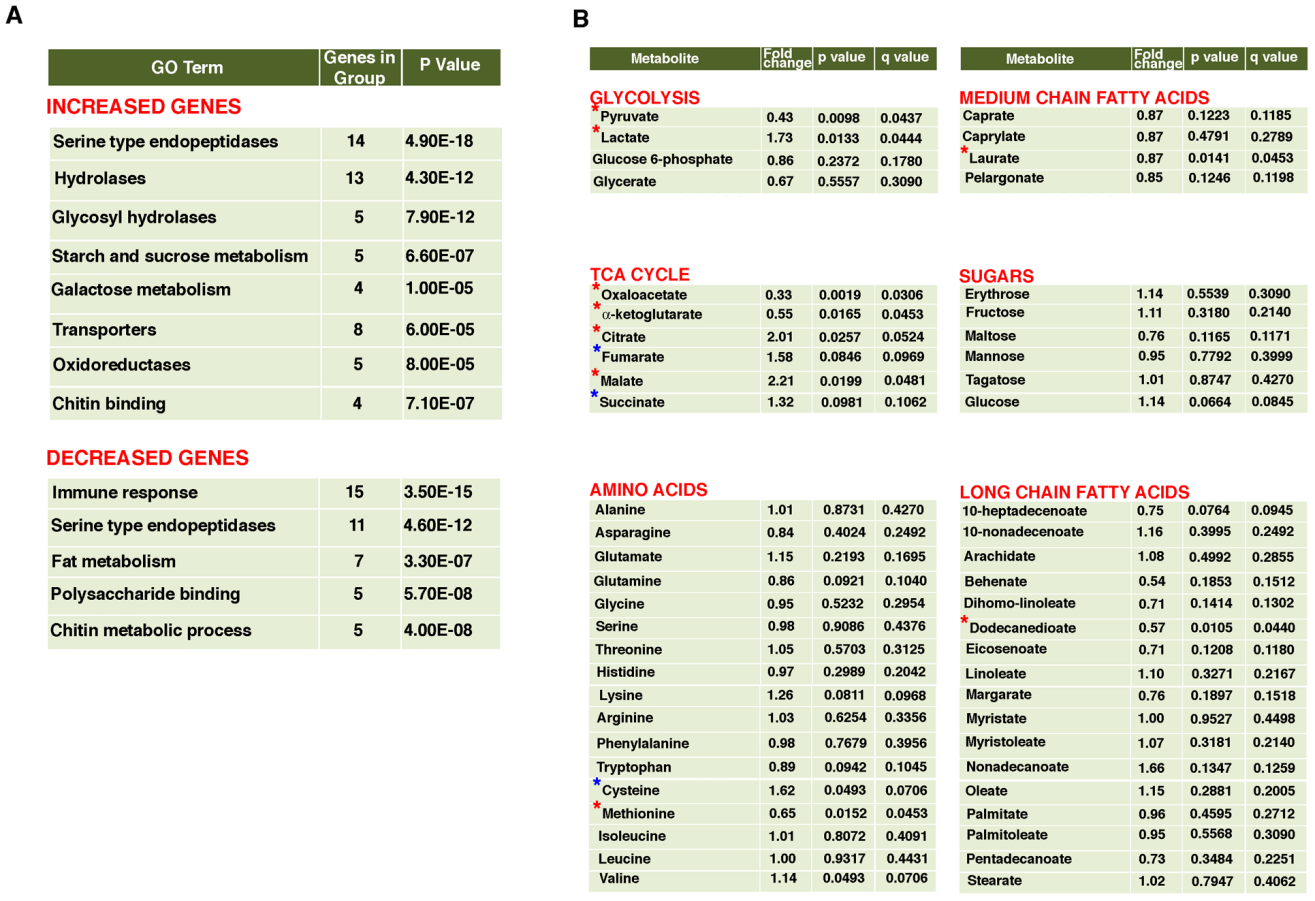
Metabolic reprogramming in *dcerk^1^* mutants. A. Functional classification of genes whose expression is altered in *dcerk^1^* mutants compared to control *w^1118^* **flies.** RNA extracted from control and mutant flies was hybridized to Affymetrix Drosophila 2.0 microarrays and experiments were carried out in triplicate. Gene expression analysis revealed 152 genes were increased (2-fold or more) and 158 genes were decreased (0.5 fold or less) in *dcerk^1^* compared to *w^1118^*. DAVID gene functional annotation tool and functional classification tool were used to annotate and classify the increased and decreased genes into functionally related gene groups. The top GO terms obtained for increased and decreased genes are listed along with the number of genes altered in each category and the statistical significance based on modified Fisher Exact P-Value. Serine type endopeptidases and hydrolases are upregulated while immune response genes are downregulated in the mutant. B. Metabolomic profiling reveals changes in glycolysis and TCA cycle metabolites in *dcerk^1^* compared to *w^1118^***.** Control and mutant fly extracts were subjected in triplicate to GC/MS, LC/MS and LC/MS/MS platforms to compare relative levels of several metabolites. The fold change, *dcerk^1^*/*w^1118^* is shown for each metabolite. A p value of <0.05 and a q value of <0.1 (false discovery rate) is considered highly statistically significant (marked by a red *). Among glycolysis intermediates, pyruvate and lactate meet these stringent criteria; pyruvate level is decreased while lactate level is increased in the mutant. Among TCA cycle metabolites, oxaloacetate and α-ketoglutarate are decreased while citrate and malate are increased. Fumarate and succinate are also increased and meet the less stringent statistical criteria of approaching significance (p 0.05 −0.1 and q 0.1 or less, marked by a blue *). Most amino acids (other than cysteine and methionine) and sugars do not show significant differences. Among fatty acids, Dodecanedioate, a long chain fatty acid is decreased significantly in the mutant. This could be due to its degradation to acetyl CoA and succinate, the latter being important for gluconeogenesis during starvation.

Ceramide kinase mutants show an increase in steady state concentration of ceramides without significant decrease in the levels of ceramide 1-phosphate in whole fly extracts (Figure S1). The increase in C24:1 ceramide is most dramatic (about 280%) while other ceramides show between 30–50% increase in the *dcerk^1^* mutants over control flies. Thus *dcerk^1^* mutants will have to survive in an increased ceramide environment. An organism's attempt to adapt to stress or altered environment involves changes in gene expression and metabolite levels that aid in reestablishing homeostasis. Therefore, we reasoned elucidating both transcriptional and metabolite responses that change in mutants relative to control could help us understand possible adaptive mechanisms that operate in *dcerk^1^*. We carried out a transcriptome-wide analysis in *w^1118^* and *dcerk^1^* flies. 310 transcripts were changed significantly in *dcerk^1^* compared to control, with 152 genes being upregulated (> = 2.0 fold) and 158 genes being downregulated (< = 0.5 fold, Table S1). To identify pathways and predicted gene functions corresponding to the altered transcripts, we employed the DAVID gene ontology (GO) annotation and functional classification tools [29]. The top GO class for increased genes included serine type endopeptidases and hydrolases (Figure 1A). Most of the serine type endopeptidases are mainly expressed in the larval/adult mid gut (based on FlyAtlas expression patterns) suggesting they are likely involved in the mobilization of dietary protein. Among the increased hydrolases were glycosyl hydrolases and those involved in metabolism of starch, sucrose and galactose and these are also expressed mainly in the adult gut. Thus, there is a significant upregulation of midgut genes involved in breakdown of dietary proteins and sugars in *dcerk^1^*. Other classes of upregulated genes in *dcerk^1^* were transporters, oxidoreductases and genes that constitute the peritrophic envelope, a lining composed of chitin and glycoproteins, separating the food from the midgut epithelium. The top GO category for downregulated genes were antimicrobial peptides (AMPs) involved in the innate immune response. Important metabolic genes that change in *dcerk^1^* flies are phosphoenolpyruvate carboxykinase (PEPCK, downregulated) involved in gluconeogenesis, fatty acid synthesis (downregulated), carnitine palmitoyltransferase (β-oxidation, upregulated) and pyruvate kinase (upregulated) involved in glycolysis.

Since gene expression changes in known targets of FOXO such as PEPCK and AMPs were observed, we analyzed if other differentially expressed genes in our microarray dataset could be potential targets of FOXO. Recent ChIP-chip analysis revealed about 700 direct FOXO targets in adult *Drosophila* [30]. Using this information coupled with ChIP peak Annopackage we identified that 40 genes from our microarray data contained FOXO binding sites and could be potential targets of FOXO (Table S2) [31].

### Metabolomic analysis of *dcerk^1^* mutants reveals changes in glycolysis and TCA cycle

In order to understand how changes in gene expression influence metabolism, we compared the metabolic profiles of *w^1118^* and *dcerk^1^* by mass spectrometry. About 175 metabolites were identified and classified into different functional pathways (Table S3). The fold changes (*dcerk^1^/w^1118^*) for various amino acids, fatty acids, sugars and intermediates in glycolysis, TCA cycle, are shown in Figure 1B. Two important metabolic pathways that were different between *w^1118^* and mutants were glycolysis and TCA cycle. *dcerk^1^* exhibited increase in lactate and decrease in pyruvate, both glycolytic intermediates. While oxaloacetate and α-ketoglutarate were decreased, other TCA cycle intermediates, citrate, malate, fumarate and succinate were increased. The altered steady state concentrations of the metabolites indicate change in the flux of these pathways. Glycolysis, TCA cycle and mitochondrial oxidative phophorylation share control of energy metabolism and they interact to match cellular ATP demand with ATP production.

**Figure 2:**
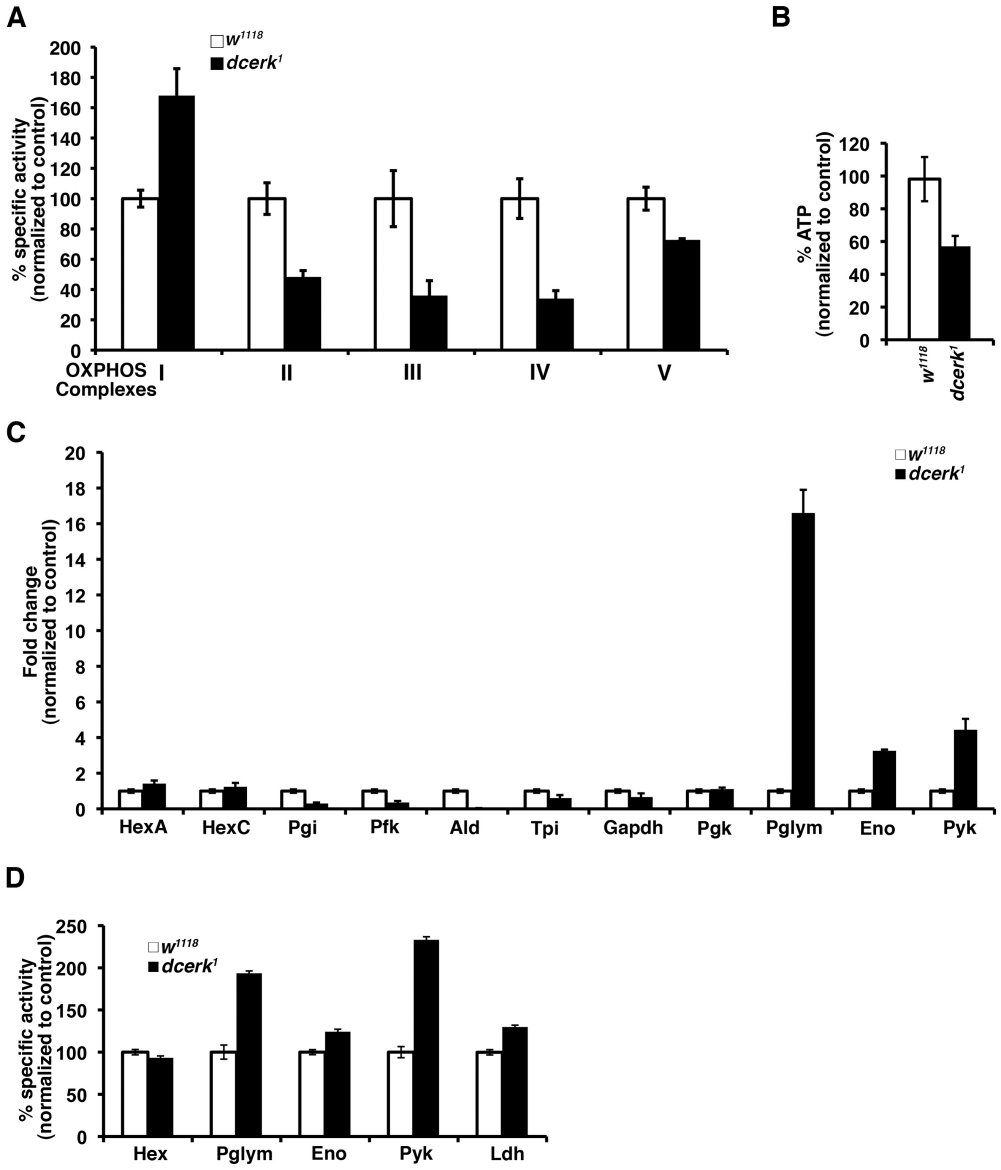
*dcerk^1^* mutants show decreased mitochondrial oxidative phosphorylation, lower ATP level, and increased glycolysis compared to control flies. (A). The activity of each of the electron transport chain complexes is measured spectrophotometrically using specific substrates and inhibitors in mitochondria isolated from control and mutant flies. Specific activity per mg of mitochondrial protein was calculated and then normalized to *w^1118^*. Mitochondria were prepared from 1000 flies and 3 replicates were carried out. Error bars represent standard deviation. (B). ATP level is measured in *w^1118^* and mutant fly mitochondria by a bioluminescence assay using luciferase catalyzed oxidation of luceferin which is ATP dependent. The amount of ATP is calculated per mg of mitochondrial protein and normalized to *w^1118^*. The relative level of ATP in the mutant mitochondria is 60% of *w^1118^*. n = 3, error bars represent standard deviation. (C). QPCR analysis of transcripts encoding glycolytic enzymes show a 16–18 fold increase in Pglym, 3–4 fold increase in Eno and 4–5 fold increase in Pyk levels in *dcerk^1^* relative to *w^1118^*. n = 3, error bars represent standard deviation. (D). Measurement of specific activities of glycolytic enzymes show increase in Pglym, Eno, Pyk and Ldh activities of *dcerk^1^* compared to *w^1118^* while that of hexokinase is not significantly different. Specific activity of each enzyme is determined per mg of protein and then normalized to *w^1118^*. n = 3, error bars represent standard deviation.

To assess mitochondrial oxidative phosphorylation, we measured the enzyme activities of the five complexes of the electron transport chain in mitochondrial-enriched fractions from control and mutant flies. As seen in Figure 2A, the activities of complexes II, III, IV and V are decreased while I is increased in the mutant compared to control. To test if compromised oxidative phosphorylation led to alteration in energy level, we measured ATP level in mitochondria isolated from *w^1118^* and mutant flies. Indeed, *dcerk^1^* showed a 40% decrease in ATP level relative to *w^1118^* (Figure 2B). Since metabolic profiling emphasized changes in glycolysis, we first examined if there are changes in gene expression of enzymes of the glycolytic pathway in addition to pyruvate kinase, which was shown to be upregulated by microarray analysis. Quantitative PCR analysis of the different glycolytic genes showed a 15-fold increase in phosphoglycerate mutase (Pglym), 3–4 fold increase in both enolase (Eno) and pyruvate kinase (Pyk) expression, enzymes that catalyze later reactions in the glycolytic pathway (Figure 2C). Interestingly, traditional regulators of glycolysis such as hexokinase and phosphofructokinase are either unchanged or decreased. To test if increase in transcript levels translated to increased activity, we measured activity of the various enzymes. Pglym, Pyk, Eno and Lactate dehydrogenase (Ldh) showed increase in enzyme activity (Figure 2D). Metabolically these changes in transcript and enzyme activity result in a 1.7 fold increase in lactate in *dcerk^1^* (Figure 1B). Thus a significant portion of glucose metabolized by the glycolytic pathway was converted to lactate by the action of lactate dehydrogenase. The above results suggest that the increased reliance on glycolysis by *dcerk^1^* compensates for their decreased ability to produce ATP through oxidative phosphorylation.

### Increased activation of AKT is required for survival of *dcerk^1^* mutant flies

**Figure 3:**
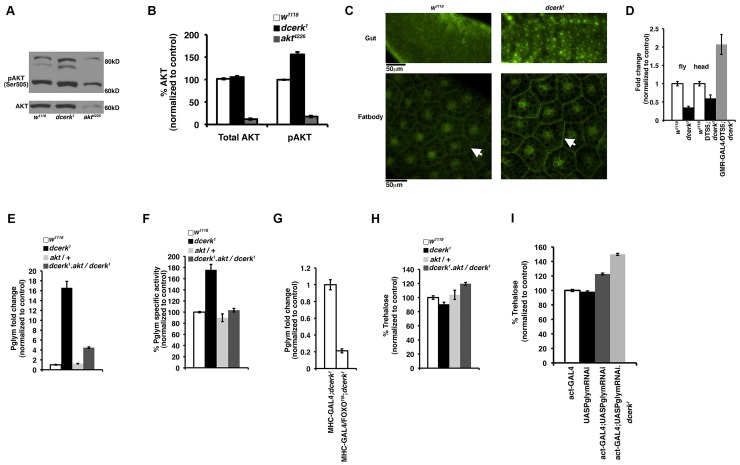
Increased glycolytic flux in *dcerk^1^* compared to *w^1118^* due to activation of Pglym and downstream glycolytic enzymes through AKT. (A). Total AKT and phospho AKT are monitored in control and mutant flies by Western blots. One band is observed for total AKT and it is not significantly different between *w^1118^* and mutant. Two major bands and one minor band are observed for phospho AKT and these are increased in *dcerk^1^* compared to *w^1118^*. The blots also show that total AKT and phospho AKT are both reduced significantly in the hypomorphic allele *akt^4226^* used in this study. (B). Quantification of total AKT and phospho AKT levels. Densitometric scanning of total AKT and phospho AKT bands from Western blots was carried out. The phospho AKT graph represents the average obtained after combining the values from all three bands observed on blots. The values were normalized to *w^1118^* taken as 100%. (C). tGPH staining is monitored in control (upper left panel) and *dcerk^1^* (upper right panel) adult midgut. The lower left panel shows tGPH staining in control while the lower right panel shows that in mutant larval fat body. There is increased tGPH staining at the cell membranes (compare arrows) indicating increased PIP3 levels and therefore increased PI3K activity. In addition to plasma membrane, nuclear tGPH is also increased in *dcerk^1^* gut since it has been reported that nuclear tGPH is also visible in many cell types when PIP_3_ levels are increased in cells. Midguts from 15–20 flies were visualized in each experiment and three independent experiments were carried out. For visualizing tGPH in fat body, 15–20 early third instar larvae were used in each experiment and three independent experiments were carried out. Scale bar represents 50 µm. (D). FOXO transcript level is measured by QPCR and shows a 60% reduction in in *dcerk^1^* flies compared to *w^1118^*. FOXO transcript level increases in the mutant heads when proteosomal degradation is inhibited by overexpressing DTS5 subunit in *dcerk^1^* photoreceptors compared to *w^1118^* or DTS5 without driver in *dcerk^1^* head samples. (E, F). Transcript level (E) and enzyme activity (F) of Pglym are significantly decreased when AKT activity is genetically reduced in *dcerk^1^* suggesting AKT mediates Pglym activation in *dcerk^1^*. (G). Overexpression of constitutively active FOXO in the muscles (MHC-GAL4) significantly decreases whole body Pglym transcript level. n = 3, error bars represent standard deviation. (H). Reduction of AKT in *dcerk^1^* increases total body trehalose level. Trehalose level is calculated per mg of protein and normalized to *w^1118^* level. n = 3, error bars represent standard deviation. (I). While actin GAL4 driver or UASPglymRNAi transgene individually have no effect on trehalose level, combining them increases whole animal trehalose by 20% in *w^1118^* and 50% in *dcerk^1^*. n = 3, error bars represent standard deviation.

Since *dcerk^1^* mutants show specific changes in glycolysis and differentially expressed genes in our transcriptional profiling data contained FOXO binding sites, we decided to test if AKT, a central modulator of metabolism and upstream regulator of FOXO was altered in *dcerk^1^*. AKT is a master regulator of survival, proliferation and cellular metabolism [32], [33]. AKT activation requires TOR dependent phosphorylation of Ser-473 (Ser-505 in *Drosophila*) at the C-terminus [34], [35]. We measured AKT activation in *dcerk^1^* flies by measuring the level of Ser-505 phosphorylated AKT protein by Western analysis. Phosphorylated AKT (seen as two major and one minor band) was increased while total AKT level was not different from *w^1118^* (Figure 3A). The blot also shows reduced levels of total and phospho AKT in *akt^4226^*, a hypomorphic allele of AKT used in this study [36]. Densitometric scanning of Western blots shows about 50% increase in pAKT level in the *dcerk^1^* mutant (Figure 3B). To test if activity of the upstream activator PI3K is increased, we used an *in vivo* reporter, which contains the PH domain of general receptor for phosphoinositide fused to GFP (tGPH) [37]. tGPH is increased in the cell membrane when PI3K activation increases PIP_3_ level. We observed an increased membrane association of tGPH in *dcerk^1^* compared to control in the adult mid gut (Figure 3C, top panel) and larval fat body (Figure 3C, bottom panel). These results suggest increased PI3K activation leads to increased AKT activation in *dcerk^1^*. A likely reason for the increased activation of PI3K could be the increased availability of PIP_2_ in the *dcerk^1^* mutant [10].

AKT mediates many of the alterations in metabolism through regulation of FOXO and in *Drosophila* there is a single homolog of the FOXO transcription factor family [38]–[41]. To test the status of FOXO, we measured FOXO transcript level in mutant flies and found that it was significantly decreased (Figure 3D). AKT activation in mammalian cells has been shown to lead to low FOXO level due to ubiquitination and proteosomal degradation of FOXO [42]. To address this possibility in *Drosophila*, we reduced ubiquitin mediated proteosomal degradation in *dcerk^1^* using a dominant temperature sensitive (DTS) mutation, DTS5 that affects the β6 proteosomal subunit [43]. Since ubiquitous expression of DTS5 resulted in lethality, we overexpressed DTS5 in photoreceptors using the GMR GAL4 driver and measured FOXO transcript level in heads. If FOXO is targeted for degradation in *dcerk^1^*, then expressing DTS5 should result in restoration of FOXO level and this is indeed the case (Figure 3D). Thus increased activation of AKT leads to downregulation of FOXO transcription factor in *dcerk^1^* mutant flies.

To evaluate the importance of increased AKT activity in *dcerk^1^* homozygous mutants, we generated *akt^4226^*, *dcerk^1^* double homozygotes. While *dcerk^1^* or *akt^4226^* homozygous flies alone are viable, double mutants are early larval lethal. Hence, compromised AKT function was incompatible with survival of *dcerk^1^* flies.

### Metabolic reprogramming in *dcerk^1^* involves increased glycolytic flux through activation of phosphoglycerate mutase and downstream glycolytic enzymes

To test if AKT may be required for the increased glycolytic flux in mutants, we generated *dcerk^1^* flies with reduced AKT function wherein one wild type copy of AKT was replaced with the hypomorphic allele of AKT. *dcerk^1^* flies heterozygous for *akt ^4226^* (referred to as *dcerk^1^.akt/dcerk^1^*) survive to adulthood and we have used them in subsequent experiments for evaluating the effects of reducing AKT function in *dcerk^1^*. The data from our studies with Pglym are shown here since it showed significant increase in both transcript and enzyme activity in mutant flies (Figures 3E, 3F). *dcerk^1^.akt/dcerk^1^* flies showed reduction in Pglym transcript and enzymatic activity. *akt^4226^*/+ flies were also tested in the above experiments and they did not differ significantly from *w^1118^* control suggesting that heterozygous state of AKT alone was not responsible for the changes (Figures 3E, 3F). Densitometric scanning of Western blots reflects a 50% decrease in total AKT and 30% decrease in pAKT levels in the *akt^4226^*/+ (Figures S2A, S2B). As mentioned earlier, AKT alters metabolic balance through downregulation of FOXO. We tested its involvement in regulating Pglym, by overexpressing FOXO™, a constitutively active form of the protein that localizes to the nucleus [44]. We expressed FOXO™ using a muscle driver, myosin heavy chain (MHC)-GAL4 and analyzed whole body Pglym transcript level [45]. Indeed, FOXO™ overexpression in the muscles resulted in significant decrease in Pglym transcript (Figure 3G), suggesting Pglym regulation is FOXO dependent.

The measurement of steady state levels of glucose (mammals) and trehalose (*Drosophila*) is an indicator of glycolytic utilization of sugars for energy metabolism. Therefore, we tested if AKT is required for maintaining normal trehalose levels in *dcerk^1^* mutants. Trehalose levels in fly extract of *dcerk^1^* were 90% of the control flies, whereas a reduction of *akt dosage* in *dcerk^1^* mutants resulted in an increase in trehalose (Figure 3H). Circulating trehalose levels also followed a similar trend as the whole body trehalose measurements in these flies (data not shown). We then globally reduced Pglym expression by ubiquitous RNAi mediated knockdown using the actin-Gal4 driver (Figure S3). Notably, this reduction resulted in semi lethality with 40% of the expected number of flies reaching adulthood in *dcerk^1^*. Trehalose levels increased by about 20% in *w^1118^* and by 50% in *dcerk^1^* when Pglym levels were reduced (Figure 3I). Hence, our results suggest increased glycolytic flux through Pglym is not only important for survival but also for maintaining near physiological levels of trehalose in *dcerk^1^* mutants.

### Metabolic adaptation in *dcerk^1^* involves increased mobilization of TAG through novel lipases, CG8093 and CG6277

Our experiments thus far suggest that *dcerk^1^* flies attempt to adapt to reduced energy availability. Triacylglycerol (TAG) is one of the major energy reserves, which is stored as cytoplasmic lipid droplets primarily in the adipose tissue [46]. In *Drosophila*, in addition to fat body cells, other tissues, most notably, the gut, oocytes, larval imaginal discs and oenocytes also accumulate TAG as intracellular lipid droplets [47]. During times of energy need such as starvation, stored TAG is hydrolyzed from these depots by activation of lipolytic mechanisms [48]. Fatty acids generated via lipolysis by lipases can be used for energy production through β-oxidation. Stored lipids are transported through the hemolymph as lipoprotein particles called lipophorins. So, we decided to test if energy rich TAG stores are altered in *dcerk^1^* and if so, does the AKT/FOXO pathway play a role in this process.

**Figure 4:**
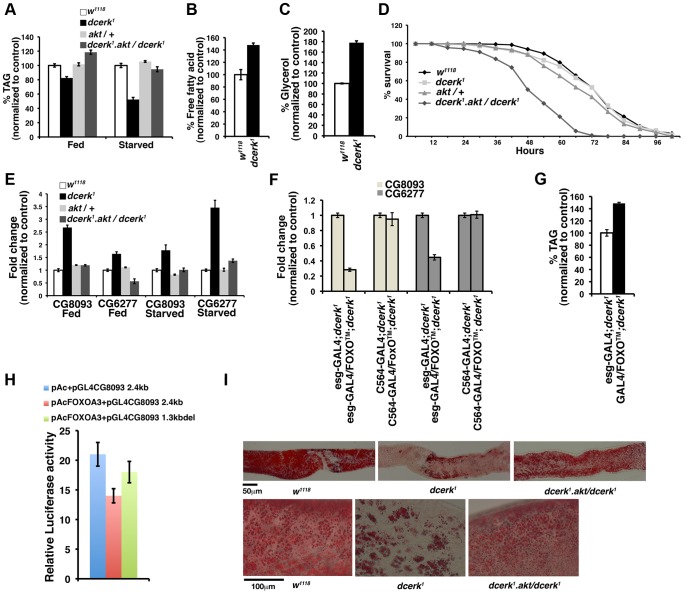
CG8093 and CG6277 are novel lipases in the gut activated by AKT/FOXO in *dcerk^1^*. (A). Relative TAG level in ad libitum fed *dcerk^1^* is 80% of *w^1118^* and increases to 120% when AKT is reduced. Relative TAG level in 20 hr starved *dcerk^1^* is 50% of *w^1118^* and significantly increases when AKT is reduced. n = 3, error bars represent standard deviation. (B). Free fatty acids were measured in lysates prepared from starved *w^1118^* and *dcerk^1^* flies. Mutants show increased free fatty acids compared to *w^1118^*. (C). Glycerol was measured in lysates prepared from starved *w^1118^* and *dcerk^1^* flies. Mutant shows increased glycerol compared to *w^1118^*. (D). *w^1118^*, *dcerk^1^*, *akt*/+ and *dcerk^1^.akt/dcerk^1^* flies are subjected to starvation and the number of surviving flies is recorded at 6 hr intervals. 200 flies divided into 10 groups for each genotype are used in one experiment. Three independent experiments are performed and the average percentage survival relative to *w^1118^* is shown for each time interval. Reduction in AKT increases the starvation sensitivity of *dcerk^1^* flies. (E). QPCR analysis shows transcript levels of two lipases CG8093 and CG6277 (identified by microarray analysis) are increased in *dcerk^1^* relative to *w^1118^* both in the fed and 20 h starved state. Both transcript levels are decreased upon reduction of AKT in *dcerk^1^*. n = 3, error bars represent standard deviation. (F). Overexpression of constitutively active FOXO in the midgut decreases whole animal transcript levels of CG8093 and CG6277 while fat body specific expression does not significantly affect them in *dcerk^1^*. FOXO overexpression with each driver is quantified by QPCR using FOXO primers and normalized. n = 3, error bars represent standard deviation. (G). Midgut specific overexpression of constitutively active FOXO increases whole animal TAG level in *dcerk^1^*. The TAG level in *dcerk^1^* expressing esg-GAL4 driver is taken as 100%. (H). Constitutive FOXO represses CG8093 in luciferase reporter assays. A 2.4 kb fragment (genomic region between 1773 bp to 4177 bp from transcription start site) containing FOXO core motifs upstream of CG8093 in a luciferase reporter vector or a 1.3 kb fragment (region between 2842 bp and 4177 bp) deficient of the FOXO motifs was cotransfected with constitutive dFOXO A3. FOXO represses transcription of the 2.4 kb fragment while the repression is less when the binding sites are deleted. (I). Dissected adult midguts stained with Oil Red O show significantly reduced lipid staining in starved *dcerk^1^* compared to *w^1118^*. *dcerk^1^* mutants with reduced AKT show comparatively more Oil Red O staining in the midgut. 15–20 guts are examined for each genotype in one experiment, which is carried out three times. Scale bar represents 50 µm. Magnified view of dissected adult midguts from *w^1118^* and *dcerk^1^.akt/dcerk^1^* show many Oil Red O stained puncta while they are significantly reduced in *dcerk^1^*. Scale bar represents 50 µm.

We measured whole animal TAG levels in ad libitum fed and 20 h starved *w^1118^* and *dcerk^1^* flies. The mutant flies showed 20% less TAG than *w^1118^* in the fed state and 50% less TAG in the starved state (Figure 4A). The decrease in TAG in the starved state is accompanied by increased free fatty acid and glycerol in whole fly extracts (Figures 4B, 4C). Since TAG levels were significantly altered in the starved state, we assessed the ability of *dcerk^1^* to survive starvation stress. As shown in Figure 4D, the susceptibility of mutant flies to starvation was similar to *w^1118^*. These results suggest that *dcerk^1^* mutant likely rely on increased TAG hydrolysis to survive starvation stress. To test if AKT mediated compensation allowed *dcerk^1^* to survive starvation, we subjected *dcerk^1^.akt/dcerk^1^* flies to starvation stress. These flies died at a faster rate than *dcerk^1^* showing reduction of AKT function in *dcerk^1^* led to starvation sensitivity (Figure 4D). TAG level in these flies was significantly higher than in *dcerk^1^*, both in the fed and starved states (Figure 4A). The starvation sensitivity of these flies despite increased TAG level suggested that they were unable to properly hydrolyze their TAG stores via lipolysis when AKT activity is reduced. Thus AKT dependent lipases may provide a critical function in *dcerk^1^* flies.

In order to identify lipases that are activated in *dcerk^1^*, we first tested transcript levels of known fat body lipases in *Drosophila*, which include Brummer (ATGL homolog), CG11055 (putative homolog of hormone sensitive lipase) and CG8552 (homolog of major triglyceride lipase in a related insect) [19], [48]. The transcript level of Brummer was decreased in *dcerk^1^* (0.5 fold compared to control) while that of the other two lipases did not show changes (data not shown). Thus traditional lipases were not upregulated for increased TAG utilization in *dcerk^1^*. We then pursued potential candidates from microarray studies. Our microarray analysis identified that amongst the 60 lipases that have been identified in *Drosophila*, expression levels of two genes, CG8093 and CG6277, that encode predicted TAG lipases were increased in *dcerk^1^*. CG8093 is an acid lipase of the α/β hydrolase family related to human lysosomal acid lipase while CG6277 is a neutral lipase showing homology to lipoprotein lipase and to pancreatic triacylglycerol lipase family. Both CG8093 and CG6277 contain the catalytic triad of Ser, His, Asp residues important for lipase activity. Earlier studies involving microarray analysis in *Drosophila* demonstrated that these lipases are repressed by high sugar and *in situ* hybridization studies showed they were expressed in the midgut [49]. Our QPCR results also showed that these genes were highly expressed in the midgut (Figure S4). To test if these lipases could facilitate TAG utilization in *dcerk^1^*, we first examined their transcript levels in fed and starved states (Figure 4E). Both genes were upregulated in *dcerk^1^*. This is an AKT mediated adaptive response since the transcript levels were downregulated when AKT is reduced in *dcerk^1^*. To evaluate the involvement of FOXO, we overexpressed FOXO™ using gut (esg-GAL4) and fat body (C564-GAL4) specific GAL4 drivers [50], [51]. Gut specific overexpression of FOXO™ decreased whole animal lipase transcripts while fat body GAL4 did not alter expression significantly in *dcerk^1^* (Figure 4F). Overexpression of FOXO™ in the gut also resulted in higher TAG level in *dcerk^1^* compared to control (Figure 4G).

The expression of these lipases could be regulated directly or indirectly by FOXO. Our earlier analysis identified FOXO binding sites upstream of CG8093 (Table S2) and thus could be a potential target of FOXO. To test if negative regulation of CG8093 by FOXO is mediated through these sites, we performed luciferase reporter assays. A 2.4 kb fragment containing two FOXO motifs from the regulatory region of CG8093 (pGL4CG8093 2.4 kb) and a fragment in which the motifs were deleted (pGL4CG8093 1.3kbdel) were cloned in a reporter vector containing the luciferase gene (pGL4). *Drosophila* S2 cells were cotransfected with constitutively active FOXO along with each of these constructs and luciferase activity was measured after 30 h [40]. Constitutively active FOXO suppresses the luciferase activity of pGL4CG8093 2.4 kb and this is considerably relieved in pGL4CG8093 1.3kbdel, which is deficient of the FOXO binding sites (Figure 4H). This suggests FOXO could act via these sites to regulate CG8093 transcription. Our search for FOXO binding sites was limited to 10 kb upstream of the microarray target genes and CG6277 was not identified in this analysis. The promoter region of CG6277 does not contain obvious FOXO binding sites suggesting transcriptional regulation either takes place through the interaction with other transcription factors or is indirectly mediated through FOXO dependent induction of a transcriptional repressor protein. A possible candidate could be sugarbabe, which encodes a zinc finger protein. An earlier study that analyzed sugar dependent gene expression profiles in *Drosophila* larvae identified CG6277 could be negatively regulated by sugarbabe [49]. Our analysis of sugarbabe transcript in *dcerk^1^* mutants suggests its level is low, which would be consistent with the observed upregulation of CG6277 transcript level. Also, analysis of FOXO binding sites identifies two potential regions within 10 kb upstream of sugarbabe (data not shown). Thus a possible mode of regulation could be FOXO activates sugarbabe, which in turn negatively regulates CG6277. Thus downregulation of FOXO in *dcerk^1^* could lead to activation of CG6277. These results suggest that increase in CG8093 and CG6277 through AKT/FOXO could lead to increased lipolysis and decreased TAG level in *dcerk^1^*.

To examine whether increased hydrolysis of fat from the gut did occur as a result of increased lipase activity, guts isolated from *w^1118^*, *dcerk^1^* and *dcerk^1^.akt/dcerk* ^1^ were stained with the neutral lipid dye Oil Red O under starved conditions (Figure 4I). Oil Red O staining was evident in *w^1118^* gut while *dcerk^1^* showed little staining. Midgut isolated from *dcerk^1^.akt/dcerk^1^* also stained well with Oil Red O (Figure 4I). Oil Red O puncta representing lipid droplets were clearly visible in *w^1118^* and *dcerk^1^* with reduced AKT but were substantially decreased in *dcerk^1^* midgut (Figure 4I, bottom panel).

To ensure that changes in gene expression and increased utilization of lipid in the gut are not to due to compromised gut integrity or defective epithelial renewal, we tested if *dcerk^1^* gut were morphologically different from *w^1118^* gut. We observed that gut length, width and ability to ingest food were not different between control and mutant flies (data not shown). Epithelial integrity and epithelial renewal were also assessed as described in Figure S5 and these data suggest that gut integrity or epithelial renewal in the gut is largely uncompromised in *dcerk^1^*.

### Midgut specific knock down of CG8093 and CG6277 results in TAG accumulation, starvation sensitivity and cardiac defects

**Figure 5:**
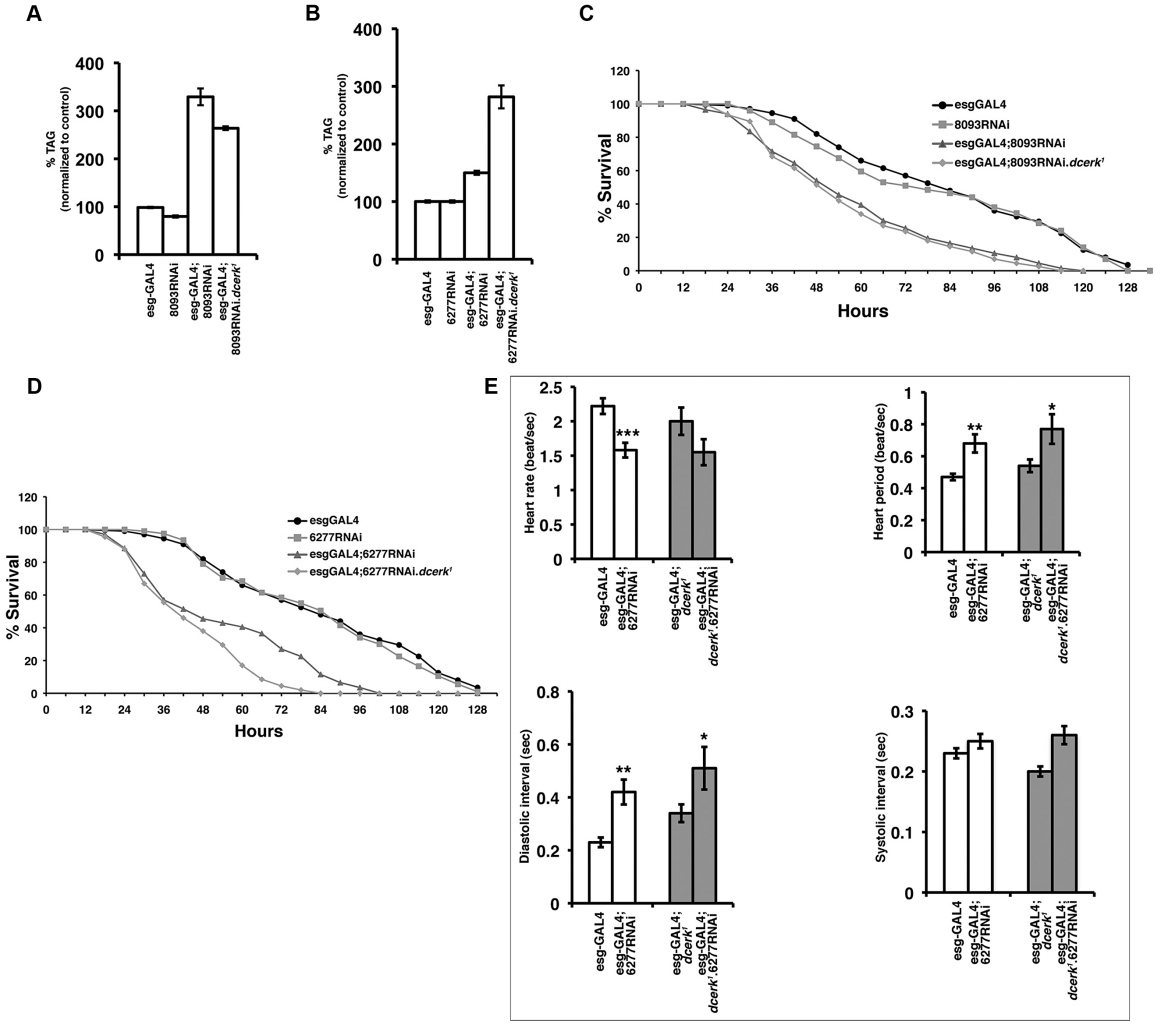
Knockdown of CG8093 and CG6277 in the midgut increases whole body TAG, starvation sensitivity in *w^1118^* and *dcerk^1^* flies and knockdown of CG6277 also leads to cardiac defects. (A). Midgut specific reduction in CG8093 increases whole body TAG level to 325–350% in control *w^1118^* flies and about 275% in *dcerk^1^* mutants. The TAG level of driver is taken as 100%. n = 3, error bars represent standard deviation. (B). Midgut specific reduction in CG6277 increases whole animal TAG to 150% in control *w^1118^* flies and about 275%–300% in *dcerk^1^* mutants. The TAG level of driver is taken as 100%. n = 3, error bars represent standard deviation. (C). Reduction in CG8093 lipase increases the starvation sensitivity of wild type and *dcerk^1^* flies. CG8093 RNAi flies, esgGAL4 driver flies, 8093 RNAi combined to esgGAL4 in wild type and *dcerk^1^* are subjected to starvation and the number of surviving flies is recorded at 6 hr intervals. 200 flies divided into 10 groups for each genotype are used in one experiment. Three independent experiments are performed and the average percentage survival is shown for each time interval. (D). Reduction in CG6277 lipase increases the starvation sensitivity of wild type and *dcerk^1^* flies. CG6277 RNAi flies, esgGAL4 driver flies, 6277 RNAi combined to esgGAL4 in wild type and *dcerk^1^* are subjected to starvation and the number of surviving flies is recorded at 6 hr intervals. (E). Quantification of cardiac function parameters in RNAi knockdown of CG6277 in wild type and *dcerk^1^*. Knockdown results in decreased heart rate in wild type flies and a similar trend in *dcerk^1^*, increased heart period in both backgrounds, longer diastolic interval in control and mutant and no significant change in systolic interval. n = 11–13 for each group. Bar represents standard error of mean and statistical significance was determined using Student's t test; * denotes P< = 0.05-0.01, ** is P< = 0.01-0.001 is and *** denotes P< = 0.001-0.0001.

Our data suggest that increased expression of CG8093 and CG6277 leads to increased lipolysis and a concomitant decrease in TAG level in *dcerk^1^*. To ascertain this observation, we asked if impairing the function of these lipases in *dcerk^1^* would lead to accumulation of TAG in the mutant. We attempted to reduce their levels by ubiquitous expression of UAS-CG8093 RNAi and UAS-CG6277 RNAi constructs in *dcerk^1^*. However these conditions resulted in lethality of both wild type and *dcerk^1^* flies. Since these lipases are expressed predominantly in the gut, we next drove the RNAi constructs using the midgut specific driver esg-GAL4 and were able to obtain adult flies. RNAi resulted in 80% reduction in the transcript levels of the lipases in control and *dcerk^1^* (Figure S6). Midgut specific knockdown of each of these lipases resulted in significant increase in whole animal TAG levels in *dcerk^1^* (Figures 5A, 5B). Reduction of these lipases also resulted in increased TAG in control flies (Figures 5A, 5B). Gut specific reduction of the lipases also resulted in increased sensitivity to starvation in both wild type and mutant background compared to GAL4 driver and RNAi control flies (Figures 5C, 5D). Staining of guts isolated from knockdown flies with Oil Red O showed increase in the number of Oil Red O puncta indicative of defective mobilization of lipid stores (Figure S7). These results collectively suggest that both lipases are important for utilization of TAG and maintenance of TAG homeostasis not only in the mutant but also in wild type flies. Additionally, the life span of wild type and *dcerk^1^* flies is significantly reduced upon gut specific knockdown of CG6277 (data not shown).

### Heart function in *dcerk^1^* mutants with lipase or Pglym knockdown

The heart has a high demand for energy and relies on fatty acids derived from lipolysis of lipoprotein bound TAG by lipases as energy substrates [52]. Cardiac dysfunction is linked to altered energy metabolism in many instances [53]. To test if the two lipases played a role in this process, we tested if their reduction in wild type flies compromised cardiac function using a variety of heart performance assays that have been developed in *Drosophila* [21]. Heart function was compromised in one-week old gut specific knockdown of CG6277 as manifested by decreased heart rate or increased heart period primarily due to an increased diastolic interval (Figure 5E). Knockdown of CG6277 in *dcerk^1^* mutant flies also resulted in increased heart period and a trend towards decreased heart rate (Figure 5E). One-week old *dcerk^1^* alone did not show significant changes in heart parameters, and only a slight trend towards a slower heart rate and dilation (increased systolic and diastolic diameters). While gut specific knockdown of CG8093 in wild type and in *dcerk^1^* flies did not show significant changes in most of the parameters tested for heart function, heart period is affected in *dcerk^1^* due to expansion of diastolic interval, pointing to the importance of this lipase for heart function in *dcerk^1^* flies (Figure S8).

Since the knockdown of lipases resulted in increased sensitivity to starvation and cardiac defects, we also tested the effects of downregulating the glycolytic gene, Pglym in sensitivity to starvation and cardiac functions in wild type and mutant backgrounds. Ubiquitious knockdown of Pglym results in a significant increase in sensitivity to starvation stress in wild type and *dcerk^1^* (Figure S9). Evaluation of various heart function parameters with Pglym knockdown flies in both wild type and *dcerk^1^* mutant backgrounds showed a decrease in diastolic and systolic diameters resulting in a somewhat constricted phenotype without significant changes in other parameters (Figure S9). This is reminiscent of cardiac constricted phenotype of *eas* mutants, which show aberrations in phosphatidylethanolamine synthesis and elevated SREBP activity [26].

### Failure of adaptive mechanisms in *dcerk^1^* mutants leads to cardiac defects and decreased life span

**Figure 6:**
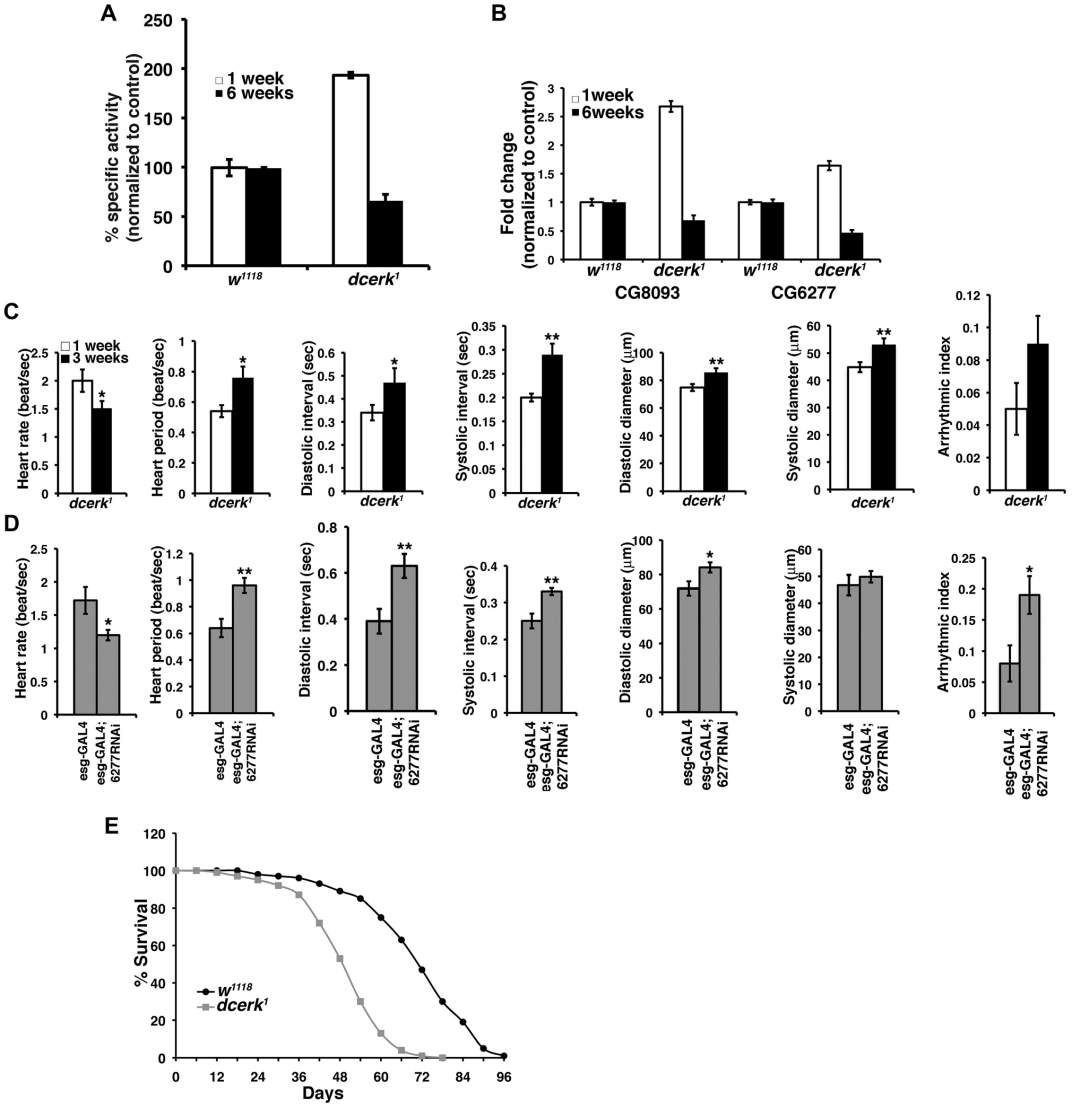
Failure of adaptive mechanisms leads to cardiac dysfunction and reduction in adult life span of *dcerk^1^*. (A). Measurement of Pglym enzyme activity in young (1 week) and aged (6 weeks) *w^1118^* and *dcerk^1^* flies shows a significant decrease in activity in older *dcerk^1^*. (B). Comparison of transcript levels of CG8093 and CG6277 by QPCR in 1week and 6 week old flies, reveals decrease in their transcripts in aged *dcerk^1^* flies. (C). Comparison of several cardiac performance indices in 1 and 3 week old esgGAL4 *dcerk^1^* flies reveals progressive decline in heart function with decreased heart rate, increased heart period and dilated heart chamber. n = 15, bar represents standard error of mean and statistical significance was determined using Student's t test; * denotes P< = 0.05-0.01, ** is P< = 0.01-0.001 is and *** denotes P< = 0.001-0.0001. (D). Knockdown of CG6277 in 3 week old wild type flies also shows decreased heart rate, increased heart period, dilated heart chamber and arrhythmia. n = 10, bar represents standard error of mean and statistical significance was determined using Student's t test; * denotes P< = 0.05-0.01, ** is P< = 0.01-0.001 is and *** denotes P< = 0.001-0.0001. (E). *dcerk^1^* have reduced adult life span compared to *w^1118^* flies. The survivorship curve of *dcerk^1^* and w*^1118^* represents data from 600 flies in duplicate.

To test if the adaptive mechanisms we observe in young *dcerk^1^* flies (less than one week) is sustained or fails with age, we tested for AKT activation and measured FOXO transcript level in aged *dcerk^1^* (6 weeks). We observe a decrease in AKT activation and 4-fold increase in FOXO transcript level in older flies (data not shown). Consequently, Pglym activity decreases significantly in old *dcerk^1^* flies (Figure 6A) leading to increased trehalose level (data not shown). Similarly, transcript levels of both lipases decrease with age (Figure 6B), leading to 50% increase in TAG level in old *dcerk^1^* (data not shown). These results suggest the efficiency of the compensatory mechanisms decrease with age in the mutant flies. The decompensation leads to deterioration in many indices of heart performance between young and three-week old *dcerk^1^* flies (Figure 6C), a time point at which we begin to observe noticeable decrease in transcript levels of the lipases in the mutant. These changes follow a similar trend as those observed when CG6277 lipase is knocked down in healthy three-week old wild type flies (Figure 6D). In both these situations in addition to heart rate and heart period changes, we also observe dilated heart chamber, perhaps due to increased dependence of heart function on this lipase with aging. We chose an interim period of three weeks because CG6277 lipase knockdown flies do not survive for six weeks. Thus, a decrease in CG6277 compromises cardiac function likely due to altered energy balance resulting from decreased TAG hydrolysis as observed in wild type flies when the transcript levels are reduced by RNAi or in aging *dcerk^1^* mutants when AKT mediated compensatory changes begin to fail. We then tested whether impaired mitochondrial function and age related pathology such as triglyceride accumulation and decline in cardiac function impacted the life span of *dcerk^1^*. While 90% of the mutants die by day 60, only 20% of the controls are dead by this time, suggesting the adult life span of the mutant flies is shortened (Figure 6E).

**Figure 7:**
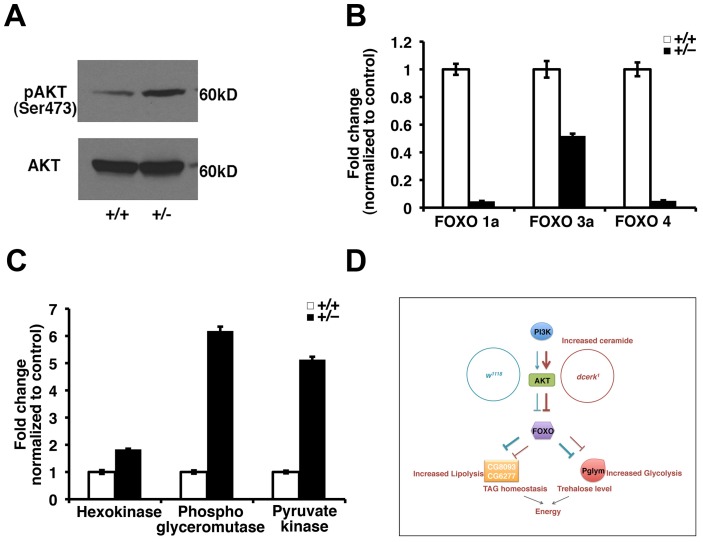
Activation of AKT, decrease in FOXO and activation of glycolytic genes in CERT heterozygous mice. (A). Total AKT and phosphoAKT levels are monitored in liver extracts from wild type (+/+) and CERT heterozygous (+/−) mice. PhosphoAKT level is increased in CERT heterozygotes while total AKT is not significantly changed. (B). Comparison of different FOXO in liver tissue of wild type (+/+) and CERT heterozygous (+/−) mice shows significant decrease in their transcript levels. (C). Comparison of glycolytic genes in skeletal muscle from wild type (+/+) and CERT heterozygous (+/−) mice shows greater than 5 fold increase in transcript levels of phosphoglycerate mutase and pyruvate kinase and 1.8 fold increase in hexokinase. (D). A simple graphical model depicting metabolic reprogramming in *dcerk^1^* due to increased AKT activation and decreased FOXO to maintain energy balance. *dcerk^1^* mutants show glucose utilization through increased glycolytic flux mediated by Pglym and downstream enzymes. They also show increased utilization of triglycerides mediated by CG8093 and CG6277.

We finally asked whether the adaptive mechanisms to increased ceramide identified here using *Drosophila dcerk* mutants are likely important in other scenarios where ceramide is increased. One example is the mouse ceramide transfer protein mutant, which accumulates ceramide and dies around embryonic day 11.5 [28]. These embryos show severe mitochondrial degeneration, decreased phospho AKT level and cardiac defects suggesting a likely failure of the adaptive mechanism. CERT heterozygous mice are viable, show mitochondrial damage and stress but not as severe as the homozygotes. We tested if our central results of AKT activation and decrease in FOXO level are observed in CERT heterozygous mice. Indeed, we see increased phosphoAKT level and decreased levels of different FOXO transcripts (Figures 7A, 7B). While results from liver extracts are shown here, down regulation of FOXO is also observed in other tissues such as intestine and skeletal muscle. We also observe an increase in transcripts of phosphoglycerate mutase and pyruvate kinase, suggesting a likely increase in glycolysis in these animals (Figure 7C). We then tested some of the mammalian lipase family members that showed homology to CG8093 (LIPA, LIPF, LIPM) and CG6277 (pancreatic lipoprotein 1, pancreatic lipoprotein related protein, lipoprotein lipase, endothelial lipase) but could not detect changes in transcript levels. The lack of validation of targets in triglyceride utilization in the mice suggests that CERT heterozygotes may not be the ideal model for lipolytic mechanisms and also reflects the complexities involved in lipid sensing in the gut and coordination with other organs in the mammalian systems.

## Discussion

In this study, we have attempted to understand how *dcerk^1^* flies survive and adapt to increased ceramide level, which disrupts several of the physiological metabolic pathways. Our results collectively suggest that in the presence of increased ceramide, the most important coping strategies for organismal survival are geared towards energy production through metabolic remodeling involving upregulation of genes that function in breakdown of dietary nutrients, increased glycolysis and utilization of stored fat in the gut. This is due to increased activation of PI3K/AKT/FOXO pathway and subsequent activation of novel downstream effectors (summarized in a model, Figure 7D). Failure of this compensatory mechanism (due to aging or genetic reduction) leads to TAG accumulation, increased starvation sensitivity and cardiac abnormalities.

While *dcerk^1^* flies are viable, genetic reduction of AKT results in lethality showing survival of *dcerk^1^* is dependent on increased activation of AKT. AKT activation leads to increased glycolytic flux in the mutants. Increase in lactate level in *dcerk^1^* (in the presence of sufficient oxygen) is reminiscent of aerobic glycolysis observed in normal proliferating cells as well as in cancer cells. In normal proliferating cells, increasing glycolytic intermediates has been proposed to support macromolecular synthesis while a similar phenomenon called Warburg effect is observed in cancer cells where glycolysis is increased to provide energy to cells defective in mitochondrial respiration [54], [55]. AKT does not activate its traditional targets of glycolysis such as hexokinase and phosphofructokinase in *dcerk^1^* but rather Pglym and downstream glycolytic enzymes. Historically Pglym has not been viewed as a rate-limiting step in glycolysis although studies have suggested its importance in heart, phagocytic cells and cancer cells [56], [57]. Thus, within the glycolytic pathway, control of metabolic flux can be achieved by exerting control on different enzymes depending on cellular conditions and Pglym could be an important node in normal proliferating as well as in stressed cells. Addition of ceramide analogs has been reported to inhibit AKT activation without affecting upstream signaling events [58], [59]. Possible mechanisms include activation of protein phosphatase 2A, which could dephosphorylate AKT and interference with membrane recruitment of AKT by atypical protein kinase C [59], [60]. These effects could in part be due to use of pharmacological amounts of ceramide analogs, which likely do not mimic endogenous ceramide. Nevertheless, these effects could be negated by overexpression of constitutively active AKT [59]. How is AKT activation achieved in *dcerk^1^*? While several mechanisms could contribute to AKT activation, an idea we favor is that more PIP_2_ is available for PI3K since utilization of PIP_2_ by PLC is severely decreased due to its degradation in *dcerk^1^* [10]. While we see an increase in tGPH suggesting an increase in PIP_3_, we have not been able to quantify PIP_3_ level by mass spectrometry. However, PIP_2_ level shows 60% increase in *dcerk^1^* compared to control [10].

Studies in *Drosophila* have highlighted the role of Brummer lipase (ATGL homolog) in fat storage and mobilization from the fat body and in mice have shown ATGL mediated lipolysis is important for PPARα-PGC1 complex activity and heart function [19], [61]. Magro, a target of the nuclear receptor DHR96, hydrolyzes dietary lipids in the gut as well as stored cholesterol esters highlighting the importance of the *Drosophila* gut in maintaining TAG and cholesterol homeostasis [62], [63]. In mammalian systems, understanding of lipid sensing by the intestine and its coordination with other organs such as the liver, brain and the signaling pathways in these organs that alter glucose and energy homeostasis are just beginning to be explored [64]. Our results suggest utilization of stored TAG from the gut is an important mechanism in times of energy need such as starvation stress and CG8093 and CG6277 are critical targets of AKT/FOXO in this process. In a *Drosophila* larval model of type 2 diabetes that is accompanied by high sugar and TAG levels, gene expression changes reveal that CG8093 and CG6277 are both significantly downregulated (15 and 5 fold respectively) suggesting these lipases could have a role in hypertriglyceridemia associated with diabetes [65]. Metabolic control of lipolysis has been primarily studied at the level of nutrient-sensing signal transduction cascades rather than transcriptional regulation and hence not much is known about the regulation of lipases by FOXO. In *Drosophila*, dFOXO has been shown to directly regulate an acid lipase, dlip4 [66]. Recent studies show a hormone dependent module consisting of salt induced kinase SIK3, the histone deacetylase HDAC4, which regulate FOXO activity in lipolysis [67]. In *C.elegans*, fat mobilization through induction of a triglyceride lipase K04A8.5, a target of DAF16 increases longevity [68]. In mammalian systems, FOXO has been shown to regulate lipolysis by either directly controlling expression of ATGL or through PPARγ [69]. A forkhead factor, FKHR has been shown to upregulate lipoprotein lipase expression in the skeletal muscle [70]. While FOXO binding sites have been identified in the promoter region of lipoprotein lipase, that it can modulate its expression directly has yet to be demonstrated [71]. Further understanding of the role of FOXOs in different organs will allow the exploration of the therapeutic value of targeting FOXO in metabolic diseases.

Many recent studies show sphingolipids could be relevant to changes in carbohydrate and fat levels. In *Drosophila*, overexpression of glucosylceramide synthase that catalyzes synthesis of glucosylceramide increases TAG and carbohydrate levels while a reduction causes decrease in fat storage [72]. *Drosophila* ceramide synthase mutants also show reduction in fat storage [73]. In mammalian systems, mice lacking sphingomyelin synthase and ceramide kinase (both of which show increase ceramide levels) are resistant to high fat diet induced obesity [74], [75]. It would be interesting to test if these animals show increased lipolytic mechanisms based on our observations in *Drosophila*. Our results with the *dcerk* mutant presented here as well as other mutants of *Drosophila* sphingolipid enzymes (our unpublished observations) suggest that an increase in ceramide in one cellular compartment eventually leads to an increase in ceramide in the mitochondria suggesting that a central effect of ceramide accumulation in an organism is impairment of mitochondrial function. This impact on mitochondria is also being validated in recent mice knock out studies such as the sphingomyelin synthase 2 and CERT mutants, which show mitochondrial dysfunction [16], [28].

The mechanisms outlined here could not only mediate adaptation to ceramide, but could be important responses to reduced energy availability. The ‘protective effects’ of the adaptive mechanisms revealed here suggest that they have potential therapeutic implications in ceramide mediated stress involving decreased energy leading to high sugar and TAG levels, ultimately resulting in cardiac dysfunction. Also, importantly, the identification of novel downstream targets in the AKT/FOXO pathway opens the possibility of new therapeutic targets in treatment of cardiac dysfunction, hyperglycemia and hypertriglyceridemia.

## Materials and Methods

### Fly stocks and husbandry

*Drosophila* stocks were raised on standard corn meal agar and maintained at 25°C. 2–7 day old flies were used in all experiments unless otherwise indicated. *Akt^4226^*, actin-GAL4 driver, MHC-GAL4 driver and tGPH flies were obtained from Bloomington Stock Center (Indiana University). UAS-FOXO was a gift from Marc Tatar (Brown University, Providence), esg-Gal4 from Norbert Perrimon (Harvard Medical School, Boston) C564-GAL4 flies from Neal Silverman (UMass, Worcester) and DTS flies were from Eric Baerahcke (UMass, Worcester). UAS RNAi lines for CG8093, CG6277 and Pglym were obtained from VDRC (Vienna). *Akt^4226^* was recombined to *dcerk^1^* to obtain *dcerk^1^.akt/dcerk^1^* flies. UASRNAi lines for CG8093, CG6277 and Pglym on the third chromosome were recombined to *dcerk^1^* to obtain *dcerk^1^*.UASCG8093RNAi, *dcerk^1^*.UASCG6277RNAi and *dcerk^1^*.UASPglymRNAi lines. UAS-FOXO, esg-GAL4, actin-GAL4, C564-GAL4 and MHC-GAL4 drivers on second chromosome were used.

### Microarray analysis

*dcerk^1^* and *w^1118^* flies were collected, and RNA was extracted using TRIzol (Invitrogen) and purified on RNeasy columns (QIAGEN). All samples were prepared in triplicate to facilitate subsequent statistical analysis. Purified RNA was sent to Expression Analysis (Durham, NC), where it was labeled and hybridized to Affymetrix Drosophila 2.0 microarrays. The details of the analysis are described in Supplemental Materials and Methods (Text S1).

### Metabolic profiling

*dcerk^1^* and *w^1118^* (100 flies each, in triplicate) were collected and frozen. The samples were prepared and analyzed by LC/MS, LC/MS/MS and GC/MS platforms by Metabolon (Durham, NC).

### ATP, TAG, trehalose, glycerol and free fatty acid measurements

TAG measurement was carried out using the Serum triglyceride determination kit (TR0100; Sigma, St. Louis, MO). Total body and circulating trehalose were measured using a glucose reagent (GAGO-20, Sigma) followed by porcine kidney trehalase (Sigma, T8778). ATP measurements were carried out using an ATP assay kit (Calbiochem). Glycerol and free fatty acid were determined using coupled enzyme assay kits (Zenbio, NC). Detailed methodology is provided in Supplemental Materials and Methods (Text S1).

### Starvation assay

In each experiment, 10 batches of 20 flies for each genotype were transferred to vials containing a small piece of sponge soaked with water. Dead flies were counted at 6 h intervals.

### Longevity assay

Newly eclosed adult w*^1118^* and dcerk*^1^* were collected over a 24 hr period and divided into batches of 20 flies per vial. Flies were transferred to fresh food vials and scored for survival every 48 h.

### Fly heartbeat analysis

Semi-intact fly heart preparations were used for heart parameter measurements according to published protocols [20]. High speed 1 min movies were taken at a rate of >100 frames per second using a Hamamatsu CCD camera on a Leica DM LFSA microscope with a 10× dipping immersion lens. The images were processed using Simple PCI imaging software (Hamamatsu Inc.). Cardiac parameters including heart rate, heart period, diastolic and systolic intervals, diameters, fractional shortening and arrhythmic index were generated using a MatLab-based image analysis program.

### Adult gut and larval fat body staining

For Oil red O staining, midguts from 2–7 days old adult flies were dissected and fixed in 4% paraformaldehyde/PBS for 30 min at room temperature. The midguts were washed four times in PBS and incubated for 20 to 30 min in 0.1% Oil Red O stain (prepared fresh in isopropanol∶water and passed through a 0.45 µm syringe filter). Midguts were washed three times with PBS, and mounted in 20% glycerol/PBS. The guts were imaged immediately using Differential Interference Contrast microscopy in a Nikon Eclipse E-600 microscope. For visualization of tGPH, adult midgut or fat body from early third instar larvae were dissected in PBS and visualized immediately using Nikon Eclipse E-600 microscope and NIS-Elements Imaging Software.

### Quantitative RT-PCR

Total RNA was extracted using TRIzol reagent and cDNA was synthesized using the SuperScript III first–Strand Synthesis kit (invitrogen). Real time PCR was performed in the ABI PRISM 7000 sequence Detection System (Applied Biosystems) using SYBR Green Supermix (Invitrogen). Reactions were normalized to RP49 levels. Detailed methodology and primers used are provided in Supplemental Materials and Methods (Text S1).

### Luciferase assays

#### Constructs

A 2.4-kb fragment containing two FOXO core motif sites WWAACA in the putative regulatory region of CG8093 (chr2R 10725084 – 10727488) was identified. This fragment was cloned in pGL4-basic vector, which has a firefly luciferase reporter gene (Promega). The fragment was amplified by PCR from genomic DNA of flies using the following primers F5′GGGGGCTAGCGACAGCTACTTACATCCAAGCAATA3′ and R5′GGGGCTCGAGCAACGAGCTCAAATTGGGTAAGA-3′and cloned between NheI and XhoI sites (pGLCG8093 2.4). The FOXO deficient fragment was generated by using primers F5′GGGGGCTAGCC ACGAGAAATGTCAGGAAGTAATG3′ and R5′GGGGGCTAGCCACGAGAAATGTCAGGAAGTAATG3′ and cloned in pGL4 vector between NheI and XhoI sites (pGLCG8093 1.3kbdel). The empty pAc5.1 vector and constitutively active version of dFOXO in pAc5.1 vector were gifts from Drs. Oscar Puig and Jaakko Mattila.

#### Cell culture and transfection

*Drosophila* Schneider (S2) cells were grown in Schneider medium with 10% heat-inactivated fetal bovine serum, penicillin, and streptomycin in 25°C in suspension. For transfection, 1×10^5^ S2 cells were plated per well in a 96 -well plate in triplicates. Cells were transfected using Effectene transfection reagent (Qiagen) according to the manufacturer's instructions. For transfections, 100 ng of expression vector (pAcFOXOA3 or pAc5.1) and 100 ng of reporter vector (pGLCG8093 2.4, pGLCG80931.3del) along with renilla plasmid (20 ng) were used. After 30 h of transfection, luciferase assays were performed using the dual-glow luciferase assay system as per manufacturer's protocol (Promega). Each experiment was repeated three times.

### Immunoblotting

Flies were crushed in SDS-sample buffer; the extracts were centrifuged and separated by SDS-PAGE followed by Western blotting using antibodies to AKT (1∶1000, Cell Signaling) and phospho Drosophila AKT Ser505 (1∶1000, Cell Signaling).

### Measurement of activity of glycolytic enzymes

Soluble extracts were prepared from approximately 1000 flies per batch using different buffers for different enzymes and spectrophotometric assays were carried out as detailed in Supplemental Materials and Methods (Text S1).

### Assays for mitochondrial oxidative phosphorylation complexes

Mitochondria were isolated from approximately 1000 flies per batch. The activity of each of the five complexes was determined spectrophometrically as detailed in Supplemental Materials and Methods (Text S1).

## Supporting Information

Figure S1 **Ceramide levels increase while ceramide 1-phosphate levels are not significantly affected in** *dcerk^1^* **mutant fly extracts compared to** *w^1118^* **.** (A). d14 long chain base ceramide with different fatty acids, C20:0, C22:0, C24;1 and C24:0 are estimated by mass spectrometry in sphingolipid enriched fractions prepared from *w^1118^* and *dcerk^1^* flies. The amount of ceramide is calculated based on total carbon content and then normalized to *w^1118^*. All ceramides show significant increase (P< = 0.001-0.0001) in mutant compared to *w^1118^*. n = 3, error bars represent standard deviation. (B). d14 long chain base ceramide 1-phosphate (C1P) with fatty acids C20:0, C22:0 and C24:0 are estimated by mass spectrometry. A 20% decrease in C24:0 C1P is observed in mutant relative to *w^1118^*. n = 3, error bars represent standard deviation.
(TIF)

Figure S2 **Western blot of total AKT and phospho AKT level in** *akt^4226^* **/+ heterozygotes.** (A). Western blot of total AKT and phospho AKT in *w^1118^*, *dcerk^1^* and *akt^4226^*/+. (B). Quantification of the blots by densitometric scanning shows a 50% decrease in total AKT and 30% reduction in pAKT in the heterozygote. n = 3.
(TIF)

Figure S3 **Ubiquitous RNAi knockdown of Pglym in** *w^1118^* **and** *dcerk^1^* **.** Combining actin GAL4 driver with UASPglymRNAi transgene results in significant reduction in Pglym transcript level in *w^1118^* and *dcerk^1^*. n = 3, error bars represent standard deviation.
(TIF)

Figure S4 **CG6277 and CG8093 show high expression in the adult midgut.** QPCR analysis is carried out using total RNA extracted from adult head and dissected adult midgut from *w^1118^*. CG6277 shows approximately 20-fold increase in transcript level in the midgut compared to head while CG8093 shows a 5 fold increase suggesting these genes are highly expressed in the adult midgut. n = 3, error bars represent standard deviation.
(TIF)

Figure S5 **Gut epithelial integrity and epithelial renewal is not compromised in** *dcerk^1^* **mutants.** A. *w^1118^* and *dcerk^1^* gut are stained with Armadillo (β-Catenin homolog), an important component of adherens junctions. The nuclei are stained with DAPI. Armadillo staining is not significantly different between *w^1118^* and *dcerk^1^* suggesting epithelial integrity is not compromised in the mutant. Scale bar represents 25 µm. B. This panel shows *w^1118^* and *dcerk^1^* midguts expressing esg-GAL4 GFP, which marks enteroblasts and intestinal stem cells involved in epithelium renewal. GFP staining is not significantly different between control and mutant guts. The guts are also stained with DAPI. Scale bar represents 50 µm. C. Survival of *dcerk^1^* is not significantly different from *w^1118^* upon ingestion of *Pseudomonas entomophila*. Increased epithelial renewal is required in the gut to make up for damage caused by oral pathogens and flies with low epithelial renewal succumb to bacterial infection earlier than control flies. However, *dcerk^1^* flies died at a similar rate as controls when fed *Pseudomonas entomophila* suggesting epithelial renewal in response to gut damage in *dcerk^1^* is not compromised.
(TIF)

Figure S6 **Gut specific knockdown of CG8093 and CG6277 in** *w^1118^* **and** *dcerk^1^* **.** Combining expression of esg-GAL4 driver and UASCG8093RNAi transgene effectively reduces CG8093 transcript level in *w^1118^* and *dcerk^1^* flies. Similarly, combining expression of esg-GAL4 driver and UASCG6277RNAi transgene effectively reduces CG6277 transcript level in *w^1118^* and *dcerk^1^* flies. n = 3, error bars represent standard deviation.
(TIF)

Figure S7 **Knockdown of CG8093 and CG6277 in** *w^1118^* **results in increased Oil Red O puncta in the midgut.** Dissected adult midguts stained with Oil Red O show significantly increased staining in RNAi knockdown in *w^1118^* compared to RNAi controls. Oil Red O puncta are significantly more in the right panels and the puncta also appear bigger in CG8093 knockdown. 15–20 guts are examined for each genotype in one experiment, n = 3.
(TIF)

Figure S8 **Quantification of cardiac function parameters in RNAi knockdown of CG8093 in wild type and** *dcerk^1^* **.** Knockdown of CG8093 does not show significant changes in most of the parameters tested in both backgrounds. However, heart period is increased due to expansion of diastolic interval in *dcerk^1^* when CG8093 is knocked down. n = 11–13 for each group. Bar represents standard error of mean.
(TIF)

Figure S9 **Assessment of starvation sensitivity and cardiac function parameters in RNAi knockdown of Pglym in wild type and** *dcerk^1^* **.** Ubiquitous knockdown of Pglym results in significant increase in starvation sensitivity in both backgrounds. Knockdown of Pglym decreases diastolic and systolic diameters in both backgrounds. n = 11–13. Bar represents standard error of mean.
(TIF)

Table S1 **List of increased and decreased genes from microarray analysis.** List of genes whose expression either increase by 2 fold or more or decrease by 0.5 fold or less in *dcerk* ^1^ compared to *w^1118^* flies is shown. The columns represent Affymetrix ID, CG number, log fold change, P value, normalized log 2 transformed intensity and present call.
(PDF)

Table S2 **List of candidate genes from microarray analysis that contain FOXO binding sites.** The table shows genes from the microarray analysis for which potential FOXO binding sites were identified. The columns show FBgn ID with chromosome location, strand, gene start and end positions, CG numbers, peak start and end positions, distance to peak, shortest distance to peak and whether it is overlapping or nearest.
(PDF)

Table S3 **Fold change (** *dcerk^1^/w^1118^* **) of metabolites and their classification into different pathways.** The table shows relative changes in different metabolites between control and mutant flies. The metabolites have been classified into functional pathways. A p value of 0.05 or less and q value (false discovery rate) of 0.1 or less is considered statistically significant.
(PDF)

Text S1 **Methodology detailed in Supplemental Materials and Methods.** Additional details are provided for microarray analysis, metabolic assays including measurements of TAG, trehalose, ATP, free fatty acid and glycerol, QPCR analysis, immunofluorescence microscopy, activity measurements for glycolytic enzymes, assays for mitochondrial oxidative phosphorylation complexes and feeding of *Pseudomonas entomophila*.
(DOCX)
