# Supplementary figures and images for: Survival Response to Increased Ceramide Involves Metabolic Adaptation through Novel Regulators of Glycolysis and Lipolysis
Source: PLoS Genet. 2013 Jun 20;9(6):e1003556. doi: 10.1371/journal.pgen.1003556 (PMC3688504; doi:10.1371/journal.pgen.1003556)

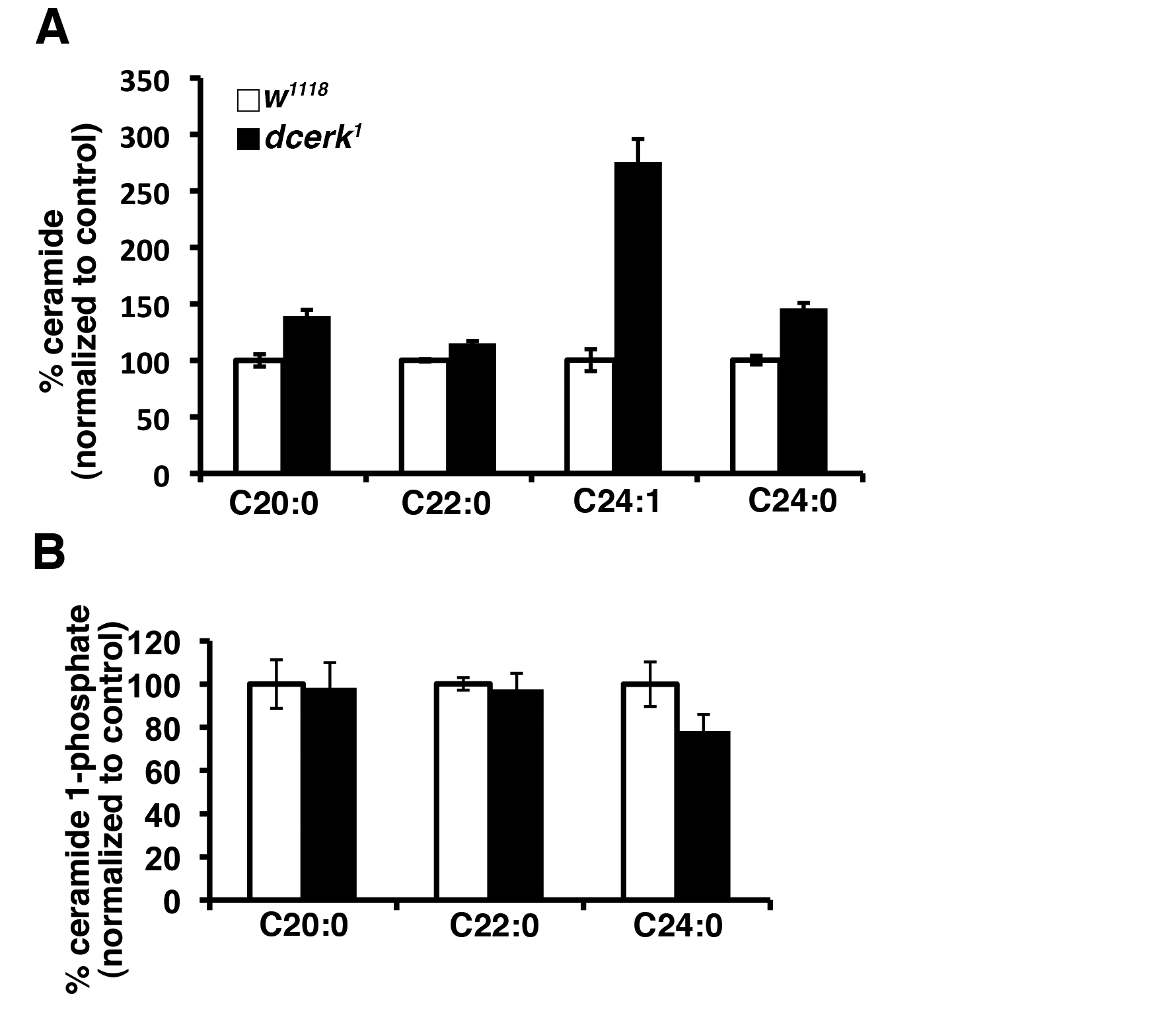

Supplement: Figure S1 — Ceramide levels increase while ceramide 1-phosphate levels are not significantly affected in dcerk1 mutant fly extracts compared to w1118 . (A). d14 long chain base ceramide with different fatty acids, C20:0, C22:0, C24;1 and C24:0 are estimated by mass spectrometry in sphingolipid enriched fractions prepared from w1118 and dcerk1 flies. The amount of ceramide is calculated based on total carbon content and then normalized to w1118. All ceramides show significant increase (P< = 0.001-0.0001) in mutant compared to w1118. n = 3, error bars represent standard deviation. (B). d14 long chain base ceramide 1-phosphate (C1P) with fatty acids C20:0, C22:0 and C24:0 are estimated by mass spectrometry. A 20% decrease in C24:0 C1P is observed in mutant relative to w1118. n = 3, error bars represent standard deviation. (TIF) [file pgen.1003556.s001.tif]

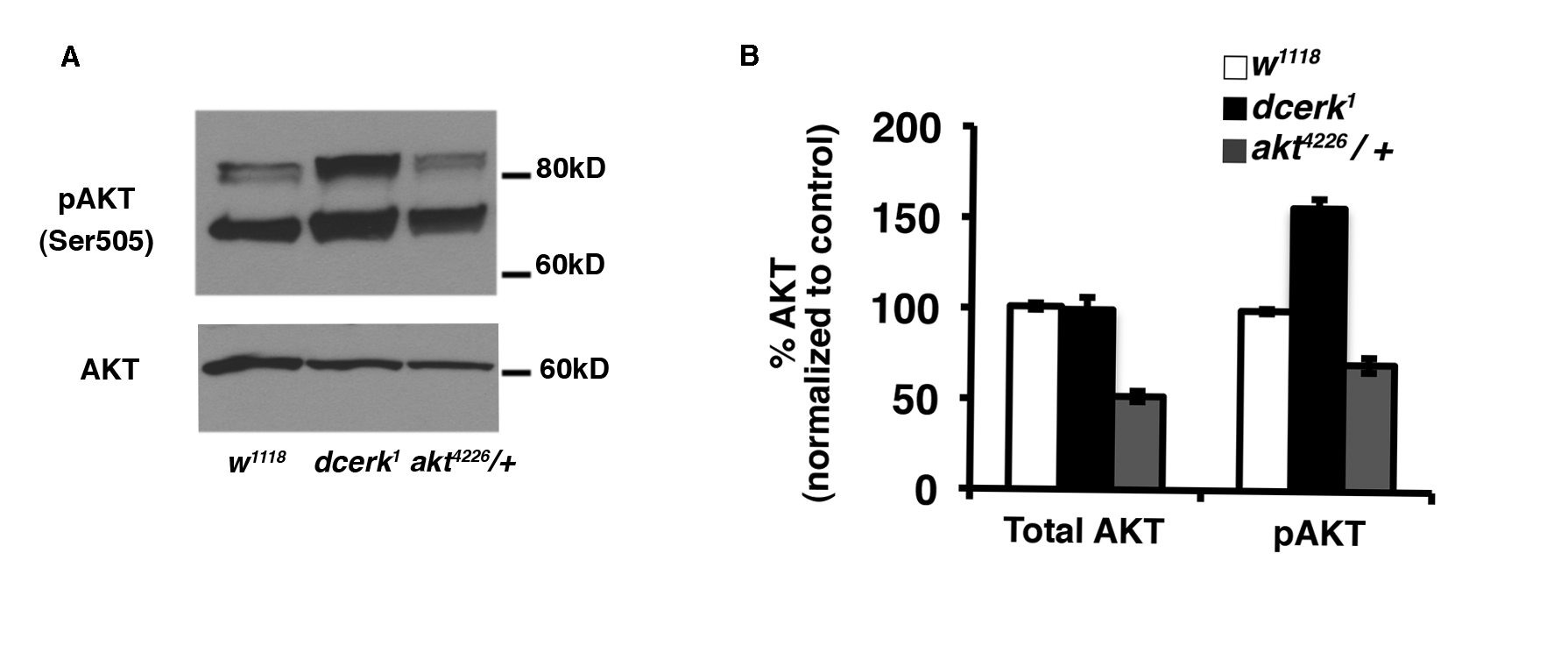

Supplement: Figure S2 — Western blot of total AKT and phospho AKT level in akt4226 /+ heterozygotes. (A). Western blot of total AKT and phospho AKT in w1118, dcerk1 and akt4226/+. (B). Quantification of the blots by densitometric scanning shows a 50% decrease in total AKT and 30% reduction in pAKT in the heterozygote. n = 3. (TIF) [file pgen.1003556.s002.tif]

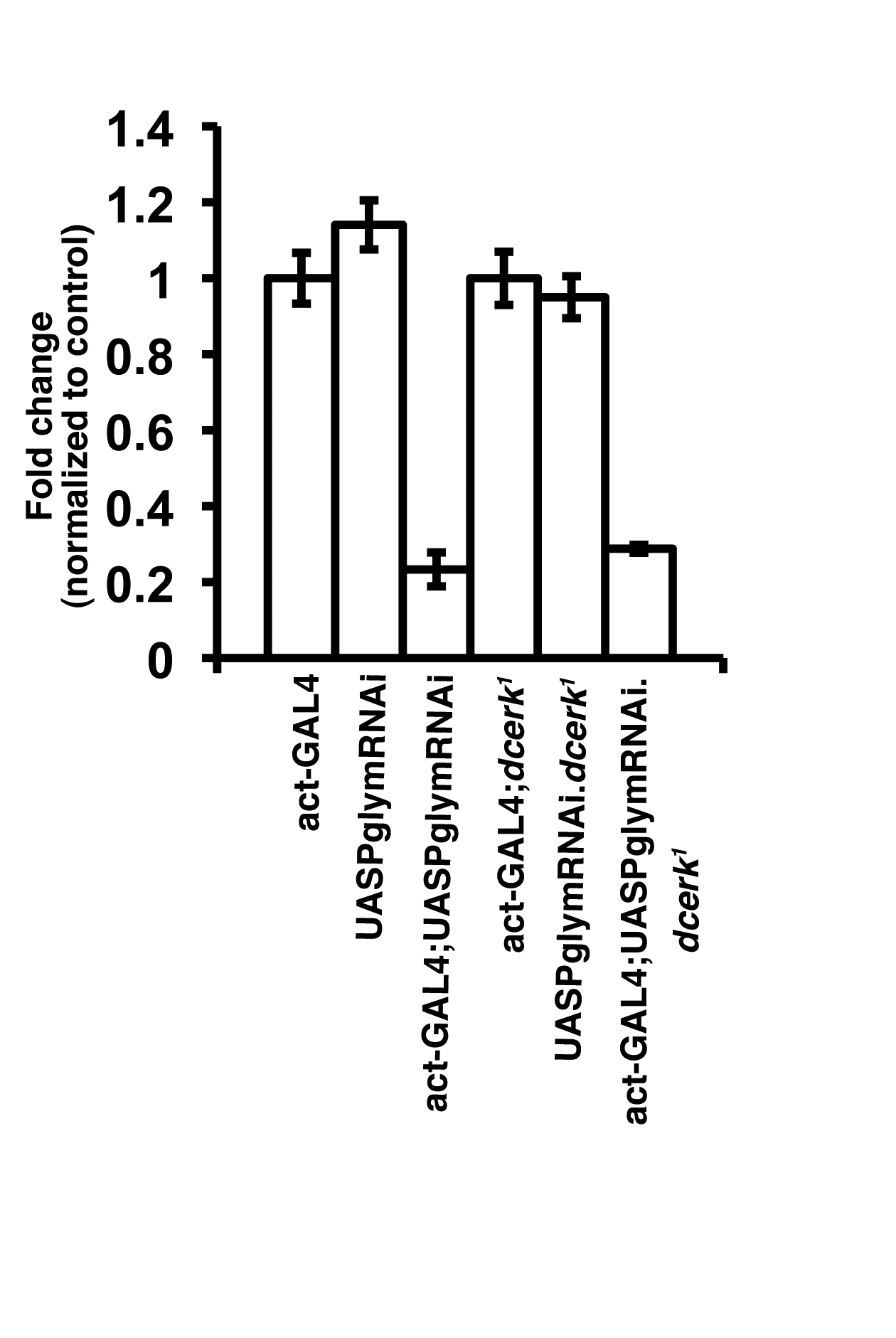

Supplement: Figure S3 — Ubiquitous RNAi knockdown of Pglym in w1118 and dcerk1 . Combining actin GAL4 driver with UASPglymRNAi transgene results in significant reduction in Pglym transcript level in w1118 and dcerk1. n = 3, error bars represent standard deviation. (TIF) [file pgen.1003556.s003.tif]

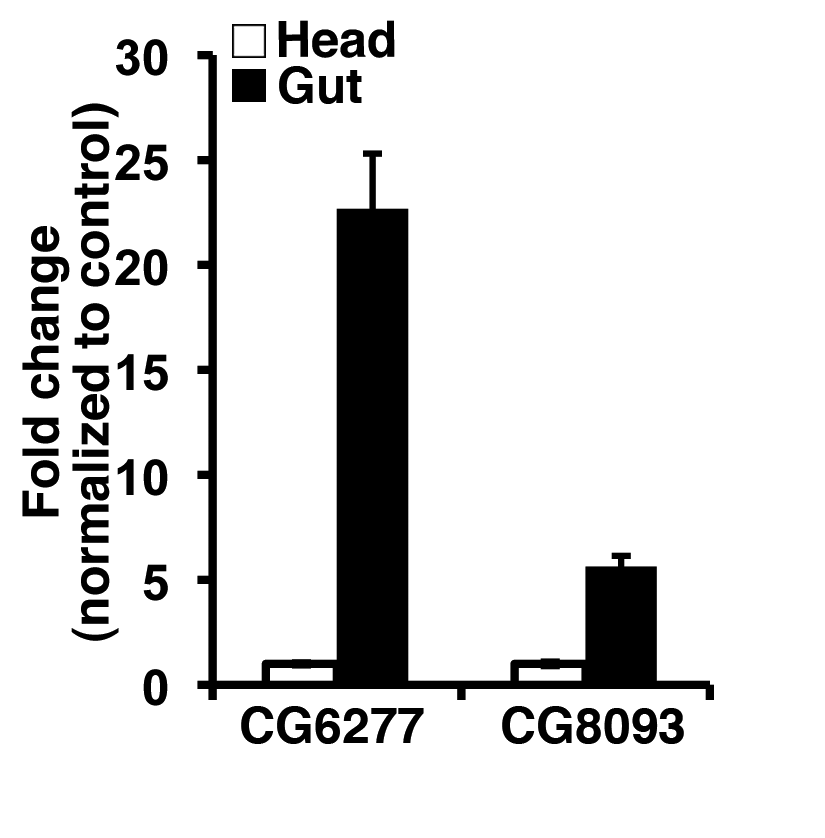

Supplement: Figure S4 — CG6277 and CG8093 show high expression in the adult midgut. QPCR analysis is carried out using total RNA extracted from adult head and dissected adult midgut from w1118. CG6277 shows approximately 20-fold increase in transcript level in the midgut compared to head while CG8093 shows a 5 fold increase suggesting these genes are highly expressed in the adult midgut. n = 3, error bars represent standard deviation. (TIF) [file pgen.1003556.s004.tif]

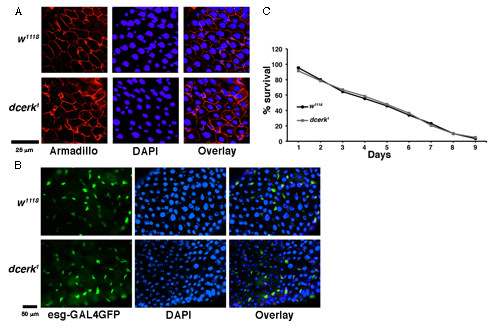

Supplement: Figure S5 — Gut epithelial integrity and epithelial renewal is not compromised in dcerk1 mutants. A. w1118 and dcerk1 gut are stained with Armadillo (β-Catenin homolog), an important component of adherens junctions. The nuclei are stained with DAPI. Armadillo staining is not significantly different between w1118 and dcerk1 suggesting epithelial integrity is not compromised in the mutant. Scale bar represents 25 µm. B. This panel shows w1118 and dcerk1 midguts expressing esg-GAL4 GFP, which marks enteroblasts and intestinal stem cells involved in epithelium renewal. GFP staining is not significantly different between control and mutant guts. The guts are also stained with DAPI. Scale bar represents 50 µm. C. Survival of dcerk1 is not significantly different from w1118 upon ingestion of Pseudomonas entomophila. Increased epithelial renewal is required in the gut to make up for damage caused by oral pathogens and flies with low epithelial renewal succumb to bacterial infection earlier than control flies. However, dcerk1 flies died at a similar rate as controls when fed Pseudomonas entomophila suggesting epithelial renewal in response to gut damage in dcerk1 is not compromised. (TIF) [file pgen.1003556.s005.tif]

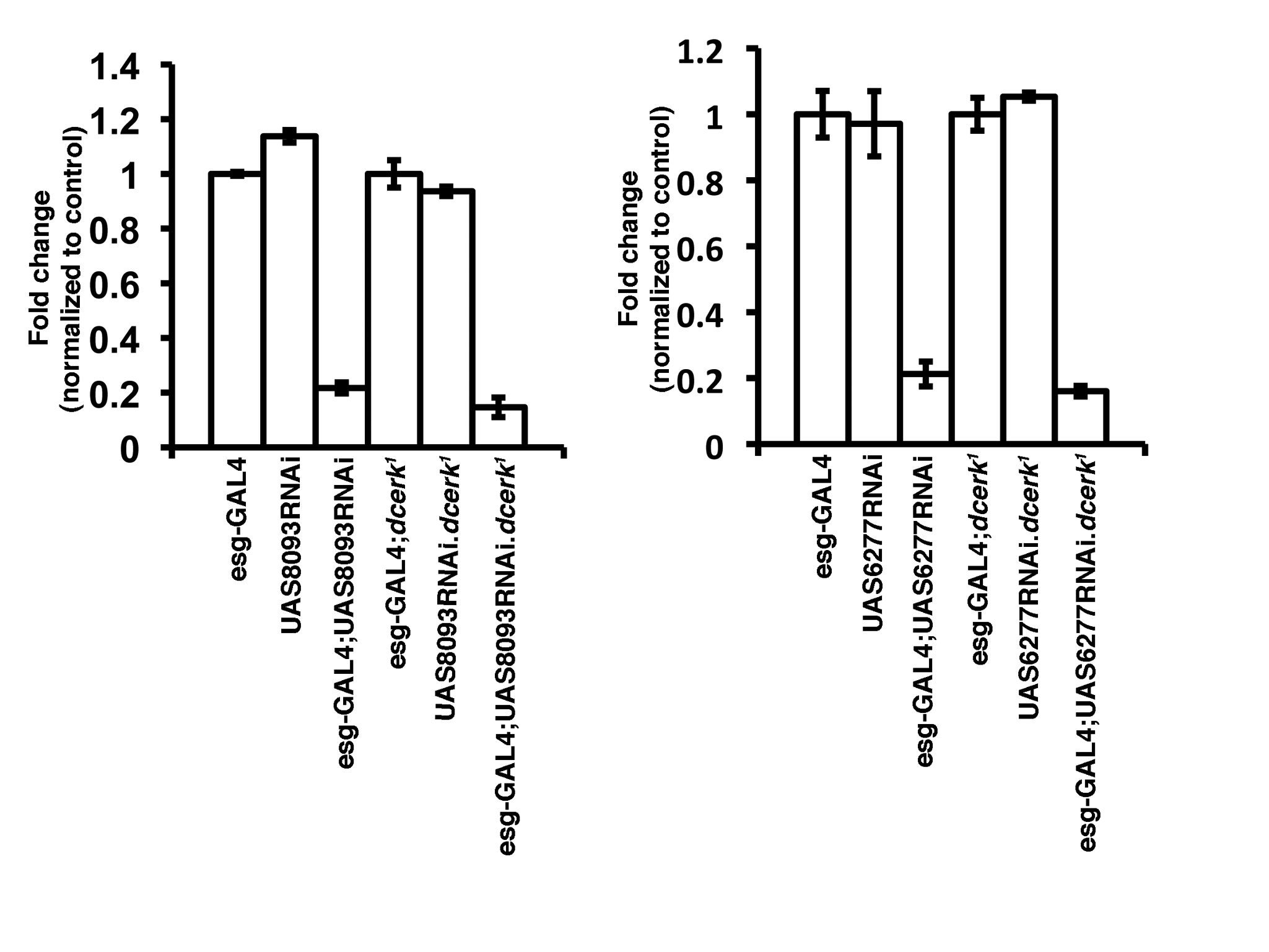

Supplement: Figure S6 — Gut specific knockdown of CG8093 and CG6277 in w1118 and dcerk1 . Combining expression of esg-GAL4 driver and UASCG8093RNAi transgene effectively reduces CG8093 transcript level in w1118 and dcerk1 flies. Similarly, combining expression of esg-GAL4 driver and UASCG6277RNAi transgene effectively reduces CG6277 transcript level in w1118 and dcerk1 flies. n = 3, error bars represent standard deviation. (TIF) [file pgen.1003556.s006.tif]

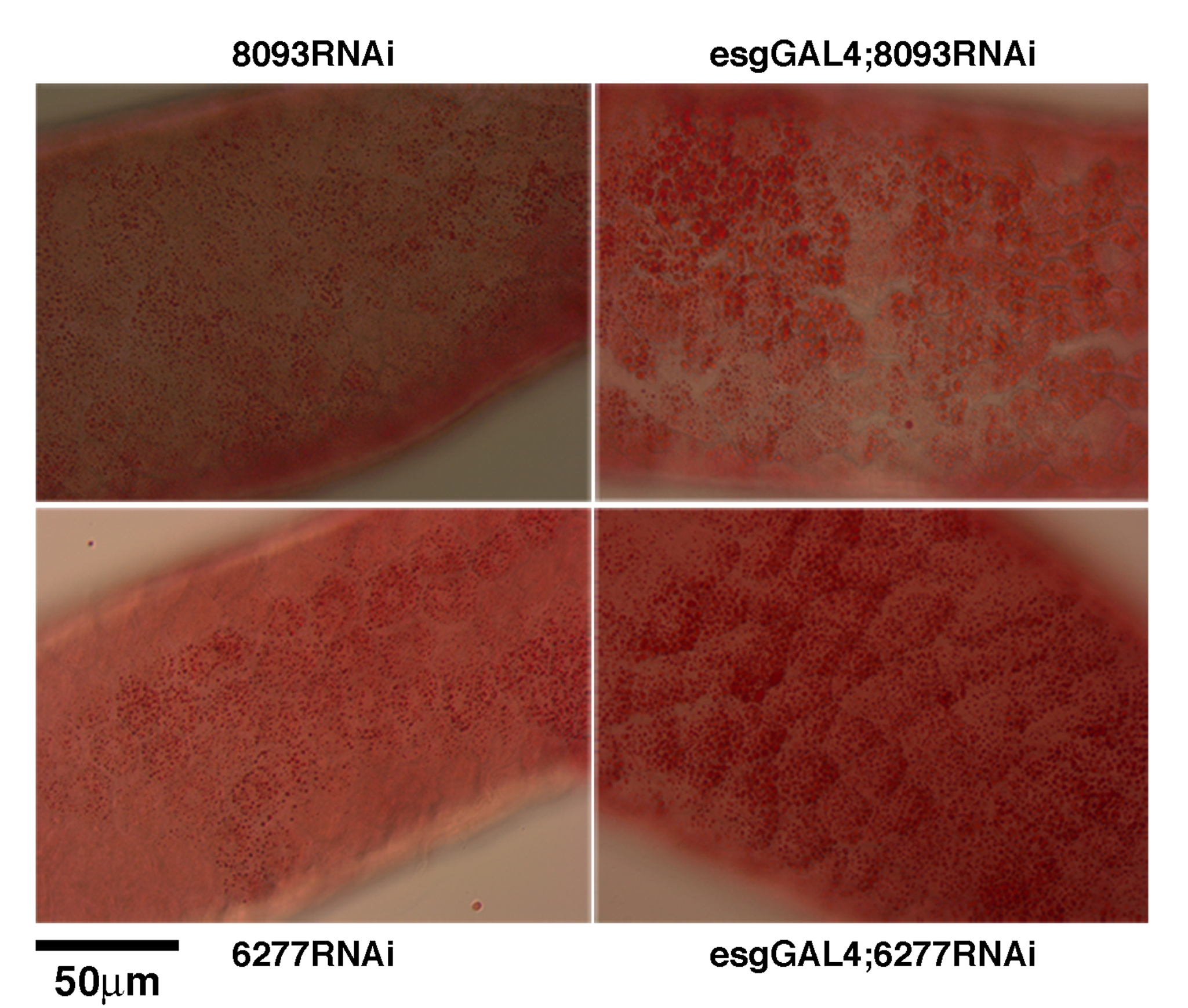

Supplement: Figure S7 — Knockdown of CG8093 and CG6277 in w1118 results in increased Oil Red O puncta in the midgut. Dissected adult midguts stained with Oil Red O show significantly increased staining in RNAi knockdown in w1118 compared to RNAi controls. Oil Red O puncta are significantly more in the right panels and the puncta also appear bigger in CG8093 knockdown. 15–20 guts are examined for each genotype in one experiment, n = 3. (TIF) [file pgen.1003556.s007.tif]

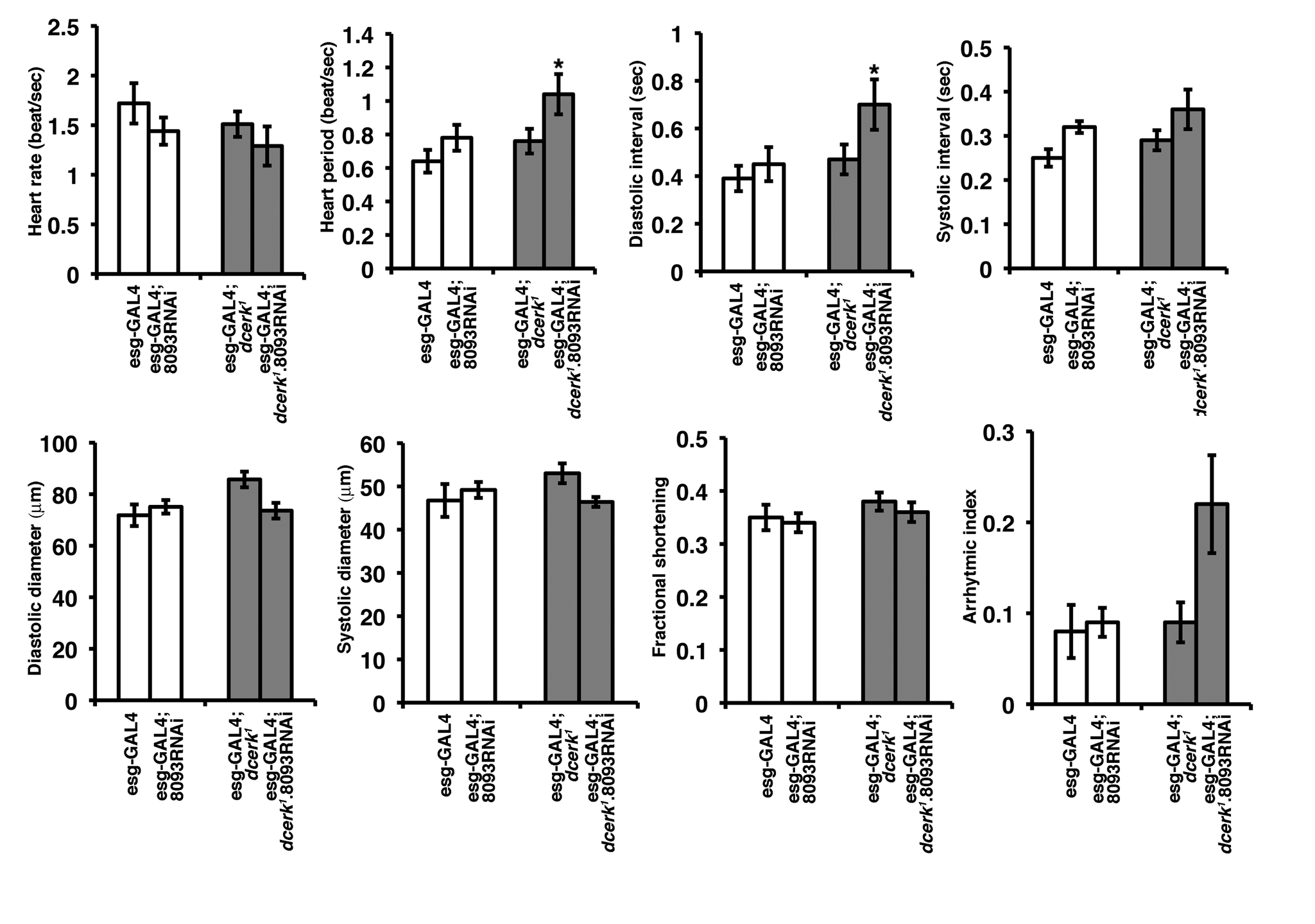

Supplement: Figure S8 — Quantification of cardiac function parameters in RNAi knockdown of CG8093 in wild type and dcerk1 . Knockdown of CG8093 does not show significant changes in most of the parameters tested in both backgrounds. However, heart period is increased due to expansion of diastolic interval in dcerk1 when CG8093 is knocked down. n = 11–13 for each group. Bar represents standard error of mean. (TIF) [file pgen.1003556.s008.tif]

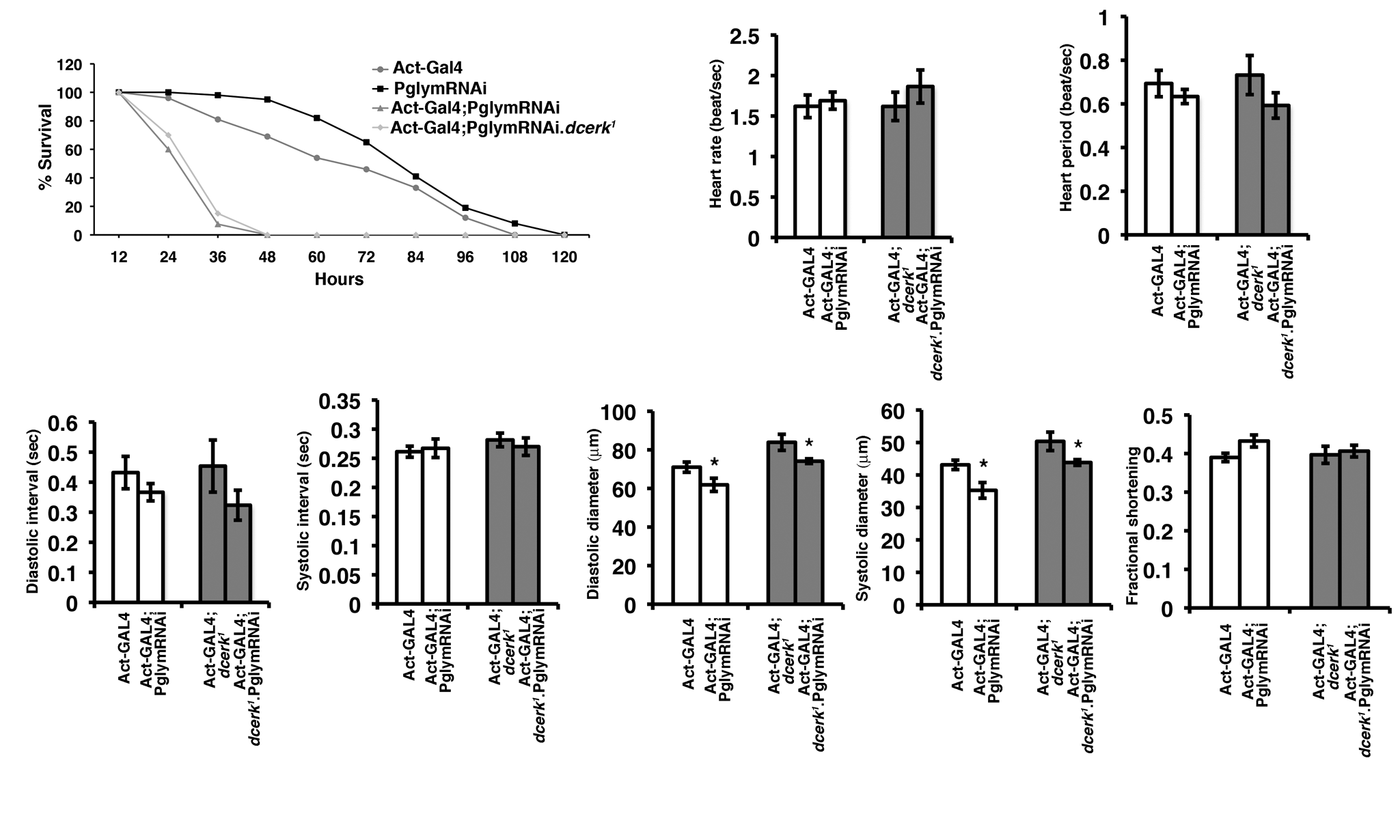

Supplement: Figure S9 — Assessment of starvation sensitivity and cardiac function parameters in RNAi knockdown of Pglym in wild type and dcerk1 . Ubiquitous knockdown of Pglym results in significant increase in starvation sensitivity in both backgrounds. Knockdown of Pglym decreases diastolic and systolic diameters in both backgrounds. n = 11–13. Bar represents standard error of mean. (TIF) [file pgen.1003556.s009.tif]
