# Supplementary material for: Survival Response to Increased Ceramide Involves Metabolic Adaptation through Novel Regulators of Glycolysis and Lipolysis
Source: PLoS Genet. 2013 Jun 20;9(6):e1003556. doi: 10.1371/journal.pgen.1003556 (PMC3688504; doi:10.1371/journal.pgen.1003556)
Supplement: Table S1 — List of increased and decreased genes from microarray analysis. List of genes whose expression either increase by 2 fold or more or decrease by 0.5 fold or less in dcerk 1 compared to w1118 flies is shown. The columns represent Affymetrix ID, CG number, log fold change, P value, normalized log 2 transformed intensity and present call. (PDF) [file pgen.1003556.s010.pdf]

INCREASED GENES

ID	Annotation ID	logFC	P.Value	adj.P.Value	Normalized log2 transformed intensity						Present Call					
					X2W1118.A	X2W1118.B	X2W1118.C	CK163.D	CK163.E	CK163.F	2W1118-A	2W1118-B	2W1118-C	CK163-D	CK163-E	CK163-F
1623027_s_at	CG6277	2.959166147	2.70E-07	0.000121102	6.342676672	6.597616121	6.938717895	9.475316474	9.663041646	9.618151011	P	P	P	P	P	P
1623109_at	CG4593	1.879859455	2.00E-05	0.002359057	6.746532893	6.390288784	6.91159318	8.825080329	8.25256337	8.610349522	A	M	A	P	P	P
1623179_at	CG15415	1.113543899	0.001516842	0.039697096	7.184653372	6.719814173	6.882137961	8.447002696	7.612246366	8.06798814	P	P	P	P	P	P
1623296_at	CG32625	2.159222734	0.000170982	0.009502767	7.326325141	6.657830318	7.060635649	9.779940755	8.651159593	9.091358961	P	P	P	P	P	P
1623398_at	CG4830	2.24893279	2.42E-05	0.002642525	5.123362932	5.024681029	5.806601698	7.453438444	7.861573032	7.386432552	A	A	A	P	P	P
1623486_at	CG7900	2.815149144	8.50E-09	1.55E-05	5.597499541	5.650449126	5.491494196	8.284967966	8.471890676	8.428031654	M	P	A	P	P	P
1623521_at	CG11909	5.935632832	9.93E-09	1.57E-05	4.411348521	4.567716442	4.189699712	9.916919427	10.60417658	10.45456716	A	A	A	P	P	P
1623601_at	CG8221	1.407805069	2.45E-05	0.002642525	5.265567158	5.48985005	5.423135943	6.504977265	6.891428572	7.00556252	P	P	P	P	P	P
1623689_a_at	CG14642	1.417316836	0.000666548	0.023480343	6.35981525	6.907862689	6.458761825	7.522034023	8.414344257	8.042011991	P	P	P	P	P	P
1623789_at	CG31776	1.16346141	0.000168532	0.009449771	4.464969391	4.489089141	4.542270079	5.31388432	5.96218471	5.710643813	A	A	A	M	P	P
1624092_at	CG31039	1.36642372	1.83E-05	0.002245684	10.70791616	11.08587506	10.59990135	12.20351921	12.11325563	12.17618889	P	P	P	P	P	P
1624269_at	CG8825	1.88875888	0.000277303	0.012944444	5.36427518	4.660004283	5.170654363	7.359874501	6.387225625	7.114110342	P	A	P	P	P	P
1624332_s_at	CG18217	1.137631994	5.27E-05	0.004327818	7.271340397	7.092287421	7.19249308	8.547549074	8.07832969	8.343138118	P	P	P	P	P	P
1624817_at	CG3212	1.322955112	0.000145151	0.008644504	5.241388766	4.790030238	4.780836691	6.478509097	5.965626378	6.336985554	A	A	A	P	M	P
1624914_at	CG8690	1.393153473	0.001445856	0.038326774	7.713618457	8.653380057	7.959096789	9.21199345	9.906499756	9.387062515	P	P	P	P	P	P
1625042_at	CG31288	1.912736107	2.39E-06	0.0005758	8.609833149	8.766817999	8.582392437	10.27535883	10.69774056	10.72415252	P	P	P	P	P	P
1625153_at	CG33105	2.302904437	2.36E-06	0.0005758	5.189181938	5.248716202	5.685304739	7.469315215	7.887331082	7.675269893	A	A	A	P	P	P
1625366_at	CG4125	1.146958909	0.000127153	0.007889929	7.841430975	7.599595966	7.858777827	9.026130281	8.606285251	9.108265962	P	P	P	P	P	P
1625382_at	CG1151	1.857103594	2.96E-07	0.000123885	4.492953661	4.257314494	4.558452082	6.293024813	6.289138297	6.297867908	A	A	A	P	P	P
1625727_at	CG32006	1.496767782	0.00054672	0.02086273	5.390477437	5.548193342	5.29859069	6.380035473	7.445243079	6.902286264	P	P	P	P	P	P
1625860_s_at	CG32939	1.286479181	1.23E-05	0.001800345	7.905086489	7.799921315	8.125332319	9.381999488	9.102511952	9.205266226	P	P	P	P	P	P
1625965_a_at	CG1148	1.264845476	0.000149843	0.008803017	5.055869158	4.460438794	4.876654823	5.915254709	6.004612379	6.267632116	A	A	A	A	P	A
1626138_s_at	CG10579	1.304423563	9.42E-05	0.006288919	8.695602852	8.2991989	8.468145966	9.478998278	9.985376014	9.911844115	P	P	P	P	P	P
1626164_at	CG31380	1.683574394	2.10E-05	0.002407133	5.641130207	6.232063333	5.715155728	7.394837789	7.634687051	7.609547611	A	A	A	P	P	P
1626248_at	CG18547	1.07840115	6.09E-05	0.004689685	7.859865721	8.120937158	7.700026181	8.846878891	9.069079221	9.000074399	P	P	P	P	P	P
1626324_at	Cyp309a1	1.116709018	0.001624948	0.041336931	6.821870946	7.643272308	6.869474604	8.046269964	8.292341461	8.346133486	P	P	P	P	P	P
1626394_at	CG18030	1.941231581	0.000458938	0.018694095	8.275184503	7.811329534	8.827658184	10.69055034	10.31181619	9.735500435	P	P	P	P	P	P
1626405_at	CG8357	1.101793815	9.58E-05	0.006329419	5.809657964	5.789730464	6.130135839	6.786345478	7.144333037	7.104227196	P	P	P	P	P	P
1626435_at	CG15263	2.030410592	0.000242439	0.01184202	5.034723437	5.356905132	5.298078955	6.803115738	7.98478861	6.993034951	A	P	A	P	P	P
1626742_at	CG8964	1.079207013	0.000863258	0.0275549	6.191117348	5.713287359	5.787035842	7.206760977	6.604257747	7.118042865	P	M	P	P	P	P
1626882_at	CG8807	1.368692949	8.39E-06	0.001328147	6.380439349	6.492161825	6.259247195	7.73015444	7.939270727	7.568502047	P	P	P	P	P	P
1626908_at	CG8066	1.424827952	0.00017018	0.009486019	5.085509669	4.651100249	4.927866847	6.518722648	6.519407696	5.900830275	P	M	A	P	P	P
1627119_at	CG8329	1.39330624	1.18E-05	0.001735154	8.644124824	8.497191458	8.575254088	9.84417762	10.23245016	9.819861308	P	P	P	P	P	P
1627162_at	CG31681	1.516094181	0.000773553	0.025856043	9.105998831	8.359657449	8.785521918	10.66957315	9.769085111	10.36080248	P	P	P	P	P	P
1627169_at	CG3264	2.257265586	0.000651282	0.023332882	5.91528653	4.763429845	6.005781851	8.419226186	7.427822521	7.609246278	P	A	P	P	P	P
1627499_at	CG2016	1.108234821	4.30E-06	0.000839949	7.981472602	7.749270468	7.870588562	8.984216774	8.945750718	8.996068602	P	P	P	P	P	P
1627633_at	CG8932	1.18857068	6.91E-06	0.00115953	6.904310123	6.990755907	6.72730907	7.957565338	8.089550015	8.140971788	P	P	P	P	P	P
1627669_at	CG1791	1.770590536	4.62E-07	0.000176219	6.106535245	5.962206176	5.890384563	7.698939	7.896544996	7.675413596	P	P	P	P	P	P
1627736_at	CG11062	1.302420119	0.000232297	0.01152483	4.647962539	4.408264924	4.490354065	5.456938363	6.21712341	5.779780113	P	P	A	P	P	P
1627869_at	character(0)	1.51079125	7.99E-07	0.000260441	10.04780085	10.1072847	9.854907985	11.5600649	11.50920575	11.47309664	P	P	P	P	P	P
1627972_at	CG9822	1.348857627	2.94E-05	0.002984522	4.496938576	4.69723884	4.233609918	5.898763993	5.931895454	5.643700768	A	A	A	P	P	P
1628806_at	CG5279	2.152122354	3.87E-07	0.000152998	6.539212465	6.60435732	6.297582752	8.469780201	8.757250883	8.670488514	A	A	A	P	P	P
1628918_at	character(0)	1.612420886	0.000658277	0.023426692	5.169374477	4.474718375	4.505513703	6.868913858	6.000717613	6.11723774	P	P	A	P	P	P
1629242_x_at	character(0)	1.855179353	8.25E-07	0.000260441	7.378233317	7.14699654	6.993821439	8.971651374	9.004694937	9.108243044	P	P	M	P	P	P
1629362_at	CG32475	2.041153275	1.34E-05	0.001895088	4.687422035	4.877440209	4.107680691	6.555061434	6.604440205	6.636501121	A	A	A	P	P	P
1629718_at	CG16727	1.597613636	4.57E-05	0.003918992	7.180317949	7.429845855	7.094194308	8.54779548	9.200323077	8.749080464	P	P	P	P	P	P
1629955_at	CG13309	1.999005054	2.22E-07	0.000108057	7.941152655	8.037759794	7.854010957	10.10327819	9.835447571	9.891212811	P	P	P	P	P	P
1630065_at	CG6912	2.229956323	1.20E-07	8.41E-05	6.44356419	6.440264672	6.526107926	8.519997935	8.727624317	8.852183504	P	P	P	P	P	P
1630109_at	CG8628	2.142967566	1.29E-07	8.74E-05	8.837176436	8.772496294	8.644316802	11.02291777	10.90316835	10.7568061	P	P	P	P	P	P
1630244_s_at	CG31809	1.69709993	4.65E-07	0.000176219	5.759034871	5.641414689	5.844459686	7.499189124	7.519353765	7.317666146	P	P	P	P	P	P
1630255_at	CG31676	1.022603732	1.55E-05	0.002050097	8.190308821	8.051763777	8.149239771	9.064175414	9.311653077	9.083295073	P	P	P	P	P	P
1630359_at	CG31810	2.20605863	3.86E-06	0.000804164	4.500707338	5.114913121	4.683362906	6.921142058	7.154172399	6.841844797	A	A	A	P	P	P
1630555_at	CG33346	4.75462026	1.19E-08	1.74E-05	5.966599089	6.159419726	5.650262136	10.45365549	10.8024956	10.78399065	P	P	P	P	P	P
1630598_at	CG18179	2.357241505	3.33E-05	0.003222748	6.496612108	6.501759681	6.05970285	8.148835447	8.923060892	9.057902814	P	A	A	P	P	P
1630645_at	CG3528	1.918090452	0.000153769	0.008924426	5.176927551	5.432851343	5.501191689	6.73890026	8.190870635	7.295471042	P	A	P	P	P	P
1630660_at	CG14033	1.230339253	0.001587761	0.040884855	6.186066478	5.97539226	6.223350564	7.84976449	6.807953566	7.418109003	P	P	P	P	P	P
1630740_at	CG4363	1.458691836	0.000454402	0.018680761	8.222762752	9.191216343	8.550108393	9.966754174	10.26448771	10.10892111	P	P	P	P	P	P
1630842_s_at	CG32640	1.418817326	4.62E-06	0.000881182	9.160499389	9.037463864	9.189647881	10.71026758	10.58707456	10.34672097	P	P	P	P	P	P
1630886_at	CG6584	1.044786766	5.62E-06	0.001034847	8.522079582	8.526460327	8.594280203	9.688361521	9.479800237	9.609018652	P	P	P	P	P	P
1630957_s_at	CG11128	1.505832235	3.36E-06	0.000715581	8.736543109	8.572277773	8.5889176	10.30240637	9.938096009	10.17473281	P	P	P	P	P	P
1631127_at	CG10424	1.215446737	3.17E-06	0.000701433	7.226908084	7.038096267	7.070939717	8.429835359	8.272148017	8.280300903	P	P	P	P	P	P
1631165_at	CG4688	1.100364847	4.09E-05	0.003620352	5.680850437	5.70128969	5.49739868	6.588958791	6.639046162	6.952628395	A	A	A	P	P	P
1631349_s_at	character(0)	2.000930168	1.07E-06	0.000301534	5.642996904	5.252255274	5.561323367	7.352454991	7.491743484	7.615167576	P	P	P	P	P	P
1631529_at	CG17240	4.5638879	1.12E-07	8.18E-05	4.55439754	4.768941453	4.51114868	9.59052386	8.732347669	9.203279844	A	A	A	P	P	P
1631805_at	CG3397	3.227317707	2.51E-09	6.80E-06	4.334096246	4.395637301	4.496102608	7.688395402	7.544544681	7.674849193	A	A	A	P	P	P
1631962_at	CG33192	1.256303056	5.84E-06	0.001064989	7.298474954	6.988861436	7.050385174	8.433693197	8.321433176	8.35150436	P	P	P	P	P	P

1632001_at	CG7102	1.226809913	7.75E-05	0.005539949	4.553783146	4.654566717	4.66229659	5.965395906	5.514658194	6.071022091	A	A	A	P	P	P
1632003_a_at	character(0)	1.143571123	1.93E-05	0.002298045	7.426893879	7.107633991	7.41082039	8.541427767	8.36154409	8.473089774	P	P	P	P	P	P
1632120_at	CG15533	1.28334589	1.86E-05	0.002245684	7.672641412	7.787316793	7.455007041	8.844149085	8.80635592	9.11449791	P	P	P	P	P	P
1632624_at	CG11330	1.7253972	0.000198339	0.010355154	7.370757858	6.87504589	7.218754581	9.303261097	8.37614145	8.961347383	P	P	P	P	P	P
1633009_a_at	CG6640	1.44943204	8.14E-05	0.00569423	7.446229593	7.417336248	6.99328551	8.443663282	8.987940545	8.773543644	P	P	P	P	P	P
1633048_at	CG8193	1.028949791	0.000544536	0.02086273	7.223224255	6.947743797	7.293099019	8.512949038	8.134975032	7.902992375	P	P	P	P	P	P
1633147_at	CG10659	1.338968791	8.02E-05	0.00569423	6.381309117	6.433704803	6.31232418	7.812620358	7.988025798	7.343598317	P	P	P	P	P	P
1633167_s_at	CG32496	3.604430264	6.59E-06	0.00112443	6.839022405	6.551488059	6.111456991	9.515251243	10.62864646	10.17136054	P	P	P	P	P	P
1633208_at	character(0)	1.331710034	7.09E-05	0.00520307	6.016830305	6.390396127	5.746437579	7.398897336	7.431617842	7.318278935	P	A	A	P	P	P
1633214_at	CG4835	1.068469523	0.000435355	0.018213787	4.676959565	5.190754673	4.938803505	5.797261506	5.933570839	6.281093966	A	A	A	A	P	P
1633304_at	CG1967	1.204671947	4.32E-05	0.003750719	8.663847715	8.309336589	8.773368967	9.891725986	9.699970891	9.768872234	P	P	P	P	P	P
1633471_at	CG11765	3.673887364	4.12E-06	0.000822723	7.040452362	6.92999387	7.742852179	10.39312901	11.24741471	11.09441679	P	P	P	P	P	P
1633540_at	CG8147	1.37387587	0.001417395	0.038156917	8.505093219	7.619945689	8.655328789	9.418356295	9.823459986	9.660179027	P	P	P	P	P	P
1633545_at	CG7496	1.580084421	3.21E-05	0.003184376	7.103733403	7.758199523	7.548365589	8.951291122	9.049738606	9.14952205	P	P	P	P	P	P
1633639_at	CG10833	1.325571441	9.65E-05	0.006329419	9.253208991	9.737859789	9.264844068	10.50783818	10.90380282	10.82098618	P	P	P	P	P	P
1633727_s_at	CG8789	1.450693072	3.17E-06	0.000701433	7.117608341	6.767088616	7.057496872	8.472123731	8.416644171	8.405505142	P	P	P	P	P	P
1633880_s_at	CG8533	2.126610828	2.48E-05	0.002657923	4.990268899	5.133843334	4.69969514	7.463920003	6.637754503	7.101965352	A	A	A	P	P	P
1634097_s_at	CG6300	1.372743918	4.10E-06	0.000822723	6.119794414	6.106831568	6.423176959	7.559149805	7.644574432	7.564310458	P	P	P	P	P	P
1634125_at	CG30440	1.134562747	0.00039596	0.016901437	7.478203854	7.068582588	7.238784072	8.702909957	8.070937167	8.415411632	P	P	P	P	P	P
1634152_at	CG12242	1.57396792	0.00019037	0.010191796	5.644062993	5.908115823	5.15456732	6.962710231	7.474761461	6.991178206	P	P	P	P	P	P
1634468_at	CG13397	1.009293286	0.001351964	0.036813821	7.750504465	7.598436449	7.56997595	8.926987616	8.179803173	8.840005932	P	P	P	P	P	P
1634514_at	CG33258	1.027042303	0.000237891	0.011680063	9.768414289	10.21495257	9.887734182	10.77000528	11.14855223	11.03367044	P	P	P	P	P	P
1634515_at	CG8093	1.168795198	0.001826358	0.044719805	8.527391696	9.460473959	8.61121584	9.988757292	9.971436944	10.14527285	P	P	P	P	P	P
1634591_at	CG11878	1.239891464	9.18E-05	0.006195013	8.797247767	9.080838399	8.61540627	9.827118826	10.17529246	10.21075554	P	P	P	P	P	P
1634633_s_at	character(0)	5.343108161	1.94E-10	3.27E-06	6.232515027	6.033482776	5.939397228	11.3577907	11.45629439	11.42063442	A	A	A	P	P	P
1634786_at	CG7106	1.329145363	0.000526267	0.020564548	7.368847701	8.069374262	7.921695645	9.036226734	9.449846183	8.861280779	P	P	P	P	P	P
1634815_at	CG31104	1.938325402	1.92E-05	0.002298045	7.584158671	8.236427497	7.842723765	9.600190069	10.07091581	9.807180256	P	P	P	P	P	P
1634903_at	CG9981	1.710951115	3.34E-06	0.000715581	5.018412441	4.839749904	5.074482331	6.85108514	6.44303513	6.77137775	P	P	P	P	P	P
1634991_at	CG8083	1.468739306	1.25E-06	0.000347983	7.907003238	8.088642659	7.896621739	9.311715281	9.483130316	9.503639959	P	P	P	P	P	P
1635306_at	CG4650	3.13122076	8.15E-07	0.000260441	4.136377545	3.963678917	4.515505422	7.237978824	7.10260071	7.668644631	A	A	A	P	P	P
1635343_a_at	CG3217	1.097848745	5.41E-05	0.004404509	7.559871835	7.356096876	7.631104961	8.8253768	8.444352809	8.570890299	P	P	P	P	P	P
1635725_a_at	CG31352	1.190485302	1.30E-05	0.001865182	8.747206034	8.865272175	8.890341243	9.83338689	10.18945419	10.05143428	P	P	P	P	P	P
1635770_at	CG31097	1.904109631	1.86E-07	0.00010363	4.98955717	5.018347142	4.761307058	6.847033325	6.788025312	6.846481626	A	A	A	P	P	P
1635936_at	CG13822	1.885672776	5.91E-06	0.001065853	7.272138607	6.798159247	7.163704446	9.185725759	8.815316014	8.889978856	P	P	P	P	P	P
1636031_at	CG1294	1.43381376	0.000623698	0.02273141	4.637857711	4.710042704	4.565009098	6.680242718	5.710820692	5.823287382	A	A	A	P	P	P
1636073_at	CG32344	1.095855026	0.000371102	0.016094103	7.694794218	7.256183303	7.576873495	8.851162454	8.306071864	8.658181776	P	P	P	P	P	P
1636194_s_at	character(0)	2.984935194	0.000561221	0.021315167	4.939551722	4.94147534	5.233905036	8.982994967	6.796077793	8.290664921	A	A	A	P	P	P
1636409_at	CG11034	1.473061649	6.31E-06	0.001097775	5.77053115	5.364133856	5.582721906	7.156905779	7.035842841	6.943823238	P	P	P	P	P	P
1636423_at	CG8012	1.440423562	6.91E-07	0.000233806	9.042716266	9.211930442	9.063289771	10.52331274	10.6261957	10.48969872	P	P	P	P	P	P
1636974_at	CG32146	1.539477464	1.37E-05	0.001900535	7.3043746	6.855668159	7.123054829	8.76074195	8.431276062	8.709511969	P	P	P	P	P	P
1637055_s_at	character(0)	2.420947468	9.67E-08	7.33E-05	7.057394521	7.025500777	6.769549754	9.247264128	9.431329003	9.436694324	A	A	A	P	P	P
1637462_at	CG13833	1.057263427	0.00026029	0.012368345	8.134683335	8.568454386	8.148825894	9.192529552	9.571764446	9.259459897	P	P	P	P	P	P
1637467_at	CG5245	1.381171241	0.001908544	0.046018733	3.971309646	4.111431229	4.314073662	6.11338544	4.894136388	5.532806434	A	A	A	P	P	P
1637481_at	CG6890	1.175494915	2.38E-05	0.002642525	7.376229128	7.131753894	7.382491671	8.554591108	8.278073454	8.584294874	P	P	P	P	P	P
1637567_at	CG30360	1.445053831	0.000209194	0.010773467	9.018570774	9.724924131	9.234995416	10.5130064	11.02157387	10.77907154	P	P	P	P	P	P
1637659_a_at	CG18146	1.10634063	1.40E-05	0.001920181	6.021460586	6.035589843	5.873446062	6.928303516	7.228582958	7.092631906	P	P	P	P	P	P
1637857_at	CG3410	3.310398521	8.36E-08	6.82E-05	3.69307044	3.860165609	3.763992291	7.248681653	6.759609141	7.240133109	A	A	A	P	P	P
1638080_at	CG13658	2.676573581	2.75E-07	0.000121102	4.420731553	4.242532925	4.433470556	6.981066561	7.32332331	6.822065906	A	A	M	P	P	P
1638182_at	CG5999	1.087658066	0.000422685	0.01784128	7.490541244	7.604617379	7.213900854	8.431167716	8.862707196	8.278158764	P	P	P	P	P	P
1638264_at	CG1304	4.111902332	4.56E-10	3.27E-06	4.173437526	4.267848338	4.319840953	8.361597928	8.452735253	8.282500631	A	A	P	P	P	P
1638393_at	character(0)	1.348286599	2.53E-05	0.002680896	5.136887509	4.85981782	4.872243954	6.548898905	6.252483753	6.11242642	P	P	P	P	P	P
1638407_at	CG31244	3.264344205	8.02E-06	0.001299345	5.235829542	4.963566002	4.815610264	7.704101759	8.869815727	8.234120936	A	A	A	P	P	P
1638428_at	character(0)	1.083470188	1.28E-05	0.001848769	6.733119648	6.703349636	6.515788944	7.637134372	7.705289096	7.860245324	M	P	M	P	P	P
1638687_at	character(0)	1.981382039	1.06E-06	0.000301534	4.346408857	3.918112877	4.01928086	6.060316538	6.170721262	5.996910911	A	A	A	P	P	P
1638812_at	CG11501	3.437839843	2.39E-05	0.002642525	5.230500635	5.153153423	6.225765855	9.440117964	8.443491941	9.039329537	A	A	P	P	P	P
1639009_at	CG10962	1.153205445	0.000810457	0.026528104	4.760248757	4.58550508	4.967556714	6.309344469	5.514163941	5.949418477	P	P	P	P	P	P
1639036_at	CG8857	1.517799407	7.55E-05	0.005420958	7.384149332	6.735918238	7.218038257	8.836907866	8.450704232	8.60389195	P	P	P	P	P	P
1639180_at	CG12505	1.366748853	3.96E-06	0.000812158	11.38409838	11.29169986	11.33746585	12.51181807	12.74385889	12.8578337	P	P	P	P	P	P
1639222_at	CG32191	1.103365735	3.48E-05	0.003294454	7.032700237	7.291537878	7.099080687	8.073989945	8.420590911	8.238835153	P	P	P	P	P	P
1639262_at	CG11669	1.858291683	0.000474009	0.018992433	7.833409666	8.974263178	8.529106005	10.02953644	10.6962279	10.18588955	P	P	P	P	P	P
1639272_at	CG3739	1.590090153	0.000321793	0.014417545	8.6946591	9.406164047	8.83368913	10.16840252	10.91047818	10.62590204	P	P	P	P	P	P
1639280_at	CG6322	1.040886263	0.000375693	0.016255106	7.750444905	7.499590136	7.764029363	9.01260521	8.408227526	8.715890458	P	P	P	P	P	P
1639323_at	CG31508	2.314126452	0.001630786	0.041429834	8.600616664	9.427295126	10.63907568	11.83439461	11.65287727	12.12209494	P	P	P	P	P	P
1639401_at	CG8696	1.420882009	0.000152291	0.008908096	10.343136	11.05450624	10.65439842	11.87935336	12.26632171	12.16901161	P	P	P	P	P	P
1639502_at	CG8942	1.457832058	0.000146916	0.008673982	6.139661208	6.22922926	5.61838341	7.718386889	7.440084342	7.202298819	P	P	P	P	P	P
1639530_at	CG14528	1.231991817	0.000677442	0.023668533	5.386991377	6.236260057	5.637598507	6.81841273	7.068665893	7.069746768	A	M	P	P	P	P
1639694_s_at	CG12505	1.455482967	1.86E-06	0.000482701	10.30106894	10.36430551	10.31977202	11.60094055	11.83529385	11.91536096	P	P	P	P	P	P
1639713_at	CG14242	1.57753506	0.000345831	0.01520692	4.437983086	4.647963859	4.155615357	5.446461427	6.231764907	6.295941148	A	A	A	P	P	P

1639896_at	character(0)	1.976307587	0.000614673	0.022488953	4.768196077	4.758474882	4.850679508	7.169286995	5.904443074	7.232543159	P	P	A	P	P	P
1639903_at	CG2071	2.308330001	2.22E-07	0.000108057	6.725051036	6.367218644	6.458443314	8.747457753	8.948665257	8.779579986	P	P	P	P	P	P
1639907_at	CG11951	1.773806019	3.99E-06	0.000812158	4.871140992	4.447182324	4.398271774	6.429406732	6.327536888	6.281069529	A	A	A	P	P	P
1640109_at	CG9481	1.036719461	0.000532019	0.020661544	6.145157227	6.40172441	5.931790468	7.001509928	7.506800907	7.080519652	P	P	P	P	P	P
1640167_s_at	CG34104	1.349072318	1.58E-06	0.000421003	10.17003369	10.18956254	10.02294614	11.37238618	11.48723193	11.57014122	P	P	P	P	P	P
1640566_at	CG1944	1.664425213	9.68E-07	0.000282235	4.472578885	4.609202461	4.383175911	6.003482906	6.17956885	6.27518114	A	A	A	P	P	P
1640587_at	CG15214	1.170872466	0.000951861	0.029621774	5.14155032	5.309988198	4.930942098	6.098677302	6.784029791	6.012390919	A	A	A	P	P	P
1640746_at	CG18542	1.146161903	2.43E-06	0.0005758	8.429958509	8.408375452	8.459278323	9.692479852	9.524128633	9.519489507	P	P	P	P	P	P
1641174_at	CG7361	4.400741271	2.72E-08	3.04E-05	4.39316231	3.993173548	3.746222034	8.475428695	8.522522564	8.336830447	A	A	A	P	P	P
1641190_at	CG6580	3.703055158	5.85E-09	1.39E-05	7.318400832	7.371429482	7.624085393	11.26514279	11.05201764	11.10592075	P	P	P	P	P	P
1641235_at	CG14102	1.278728152	9.89E-05	0.006463675	6.46869155	6.390423832	6.627909086	8.049489615	7.437689702	7.836029606	P	P	P	P	P	P
1641245_a_at	character(0)	1.583547811	0.000124773	0.007778581	5.436292627	4.96196582	5.531056811	7.268656276	6.737848797	6.673453616	P	P	P	P	P	P
1641268_at	CG13313	1.739272342	2.42E-05	0.002642525	4.540748557	5.081901258	4.474389715	6.263687626	6.463102909	6.588066023	A	A	A	A	A	A
1641626_at	CG18233	1.920848253	0.000478206	0.019094484	4.126569571	3.736684756	3.937426778	5.18992962	6.528985063	5.844311179	A	A	A	P	P	A
1641729_at	CG13043	1.75818391	5.79E-05	0.004550507	4.814469853	5.275390363	4.736366376	6.321369047	6.878364716	6.901044562	A	A	A	P	P	P
AFFX-Dm-K01486-1_at	character(0)	2.854138677	1.43E-07	9.02E-05	4.055372845	3.76173587	3.869298249	6.646196855	7.00317186	6.599454279	A	A	A	P	P	P
AFFX-Dm-U57609-2_s_at	character(0)	4.900354811	5.21E-10	3.27E-06	4.674778709	4.787779415	4.747117512	9.517859394	9.826438135	9.566442541	A	A	A	P	P	P

DECREASED GENES

ID	Annotation ID	logFC	P.Value	adj.P.Val	X2W1118.A	X2W1118.B	X2W1118.C	CK163.D	CK163.E	CK163.F	2W1118-A.CEL	2W1118-B.CEL	2W1118-C.CEL	CK163-D.CEL	CK163-E.CEL	CK163-F.CEL
1622920_at	CG7875	-1.149896659	2.50E-05	0.002658615	10.59257018	10.57265036	10.52957771	9.168707065	9.523114089	9.553287121	P	P	P	P	P	P
1623051_at	CG18125	-1.89357408	0.001667698	0.041989558	9.438451479	8.131103112	9.730623075	7.071990589	7.278582211	7.268882628	P	P	P	P	P	P
1623211_at	CG17234	-2.235038792	0.001201294	0.034448763	8.622669576	7.48597553	9.370780014	6.153115814	6.192304577	6.428888352	P	P	P	P	P	P
1623346_at	CG2861	-1.001693605	0.002032502	0.04767323	6.113129663	6.544429999	6.377506938	4.956520776	5.748809239	5.32465577	P	P	P	M	P	P
1623410_at	CG3477	-1.823617898	0.001328296	0.036536814	7.247344574	7.165439017	7.125864824	4.451775558	5.699691563	5.9163276	P	P	P	A	A	A
1623477_at	character(0)	-3.029720994	7.88E-07	0.000260441	9.072946869	8.433269495	8.702720593	5.466035397	5.746421651	5.907316927	P	P	P	A	P	A
1623675_at	CG7592	-1.680618681	6.99E-05	0.005174052	13.1068675	13.77975005	13.00578651	11.64488796	11.51170285	11.6939572	P	P	P	P	P	P
1623790_at	CG31901	-2.228355106	1.66E-07	9.54E-05	9.929653418	9.71940181	9.753164142	7.401510452	7.621038538	7.694605063	P	P	P	P	P	P
1623871_at	CG18563	-1.000223071	0.000780309	0.025936863	6.261640543	5.713671741	5.765020847	4.663933347	5.022723351	5.05300722	P	P	P	A	M	M
1624137_at	CG11911	-1.155987921	0.000121456	0.007672753	12.41992803	12.04942865	12.37415423	11.22503348	11.28821438	10.86229929	P	P	P	P	P	P
1624212_at	CG6784	-1.557300642	0.000564832	0.021390103	7.131405858	7.899752534	7.325794056	5.550537311	6.35311035	5.781402863	P	P	P	P	P	P
1624508_at	CG13526	-1.226006906	0.000285765	0.01324163	7.901961451	8.503233616	8.093781118	6.671114848	7.119933895	7.029906725	P	P	P	P	P	P
1624543_s_at	character(0)	-2.067127248	1.98E-07	0.000107058	8.204327932	8.27562032	8.357234942	6.13546777	6.391467478	6.108866201	P	P	P	P	P	P
1624802_at	CG31686	-1.722300347	0.001305007	0.036211554	8.015059999	6.84081966	8.196280093	6.092515748	5.885276375	5.907466589	P	P	P	P	P	P
1624819_s_at	character(0)	-1.913623137	2.26E-05	0.002546964	6.653584593	6.589139807	6.61471132	5.119274698	4.302210362	4.695081251	P	P	P	P	P	P
1624957_a_at	CG4821	-3.127907497	1.52E-07	9.17E-05	10.21051705	10.5784306	10.28439694	7.018149567	7.479945185	7.191527345	P	P	P	P	P	P
1624991_at	CG11611	-3.214374123	4.78E-08	4.32E-05	8.420288317	8.876518176	8.698305394	5.56730232	5.371606282	5.413080917	P	P	P	A	P	M
1625050_s_at	character(0)	-1.494989554	0.000139016	0.008363933	8.551455026	8.505697314	8.58430268	6.627178091	7.477261882	7.052046383	P	P	P	P	P	P
1625124_at	CG10146	-2.482853969	8.22E-05	0.005715502	8.439110244	7.468935321	8.099657835	5.991324086	5.5081003	5.059717108	P	P	P	P	P	M
1625135_at	CG13415	-1.129627384	0.000699264	0.024139248	6.30284609	6.666825614	6.364094339	5.055836303	5.740347423	5.148700167	P	P	P	A	P	A
1625141_at	CG14715	-2.665255328	8.64E-08	6.82E-05	9.36281286	9.060129622	9.310692906	6.68097795	6.413294209	6.643597245	P	P	P	P	P	P
1625195_s_at	CG7734	-1.899412551	6.28E-06	0.001097775	9.590482553	9.722766571	9.590503619	7.453151566	7.679330473	8.073033052	P	P	P	P	P	P
1625287_at	character(0)	-1.057835552	6.96E-05	0.005171237	7.320779405	7.211662177	7.291974863	6.091918495	6.48785456	6.071136734	P	P	P	P	P	P
1625342_at	CG33282	-1.15630994	0.000174126	0.009649236	7.075403723	7.431352285	6.992521688	5.909631173	5.845382809	6.275333896	P	P	P	P	P	P
1625359_at	CG31281	-3.996198295	2.73E-08	3.04E-05	8.630693806	9.159013965	8.693493025	4.844372341	4.913809342	4.73642423	P	P	P	P	M	A
1625375_at	CG32201	-1.456409015	0.00028678	0.013256216	5.126349897	6.017530432	5.354875685	4.023058221	3.959701822	4.146768927	P	P	P	A	A	A
1625527_at	CG5265	-1.538576749	0.000463988	0.018788319	8.868612549	9.379378969	8.968366688	7.092724528	8.038036549	7.46986688	P	P	P	P	P	P
1625648_at	CG3868	-1.379625669	5.83E-07	0.000209618	12.46183359	12.61430986	12.4583016	11.13945284	11.15424944	11.10186575	P	P	P	P	P	P
1625698_at	CG6639	-2.402992606	0.000338084	0.014935601	9.2641211	7.944824947	9.152044475	6.735420626	5.961384464	6.455207613	P	P	P	P	P	P
1625791_s_at	character(0)	-1.5139312	1.86E-06	0.000482701	5.648065794	5.590553047	5.841604043	4.205519121	4.046876861	4.286033302	P	P	P	A	A	A
1625828_at	CG11841	-1.062672688	0.000113769	0.007284288	9.427792816	9.76836451	9.52268096	8.3478578	8.74146293	8.441499492	P	P	P	P	P	P
1625862_x_at	character(0)	-1.10988251	3.33E-05	0.003222748	7.392796425	7.451688436	7.332912635	6.332179416	6.464352465	6.051218084	P	P	P	P	P	P
1625957_at	CG10051	-1.192808201	7.11E-05	0.00520307	6.899247331	6.455257794	6.775622374	5.568111913	5.656962351	5.326628633	P	P	P	P	P	P
1626022_at	CG14680	-1.052718612	0.000129918	0.007942582	7.774609797	8.004882069	7.699992905	6.52449276	6.838292783	6.958543393	P	P	P	P	P	P
1626123_at	character(0)	-1.165535403	0.000552494	0.021025823	8.3999715	8.142595142	8.425749561	7.430187221	6.717291084	7.324231689	P	P	P	P	P	P
1626251_at	CG9668	-1.13013274	6.46E-06	0.001113706	10.14954707	10.12636078	10.05930743	8.841786034	9.116215244	8.986815778	P	P	P	P	P	P
1626257_at	CG13656	-1.315633866	1.36E-05	0.001900535	7.267830187	7.703275346	7.540199698	6.255524025	6.118397384	6.190482223	P	P	P	P	P	P
1626326_at	CG31436	-1.291023621	6.78E-07	0.000233464	8.118867884	8.246726551	8.21288966	6.886067853	6.944468418	6.874876961	P	P	P	P	P	P
1626345_at	CG15231	-1.062729048	0.002080761	0.048393481	10.84998878	10.04722519	10.81676674	9.626904885	9.312505918	9.586382762	P	P	P	P	P	P
1626529_at	CG12977	-1.018360693	0.000123769	0.007741489	7.117521079	7.162206013	7.054668433	5.813650047	6.154783635	6.310879763	P	P	P	P	P	P
1626540_at	CG14076	-1.811523033	0.000237658	0.011680063	7.974688416	8.627445661	8.059341873	5.936435247	6.85264758	6.437824025	P	P	P	P	P	P
1627271_at	CG13422	-1.04434188	0.001603995	0.041137352	11.45446793	10.8367376	11.68808483	10.26848531	10.39116773	10.18661168	P	P	P	P	P	P
1627343_a_at	CG5535	-1.078989377	0.000790002	0.026035091	9.401760564	8.794655372	9.330957253	8.305739788	7.84045182	8.144213449	P	P	P	P	P	P
1627408_at	CG32107	-1.586170766	2.34E-05	0.002619091	6.742273024	7.206838978	6.579053172	5.217283502	5.234997401	5.317371975	P	P	P	P	P	P
1627613_at	CG8175	-2.519717	0.00021159	0.010837965	9.362710245	8.218532658	9.495262051	6.790440252	6.054943051	6.671970649	P	P	P	P	P	P
1627642_at	CG14974	-1.422595157	0.001668327	0.041989558	8.666682336	9.354079359	8.821703299	7.006131526	8.057073802	7.511474196	P	P	P	P	P	P
1627716_at	CG11598	-4.29329256	1.55E-07	9.17E-05	9.089605304	9.801910468	9.362191812	4.878870114	5.39844777	5.09651202	P	P	P	A	A	A
1627745_s_at	character(0)	-1.073456406	3.03E-05	0.003025957	5.503144477	5.172580985	5.168534731	4.19506089	4.280525134	4.148304951	A	A	A	A	A	A

1627759_at	CG30080	-1.036438575	0.00107973	0.03217461	6.710190498	6.021092007	6.466846205	5.240486723	5.618846978	5.229479283	P	P	P	M	P	A
1627771_at	CG13075	-1.811891551	2.54E-06	0.00058679	7.604375791	7.849854839	7.411878609	5.786240023	5.949229879	5.694965233	P	P	P	A	M	M
1628226_at	CG8539	-1.695946427	2.49E-06	0.000581926	7.812741025	7.963250639	7.921573755	6.302272247	5.954315813	6.353138077	P	P	P	P	P	P
1628238_at	CG10541	-1.352576224	0.001339799	0.036622979	9.221297715	9.586598912	9.44375387	7.502309626	8.599930241	8.091681958	P	P	P	P	P	P
1628617_at	CG32282	-3.625097004	1.46E-08	1.97E-05	11.65169328	11.74390638	11.67963557	8.167633856	8.228100945	7.804209419	P	P	P	P	P	P
1628639_at	CG5791	-1.448346125	5.95E-05	0.004635541	10.63036413	10.21069127	10.79319806	8.887038814	9.174677863	9.227498415	P	P	P	P	P	P
1628818_at	CG17562	-1.310530363	3.55E-06	0.000747543	10.30110855	10.49169055	10.20751805	8.94193086	9.093721604	9.033073605	P	P	P	P	P	P
1629014_s_at	CG2604	-1.076529361	4.44E-06	0.000859521	11.16120611	11.29365724	11.1165996	10.16194035	10.13605693	10.04387759	P	P	P	P	P	P
1629083_at	CG16704	-1.076573349	5.33E-06	0.00100059	10.96373098	10.94894094	10.88427686	9.985541522	9.78866214	9.793025065	P	P	P	P	P	P
1629184_at	CG3819	-3.998484061	6.91E-10	3.27E-06	10.63794639	10.62779184	10.80953315	6.764684085	6.626167974	6.688967142	P	P	P	P	P	P
1629233_s_at	CG16708	-3.180590512	9.59E-07	0.000282235	9.423351577	9.255859106	9.373134887	5.691672266	6.470101962	6.348799806	P	P	P	P	P	P
1629367_at	CG15534	-3.645689552	6.62E-09	1.40E-05	9.037763917	9.092288755	8.874838288	5.268558191	5.255676849	5.543587264	P	P	P	A	A	P
1629462_at	CG30192	-1.103978542	0.00054105	0.020835537	8.282133883	8.563094546	8.439986403	7.122458071	7.742412676	7.108408461	P	P	P	P	P	P
1629530_at	CG15066	-1.503071341	6.29E-05	0.004768576	11.34823078	10.88274816	11.54666755	9.585448135	9.856125286	9.826859046	P	P	P	P	P	P
1629588_at	CG14240	-1.505938035	1.58E-05	0.002070892	6.567378896	7.005797514	6.944309846	5.500999329	5.318816395	5.179856426	P	P	P	P	P	P
1629641_s_at	character(0)	-1.080782012	1.42E-05	0.001925701	5.136320562	5.140356499	5.16739725	4.184203318	3.875252748	4.142272211	P	P	P	A	A	A
1629738_at	CG14957	-1.582256537	4.23E-05	0.00369017	5.671870954	5.796915466	5.192542882	4.150907576	3.976535266	3.787116848	P	P	M	A	A	A
1629846_at	CG11619	-1.927735357	9.08E-07	0.000273161	7.76366522	7.503848038	7.427778248	5.528939728	5.792529892	5.590615816	P	P	P	P	P	A
1629934_at	CG14219	-1.205556971	0.000663289	0.02345272	6.112171827	6.828436541	6.16770611	4.930293379	5.284816458	5.276533727	P	P	P	A	M	P
1630010_a_at	CG17077	-1.766290943	2.51E-07	0.000118836	7.629147765	7.388820477	7.590084918	5.740592276	5.754372088	5.814215966	P	P	P	P	P	P
1630119_s_at	CG3620	-2.19047342	3.35E-08	3.34E-05	7.793895568	7.889450603	7.857543357	5.671055851	5.746372634	5.552040783	P	P	P	P	P	P
1630154_at	CG3437	-1.921827633	5.11E-05	0.004268949	7.779432618	7.284395586	7.608356122	6.016187279	5.21414198	5.676372166	P	P	P	A	A	A
1630419_a_at	CG7300	-1.175290408	6.73E-06	0.001138709	8.288420279	8.207063147	8.50365398	7.151336024	7.11738143	7.204548729	P	P	P	P	P	P
1630528_at	CG17751	-1.691779169	1.69E-05	0.002183219	8.732346602	9.180195106	8.695439058	7.377092226	7.180322291	6.975228741	P	P	P	P	P	P
1630614_s_at	character(0)	-1.905536258	2.86E-07	0.000123001	9.633796772	9.545175475	9.438719671	7.642545717	7.496567947	7.761969479	P	P	P	P	P	P
1630700_at	character(0)	-1.522001544	4.01E-05	0.003564137	6.90855135	6.930101628	7.298019293	5.252740309	5.791568513	5.526358817	P	P	P	P	P	A
1630734_at	CG6240	-1.426595546	5.51E-06	0.001023826	7.528565077	7.279978318	7.5225033	5.904311726	5.949418869	6.197529463	P	P	P	P	P	P
1630938_a_at	CG14879	-2.503206018	3.95E-08	3.74E-05	8.140442492	8.00065249	7.962660558	5.385359105	5.573725761	5.635052622	P	P	P	P	P	P
1631475_at	CG7629	-2.075346182	0.001018591	0.030986109	5.691108755	7.352474939	6.130442565	4.345921576	4.371259857	4.230806279	P	P	P	A	A	A
1632045_at	CG5697	-1.230392272	0.00062653	0.022788017	9.290231324	10.04016757	9.26089072	8.197702582	8.310375782	8.392034429	P	P	P	P	P	P
1632215_at	CG6296	-1.403699888	1.76E-05	0.002198049	6.666361798	6.778908234	6.656552103	5.416068719	5.473942998	5.000710753	P	P	P	A	M	A
1632656_at	CG12112	-1.091618371	0.000727476	0.024752483	8.510434523	8.028562414	8.392918615	7.547335276	6.909536994	7.200188171	P	P	P	P	P	P
1632720_at	CG9120	-1.360694197	0.000281689	0.013084733	7.500924836	7.091847266	7.755317072	6.41171644	5.939333832	5.914956311	P	P	P	P	P	M
1632870_at	CG32364	-1.844563776	9.96E-05	0.006488539	8.688127093	8.26268786	8.587292317	7.000183154	6.14596219	6.858270597	P	P	P	P	P	P
1632964_at	CG15678	-1.095314294	0.001437214	0.038309525	8.512888807	7.895968586	7.603300993	6.956894365	6.890549631	6.878771507	P	P	P	P	P	P
1632978_at	CG32207	-1.426529471	7.30E-05	0.005284743	6.795963371	6.241093228	6.850347357	5.254062205	5.292178226	5.061575113	P	P	P	P	A	P
1633053_at	CG18108	-1.413188434	0.000325612	0.014544153	11.10157553	10.58413607	11.45289986	9.585945329	9.817939227	9.495161601	P	P	P	P	P	P
1633481_at	CG14394	-1.174137922	2.40E-06	0.0005758	7.621800476	7.670650257	7.483291433	6.46564098	6.365239377	6.422448043	P	P	P	P	P	P
1633599_a_at	CG17725	-1.037158336	0.000828446	0.026884764	13.00973414	13.20359824	12.86821453	11.62837466	12.00373471	12.33796253	P	P	P	P	P	P
1633709_at	CG2772	-1.039196272	8.95E-06	0.001401642	7.574509634	7.743033653	7.532459635	6.574116408	6.505119173	6.653178524	P	P	P	P	P	M
1633826_at	CG4715	-2.130620797	7.22E-06	0.001200384	9.448786363	9.558213461	9.480596674	7.384470366	7.731334535	6.979929207	P	P	P	P	P	P
1633946_at	CG31955	-2.206803667	2.59E-07	0.000119507	7.670354328	7.733920803	7.535020404	5.642969887	5.379662228	5.296252421	P	P	P	P	P	P
1634016_at	CG2781	-5.977700252	1.61E-09	5.07E-06	10.3034299	10.58357143	10.59860285	4.798134452	4.32005958	4.434309387	P	P	P	P	A	A
1634076_at	CG12057	-1.138384028	2.72E-05	0.002848133	12.78714035	12.48763614	12.65726941	11.67829492	11.4671466	11.37145229	P	P	P	P	P	P
1634239_at	CG14205	-2.059139045	3.01E-07	0.000123885	7.940751294	7.751677487	7.817612023	5.672221025	5.969451017	5.690951627	P	P	P	A	A	A
1634409_at	CG31775	-1.178749673	0.000534971	0.0207191	9.126355246	9.182408562	9.13384658	8.423961493	7.605162841	7.877237035	P	P	P	P	P	P
1634671_a_at	character(0)	-1.145952309	0.001029918	0.031168984	5.640345906	4.736209108	5.253920912	4.031009844	4.014114803	4.147494353	P	P	P	A	A	A
1635065_at	CG7025	-1.083956991	1.37E-05	0.001900535	9.052749078	9.03210427	8.796910282	7.870031363	7.968402536	7.791458757	P	P	P	P	P	P
1635200_at	CG11261	-1.067859121	8.14E-05	0.00569423	6.490914455	6.786948009	6.574017233	5.775530025	5.486997318	5.385774992	P	P	P	P	P	P
1635263_at	CG11825	-1.007456003	0.000456715	0.018694095	8.174314774	7.816729077	8.229635701	7.356304005	6.917304725	6.924702813	P	P	P	P	P	P
1635270_at	character(0)	-1.903751439	3.31E-06	0.000715581	8.009208016	8.130570753	7.639234137	5.906994303	6.162466046	5.998298242	P	P	P	A	P	A
1635276_at	CG11437	-2.072577437	7.50E-06	0.001235708	7.451784052	7.885374786	7.189603758	5.558226764	5.323572813	5.427230709	P	P	P	A	A	A
1635507_at	CG4269	-1.119008367	1.31E-05	0.001865825	9.381469807	9.228673849	9.141421494	8.168822054	8.235466523	7.990251471	P	P	P	P	P	P
1635512_at	CG11893	-4.126967454	1.37E-09	5.07E-06	7.857651023	8.125934994	7.931620794	3.745756419	3.868304764	3.920243268	P	P	P	A	A	A
1635650_at	character(0)	-1.041316636	3.95E-05	0.003538877	5.735186909	5.962363755	5.654490489	4.626680545	4.885405441	4.716005261	P	P	P	P	P	A
1635671_at	CG33290	-2.036524494	0.000213222	0.010852713	8.061453938	8.949548863	7.981153339	6.0552306	6.704370327	6.122981732	P	P	M	A	A	A
1635793_at	CG11413	-1.068925509	2.99E-05	0.003013807	10.34617278	10.45171432	10.23988187	9.270787664	9.109021441	9.451183342	P	P	P	P	P	P
1636015_s_at	CG32850	-1.118462241	3.52E-05	0.003318224	9.760879253	9.732032868	9.948068651	8.501955342	8.88492127	8.698717437	P	P	P	P	P	P
1636105_at	CG13675	-2.144029916	7.83E-06	0.001279437	6.144401146	6.881895791	6.630428135	4.410967924	4.304812929	4.508854471	P	P	P	A	A	A
1636490_at	PGRP-SB1	-1.336412633	8.41E-06	0.001328147	9.893716535	10.25533893	10.20584796	8.690550937	8.83473939	8.820375199	P	P	P	P	P	P
1636566_at	CG32368	-2.831968347	2.10E-05	0.002407133	7.579398877	6.64849359	7.78147261	4.601958315	4.582745066	4.328756655	P	P	P	A	P	A
1636678_at	CG14343	-1.103214834	2.44E-05	0.002642525	5.738409503	5.812769762	6.047973118	4.878162732	4.773362808	4.63798234	A	P	P	A	A	A
1636829_at	CG31902	-1.23397477	0.0006613	0.02345272	6.952795877	7.563968545	7.120509587	5.668545767	6.329509261	5.937294671	P	P	P	M	P	A
1637095_at	CG15818	-1.255221225	0.000309672	0.013973565	7.197560672	6.695845924	7.455962398	5.731341424	5.880819544	5.971544351	P	P	P	P	P	A
1637275_a_at	CG13335	-1.081378986	0.001251435	0.035611404	11.4566839	11.69344335	11.63243635	10.02582675	10.67254836	10.84005153	P	P	P	P	P	P
1637574_at	CG1966	-1.074504253	0.000997902	0.030552898	9.634101214	9.172283651	9.631907976	8.722195199	8.06182821	8.430756673	P	P	P	P	P	P
1637590_at	CG5156	-2.461443959	2.09E-07	0.000108057	7.67162819	7.490753374	7.743453391	5.240321132	4.955240516	5.32594143	P	P	P	A	P	A
1637691_at	CG4570	-3.468944638	5.47E-07	0.000203368	8.284054411	7.769582599	8.514699179	4.946912731	4.634959309	4.579630235	P	P	P	P	A	A

1637833_at	CG30095	-1.144256408	1.72E-05	0.002184696	6.951229225	7.237476963	6.870283229	5.910067284	5.876574725	5.839578182	P	P	P	P	P	P
1637900_at	CG11852	-1.005881907	2.90E-05	0.002982708	9.625336758	9.897039911	9.716428907	8.766646029	8.840451916	8.614061911	P	P	P	P	P	P
1637963_at	CG14191	-1.138554106	9.99E-06	0.001539743	7.424870998	7.412587357	7.531884469	6.206298677	6.494662308	6.252719521	P	P	P	P	P	P
1638016_at	CG2993	-1.280649851	1.69E-05	0.002183219	6.511596924	6.186373939	6.590435531	5.115322314	5.080001478	5.251133047	P	P	P	P	P	P
1638053_at	CG10842	-2.065085307	3.08E-07	0.000124043	9.966262249	10.10097967	9.919301412	7.968331842	7.74497226	8.077983312	P	P	P	P	P	P
1638054_at	CG13056	-1.311039119	8.60E-07	0.000267275	7.474141143	7.442707644	7.382006747	6.15713247	6.181399259	6.027206449	P	P	P	M	A	M
1638096_at	CG14315	-1.037299698	0.000155717	0.008991422	5.206364829	5.451106411	5.694651863	4.2664606	4.537380249	4.436383161	P	P	P	A	M	A
1638139_at	CG31664	-1.725679677	1.03E-05	0.001578556	6.455996523	6.689837954	6.38322642	4.826178946	5.030940892	4.49490203	P	P	P	A	A	A
1638142_at	CG3288	-1.036825828	0.00012864	0.007889929	5.55627385	5.983177844	5.692325406	4.53259349	4.752392727	4.8363134	P	P	P	A	P	A
1638235_at	CG10794	-2.331740326	7.03E-05	0.0051849	9.280479697	8.735095141	9.869465578	6.697882539	7.180781513	7.011155385	P	P	P	A	A	A
1638275_at	CG11699	-1.067172061	9.69E-06	0.001505892	10.89932527	10.92037822	10.81865954	9.969050246	9.727056914	9.740739687	P	P	P	P	P	P
1638484_at	CG4190	-1.751021886	8.82E-07	0.00026964	7.721781692	7.638458489	7.38284232	5.768164991	5.908746147	5.813105705	P	P	P	P	P	P
1638528_at	CG15531	-1.038855564	1.99E-05	0.002356764	11.3137666	11.52188956	11.19780179	10.25393334	10.35709909	10.30585882	P	P	P	P	P	P
1638616_at	CG17084	-1.469584655	1.18E-05	0.001735154	7.212493569	7.594345153	7.217302164	5.706976985	5.958371469	5.950038467	P	P	P	P	P	P
1638696_a_at	CG3552	-1.01038418	0.001199991	0.034448763	9.592747404	9.783544363	9.592326041	8.250528798	9.041390396	8.645546076	P	P	P	P	P	P
1638716_a_at	CG10725	-4.587719153	8.97E-09	1.55E-05	9.707107036	9.326242829	9.727870053	5.215311247	4.898272133	4.88447908	P	P	P	A	P	P
1638778_at	CG10352	-1.421114549	4.65E-06	0.000881182	7.08706666	7.290440584	6.9822046	5.696320359	5.828448233	5.571599606	P	P	P	A	P	A
1638974_at	CG14963	-1.70112185	1.36E-06	0.000372782	7.5083963	7.269220615	7.368366162	5.852655201	5.659494584	5.530467741	P	P	P	P	P	P
1639019_s_at	CG33470	-1.185375713	0.000446023	0.018416184	11.51662722	11.53767228	11.93555764	10.095871	10.66832743	10.66953157	P	P	P	P	P	P
1639211_at	character(0)	-2.311089775	3.18E-06	0.000701433	8.771626348	8.587168815	8.582541021	6.573652888	5.939131532	6.495282439	P	P	P	P	P	P
1639278_at	CG18105	-1.183432532	2.62E-06	0.000597752	6.697556059	6.846276136	6.638220888	5.550321429	5.495559286	5.585874773	P	P	P	A	P	P
1639365_at	CG33495	-2.008231778	1.09E-05	0.001635711	11.59031313	11.35420349	11.16490036	9.210657754	9.724920186	9.149143708	P	P	P	P	P	P
1639429_at	Oatp58Da	-1.074795779	3.44E-05	0.003277602	7.646941012	8.047419826	7.750269995	6.735212729	6.712119519	6.772911246	P	P	P	P	P	P
1639454_at	CG10912	-1.024427923	8.59E-05	0.005901107	10.76377096	10.78389061	10.76011109	9.593876029	9.617227109	10.02338576	P	P	P	P	P	P
1639571_s_at	CG18743	-2.357056833	0.001426978	0.038234224	9.008653681	7.17979922	7.387338443	5.694033943	5.65949382	5.151093082	P	P	P	P	P	P
1639914_at	CG5961	-1.619361046	2.42E-06	0.0005758	8.751248807	8.603357594	8.761941829	7.199884553	6.861621455	7.196959083	P	P	P	P	P	P
1639986_at	CG6854	-1.533091078	3.88E-05	0.00349936	7.855866392	7.363251231	7.832915374	6.04934661	6.011623135	6.391790017	P	P	P	P	P	P
1640004_at	CG9726	-1.720412198	5.99E-06	0.001070262	6.55539226	6.420153528	6.549965492	4.616901424	4.646430473	5.100942788	P	P	P	A	A	A
1640075_a_at	CG3424	-2.120409487	1.43E-07	9.02E-05	9.511591271	9.48841725	9.740704467	7.381053181	7.560138173	7.438293174	P	P	P	P	P	P
1640400_at	CG5773	-1.011387497	8.10E-05	0.00569423	10.85741783	11.11008279	10.78322162	10.04590969	9.938996468	9.731653582	P	P	P	P	P	P
1640404_at	CG1076	-1.092287088	0.000912266	0.028624606	6.821434043	7.409775349	6.929547876	5.630923134	6.153392301	6.099580567	P	P	P	P	P	P
1640416_at	character(0)	-1.411774141	9.19E-05	0.006195013	5.344759672	6.006636676	5.565912217	4.313184183	4.3185909	4.05021106	P	P	A	A	A	A
1640642_at	CG5192	-1.025952478	2.06E-05	0.002401486	10.23231839	10.29673085	10.10525544	9.026532408	9.285698878	9.244215951	P	P	P	P	P	P
1640666_at	CG31205	-1.905620262	1.99E-06	0.000509009	11.0658503	11.29628448	11.02425475	9.0994703	9.470577088	9.099481359	P	P	P	P	P	P
1640965_at	CG10050	-1.146396084	0.000300328	0.013714269	6.437208056	5.909725746	6.208498505	5.317822259	4.95159073	4.846831065	P	P	P	P	P	A
1641084_at	CG13897	-1.027261606	0.000441558	0.018343652	7.511608587	7.17181675	7.184453514	6.505845446	5.951402178	6.32884641	P	P	P	P	P	P
1641270_at	CG8745	-2.526190529	1.38E-06	0.000374311	10.70904893	10.34700136	10.44452124	7.687272416	7.961809932	8.272917594	P	P	P	P	P	P
1641419_at	CG4740	-2.781374143	3.25E-05	0.003196491	9.951408736	8.964628068	10.18180982	6.810197142	7.037287991	6.906239058	P	P	P	P	P	P
1641464_s_at	CG32850	-1.676783484	2.22E-07	0.000108057	9.307134752	9.264613906	9.276486176	7.483690434	7.696140537	7.638053412	P	P	P	P	P	P
1641738_a_at	CG13636	-1.520492011	2.09E-06	0.000528284	9.558420242	9.197911102	9.314058567	7.804474336	7.847947599	7.856491941	P	P	P	P	P	P
1641746_at	CG2555	-1.490535792	1.74E-05	0.002184696	6.25896689	5.888182561	6.357740851	4.701112194	4.812522657	4.519648075	P	P	P	A	A	A
