# Supplementary material for: Survival Response to Increased Ceramide Involves Metabolic Adaptation through Novel Regulators of Glycolysis and Lipolysis
Source: PLoS Genet. 2013 Jun 20;9(6):e1003556. doi: 10.1371/journal.pgen.1003556 (PMC3688504; doi:10.1371/journal.pgen.1003556)
Supplement: Table S2 — List of candidate genes from microarray analysis that contain FOXO binding sites. The table shows genes from the microarray analysis for which potential FOXO binding sites were identified. The columns show FBgn ID with chromosome location, strand, gene start and end positions, CG numbers, peak start and end positions, distance to peak, shortest distance to peak and whether it is overlapping or nearest. (PDF) [file pgen.1003556.s011.pdf]

Feature	CG	Peak start	Peak end	Distance to Feature	Shortest Distance	From Overlapping Or Nearest
FBgn0014417,chr2L,-,8409050,8411868	CG13397	8406938	8408130	4334	920	NearestStart
FBgn0000351,chr2L,-,6674636,6676635	CG11330	6679868	6680454	-3526	3233	NearestStart
FBgn0034437,chr2R,+,14878507,14882157	CG10051	14875548	14876748	-2359	1759	NearestStart
FBgn0033674,chr2R,-,7539052,7541177	CG8964	7545060	7548984	-5845	3883	NearestStart
FBgn0033999,chr2R,-,10729194,10730463	CG8093	10725084	10727488	4177	1706	NearestStart
FBgn0035412,chr3L,+,3171110,3171522	CG14957	3167466	3169570	-2592	1540	NearestStart
FBgn0023479,chr3L,+,9048604,9066072	CG4821	9042480	9045180	-4774	3424	NearestStart
FBgn0037030,chr3L,+,20770051,20770796	CG3288	20772778	20773376	3026	1982	NearestStart
FBgn0053290,chr3L,+,21476269,21476785	CG33290	21476566	21477160	594	219	NearestStart
FBgn0053290,chr3L,+,21476269,21476785	CG33290	21478942	21481068	3736	2157	NearestStart
FBgn0035131,chr3L,-,313351,315838	CG17084	317770	318360	-2227	1932	NearestStart
FBgn0035998,chr3L,-,9430283,9431608	CG3437	9426446	9427658	4556	2625	NearestStart
FBgn0036204,chr3L,-,11748080,11748359	CG11611	11752380	11752960	-4311	4021	NearestStart
FBgn0036204,chr3L,-,11748080,11748359	CG11611	11753550	11754158	-5495	5191	NearestStart
FBgn0003076,chr3L,-,16091313,16093855	CG5165	16089146	16089768	4398	1545	NearestStart
FBgn0003076,chr3L,-,16091313,16093855	CG5165	16090342	16091582	2893	269	NearestStart
FBgn0036648,chr3L,-,16573396,16574479	CG4098	16571562	16572454	2471	942	NearestStart
FBgn0036648,chr3L,-,16573396,16574479	CG4098	16572776	16573942	1120	537	NearestStart
FBgn0036764,chr3L,-,17817882,17820947	CG5535	17818080	17818642	2586	198	NearestStart
FBgn0020277,chr3L,-,19569655,19571349	CG8807	19573042	19574842	-2593	1693	NearestStart
FBgn0020277,chr3L,-,19569655,19571349	CG8807	19579342	19582944	-9794	7993	NearestStart
FBgn0036906,chr3L,-,19732820,19735129	CG14102	19730852	19732344	3531	476	NearestStart
FBgn0036906,chr3L,-,19732820,19735129	CG14102	19732964	19734770	1262	144	NearestStart
FBgn0036906,chr3L,-,19732820,19735129	CG14102	19743144	19743748	-8317	8015	NearestStart
FBgn0036764,chr3L,-,17817882,17822023	CG5535	17818080	17818642	479	198	Overlapping
FBgn0053192,chr3R,+,16360726,16361007	CG33192	16364130	16365634	4156	3123	NearestStart
FBgn0039628,chr3R,+,24876792,24878047	CG11841	24867630	24869468	-8243	7324	NearestStart
FBgn0039628,chr3R,+,24876792,24878047	CG11841	24871540	24872442	-4801	4350	NearestStart
FBgn0027620,chr3R,+,27619810,27625533	CG1966	27609638	27616862	-6560	2948	NearestStart
FBgn0027620,chr3R,+,27619810,27625533	CG1966	27618030	27618632	-1479	1178	NearestStart
FBgn0037930,chr3R,+,7499940,7500654	CG14715	7502440	7503044	2802	1786	NearestStart
FBgn0038047,chr3R,+,8255391,8256897	CG5245	8245554	8246444	-9392	8947	NearestStart
FBgn0038067,chr3R,+,8460550,8461924	CG11598	8454634	8455266	-5600	5284	NearestStart
FBgn0038419,chr3R,-,12171851,12174946	CG14879	12175252	12177050	-1205	306	NearestStart
FBgn0003358,chr3R,-,25750946,25751907	CG31039	25759568	25760738	-8246	7661	NearestStart
FBgn0037534,chr3R,-,3807538,3821568	CG2781	3830138	3831330	-9166	8570	NearestStart
FBgn0053105,chr3R,-,5517624,5518516	CG33105	5512868	5514954	4605	2670	NearestStart
FBgn0053105,chr3R,-,5517624,5518516	CG33105	5519130	5522446	-2272	614	NearestStart
FBgn0053105,chr3R,-,5517624,5518516	CG33105	5524536	5525130	-6317	6020	NearestStart
FBgn0037847,chr3R,-,6689070,6694379	CG6584	6696334	6696966	-2271	1955	NearestStart

FBgn0038056,chr3R,-,8284140,8285922	CG5961	8283330	8283950	2282	190	NearestStart
FBgn0038056,chr3R,-,8284140,8285922	CG5961	8287540	8289064	-2380	1618	NearestStart
FBgn0038243,chr3R,-,10393331,10394532	CG8066	10397430	10400154	-4260	2898	NearestStart
FBgn0004369,chr3R,+,25287534,25310461	CG11516	25307436	25308332	2577	2129	Overlapping
FBgn0004369,chr3R,+,25287534,25310461	CG11516	25308934	25309532	1228	929	Overlapping
FBgn0052006,chr4,+,171016,173799	CG32006	160834	162966	-9116	8050	NearestStart
FBgn0052850,chr4,+,335195,341637	CG32850	329130	330348	-5456	4847	NearestStart
