# Supplementary material for: Survival Response to Increased Ceramide Involves Metabolic Adaptation through Novel Regulators of Glycolysis and Lipolysis
Source: PLoS Genet. 2013 Jun 20;9(6):e1003556. doi: 10.1371/journal.pgen.1003556 (PMC3688504; doi:10.1371/journal.pgen.1003556)
Supplement: Table S3 — Fold change ( dcerk1/w1118 ) of metabolites and their classification into different pathways. The table shows relative changes in different metabolites between control and mutant flies. The metabolites have been classified into functional pathways. A p value of 0.05 or less and q value (false discovery rate) of 0.1 or less is considered statistically significant. (PDF) [file pgen.1003556.s012.pdf]

SUPER PATHWAY	PATHWAY	METABOLITE	*dcerk*[1] / *w*[1118]	p value	q value
Amino acid	Alanine and aspartate metabolism	3-ureidopropionate	1.56	0.0200	0.0481
Amino acid	Alanine and aspartate metabolism	beta-alanine	0.78	0.2845	0.1997
Amino acid	Butanoate metabolism	2-aminobutyrate	0.99	0.7612	0.3937
Amino acid	Cysteine, methionine, SAM, taurine metabolism	homocysteine	1.12	0.0729	0.0918
Amino acid	Cysteine, methionine, SAM, taurine metabolism	methionine sulfoxide	1.24	0.0441	0.0666
Amino acid	Cysteine, methionine, SAM, taurine metabolism	S-adenosylhomocysteine (SAH)	0.78	0.1850	0.1512
Amino acid	Cysteine, methionine, SAM, taurine metabolism	taurine	1.24	0.0257	0.0524
Amino acid	Glutamate metabolism	gamma-aminobutyrate (GABA)	0.83	0.3482	0.2251
Amino acid	Glutamate metabolism	N-acetylglutamate	1.18	0.2631	0.1877
Amino acid	Glutathione metabolism	5-oxoproline	1.06	0.3327	0.2193
Amino acid	Glycine, serine and threonine metabolism	beta-hydroxypyruvate	1.00	0.9981	0.4612
Amino acid	Glycine, serine and threonine metabolism	betaine	1.51	0.0056	0.0395
Amino acid	Glycine, serine and threonine metabolism	homoserine (homoserine lactone)	0.94	0.5637	0.3101
Amino acid	Guanidino and acetamido metabolism	4-guanidinobutanoate	1.06	0.2456	0.1802
Amino acid	Histidine metabolism	1-methylhistidine	1.21	0.0515	0.0716
Amino acid	Histidine metabolism	histamine	0.84	0.0501	0.0706
Amino acid	Lysine metabolism	pipecolate	0.77	0.3572	0.2285
Amino acid	Phenylalanine & tyrosine metabolism	phenylalanine	0.98	0.7679	0.3956
Amino acid	Polyamine metabolism	5-methylthioadenosine (MTA)	0.73	0.0010	0.0306
Amino acid	Polyamine metabolism	putrescine	0.65	0.0935	0.1045
Amino acid	Polyamine metabolism	spermidine	0.85	0.4110	0.2529
Amino acid	Urea cycle; arginine-, proline-, metabolism	dimethylarginine	1.23	0.1075	0.1126
Amino acid	Urea cycle; arginine-, proline-, metabolism	N-acetylarginine	0.45	0.0124	0.0444
Amino acid	Urea cycle; arginine-, proline-, metabolism	ornithine	0.78	0.1771	0.1493
Amino acid	Urea cycle; arginine-, proline-, metabolism	sarcosine (N-Methylglycine)	0.42	0.0095	0.0437
Amino acid	Urea cycle; arginine-, proline-, metabolism	urea	1.11	0.6287	0.3359
Amino acid	Valine, leucine and isoleucine metabolism	3-methyl-2-oxobutyrate	0.51	0.0344	0.0607
Amino acid	Valine, leucine and isoleucine metabolism	3-methyl-2-oxovalerate	0.62	0.0228	0.0493
Amino acid	Valine, leucine and isoleucine metabolism	4-methyl-2-oxopentanoate	0.43	0.0013	0.0306
Amino acid	Aminosugars metabolism	erythronate*	1.44	0.2818	0.1989
Carbohydrate	Starch, and sucrose metabolism	mannose-6-phosphate	0.76	0.2764	0.1961
Carbohydrate	Nucleotide sugars, pentose metabolism	6-phosphogluconate	1.18	0.6008	0.3250
Carbohydrate	Nucleotide sugars, pentose metabolism	arabitol	3.41	0.0178	0.0453
Carbohydrate	Nucleotide sugars, pentose metabolism	gluconate	0.98	0.7544	0.3918
Carbohydrate	Nucleotide sugars, pentose metabolism	ribitol	5.77	0.0018	0.0306
Carbohydrate	Nucleotide sugars, pentose metabolism	ribose	0.93	0.6817	0.3594
Carbohydrate	Nucleotide sugars, pentose metabolism	ribulose 5-phosphate	0.75	0.3399	0.2221
Carbohydrate	Nucleotide sugars, pentose metabolism	sedoheptulose-7-phosphate	1.27	0.1141	0.1167
Carbohydrate	Nucleotide sugars, pentose metabolism	xylitol	0.88	0.2887	0.2005
Carbohydrate	Folate metabolism	biopterin	0.88	0.4161	0.2536
Carbohydrate	Folate metabolism	dihydrobiopterin	0.79	0.0375	0.0624
Cofactors and vitamins	Pantothenate and CoA metabolism	acetyl coenzyme A	0.93	0.9216	0.4409
Cofactors and vitamins	Pantothenate and CoA metabolism	coenzyme a	1.51	0.0414	0.0640
Cofactors and vitamins	Pantothenate and CoA metabolism	pantothenate	1.69	0.0149	0.0453
Cofactors and vitamins	Pantothenate and CoA metabolism	phosphopantetheine	1.20	0.2414	0.1791
Cofactors and vitamins	Pterins	isoxanthopterin	0.77	0.0178	0.0453
Cofactors and vitamins	Pyridoxal metabolism	pyridoxal	1.20	0.0585	0.0759
Cofactors and vitamins	Riboflavin metabolism	flavin adenine dinucleotide (FAD)	0.75	0.0483	0.0704
Cofactors and vitamins	Riboflavin metabolism	riboflavin (Vitamin B2)	0.79	0.1125	0.1159
Cofactors and vitamins	Thiamine metabolism	thiamin (Vitamin B1)	0.75	0.1929	0.1518
Cofactors and vitamins	Vitamin B6 metabolism	pyridoxate	0.53	0.0078	0.0407
Energy	Oxidative phosphorylation	acetylphosphate	0.92	0.5600	0.3094
Energy	Oxidative phosphorylation	phosphate	1.02	0.8529	0.4247
Energy	Carnitine metabolism	3-dehydrocarnitine*	1.12	0.3264	0.2167
Energy	Carnitine metabolism	acetylcarnitine	0.93	0.1089	0.1131
Energy	Carnitine metabolism	carnitine	1.13	0.3769	0.2376
Energy	Carnitine metabolism	isobutyrylcarnitine	1.05	0.8656	0.4270
Lipid	Carnitine metabolism	oleoylcarnitine	1.33	0.3173	0.2140
Lipid	Carnitine metabolism	palmitoylcarnitine	1.23	0.4971	0.2855
Lipid	Essential fatty acid	linolenate [alpha or gamma; (18:3n3 or 6)]	1.38	0.0177	0.0453
Lipid	Fatty acid, dicarboxylate	adipate	1.48	0.4031	0.2492
Lipid	Fatty acid, dicarboxylate	hexadecanedioate	1.75	0.0008	0.0306
Lipid	Fatty acid, dicarboxylate	sebacate (decanedioate)	0.96	0.8252	0.4137
Lipid	Fatty acid, monohydroxy	4-hydroxybutyrate (GHB)	1.35	0.3225	0.2159
Lipid	Fatty alcohol, long chain	1-octadecanol	1.30	0.2387	0.1782

Lipid	Glycerolipid metabolism	choline	0.85	0.0160	0.0453
Lipid	Glycerolipid metabolism	choline phosphate	1.01	0.8109	0.4091
Lipid	Glycerolipid metabolism	ethanolamine	0.78	0.3628	0.2309
Lipid	Glycerolipid metabolism	glycerol 3-phosphate (G3P)	0.79	0.1302	0.1229
Lipid	Glycerolipid metabolism	glycerophosphorylcholine (GPC)	1.31	0.0364	0.0624
Lipid	Glycerolipid metabolism	phosphoethanolamine	1.05	0.8584	0.4254
Lipid	Glycerolipid metabolism	phosphoglycerate (2 or 3)	0.82	0.3707	0.2348
Lipid	Inositol metabolism	inositol 1-phosphate (I1P)	1.27	0.2112	0.1652
Lipid	Inositol metabolism	myo-inositol	1.23	0.1183	0.1173
Lipid	Ketone bodies	3-hydroxybutyrate (BHBA)	1.12	0.1875	0.1512
Lipid	Mevalonate metabolism	malonate (propanedioate)	0.30	0.1920	0.1518
Lipid	Mevalonate metabolism	mevalonate*	0.64	0.0346	0.0607
Lipid	Phospholipid metabolism	cytidine 5'-diphosphocholine	0.73	0.0838	0.0969
Lipid	Phospholipid metabolism	cytidine-5'-diphosphoethanolamine	0.99	0.9744	0.4518
Lipid	Sterol/Steroid	ergosterol	1.14	0.4578	0.2712
Lipid	Purine metabolism, (hypo)xanthine/inosine containing	hypoxanthine	0.95	0.6129	0.3302
Lipid	Purine metabolism, (hypo)xanthine/inosine containing	inosine	0.90	0.1044	0.1102
Lipid	Purine metabolism, (hypo)xanthine/inosine containing	xanthine	1.27	0.1159	0.1171
Lipid	Purine metabolism, (hypo)xanthine/inosine containing	xanthosine	0.89	0.3812	0.2391
Nucleotide	Purine metabolism, adenine containing	1-methyladenine	0.67	0.0402	0.0629
Nucleotide	Purine metabolism, adenine containing	1-methyladenosine	0.98	0.9628	0.4518
Nucleotide	Purine metabolism, guanine containing	1-methylguanosine	0.78	0.0518	0.0716
Nucleotide	Purine metabolism, guanine containing	guanosine	0.81	0.0851	0.0969
Nucleotide	Purine metabolism, guanine containing	N6-carbamoylthreonyladenosine	0.83	0.1864	0.1512
Nucleotide	Purine metabolism, urate metabolism	allantoin	0.45	0.0112	0.0441
Nucleotide	Purine metabolism, urate metabolism	urate	0.57	0.0389	0.0624
Nucleotide	Pyrimidine metabolism, cytidine containing	5-methylcytidine	0.54	0.0463	0.0683
Nucleotide	Pyrimidine metabolism, cytidine containing	cytidine	1.22	0.0777	0.0951
Nucleotide	Pyrimidine metabolism, uracil containing	pseudouridine	0.80	0.1172	0.1171
Nucleotide	Pyrimidine metabolism, uracil containing	uracil	0.87	0.1556	0.1390
Nucleotide	Pyrimidine metabolism, uracil containing	uridine	0.61	0.0061	0.0396
Nucleotide	Dipeptide	glutamylvaline	1.02	0.8074	0.4091
Nucleotide	Dipeptide	glycylleucine	1.14	0.1849	0.1512
Nucleotide	Dipeptide	glycylproline	1.61	0.0378	0.0624
Nucleotide	Dipeptide	glycylvaline	2.11	0.0002	0.0232
Peptide	Dipeptide	prolylleucine	1.54	0.0339	0.0607
Peptide	γ–glutamyl	gamma-glutamylglutamine	0.75	0.0496	0.0706
Peptide	γ-glutamyl	gamma-glutamylisoleucine*	1.27	0.0073	0.0407
Peptide	γ-glutamyl	gamma-glutamylleucine	1.01	0.9726	0.4518
Peptide	γ-glutamyl	gamma-glutamylphenylalanine	1.13	0.4746	0.2776
Peptide	Benzoate metabolism	4-hydroxybenzoate	0.84	0.4317	0.2607
Peptide	Benzoate metabolism	methyl-4-hydroxybenzoate	0.69	0.2481	0.1810
Peptide	Chemical	glycerol 2-phosphate	1.44	0.0224	0.0493
Peptide	Chemical	glycolate (hydroxyacetate)	1.01	0.9236	0.4409
Xenobiotics	Sugar, sugar substitute, starch	erythritol	1.15	0.4521	0.2705
Xenobiotics	Sugar, sugar substitute, starch	glucoheptose	1.21	0.4374	0.2630
Xenobiotics	Sugar, sugar substitute, starch	sophorose	1.48	0.1616	0.1390
