# Supplementary material for: Survival Response to Increased Ceramide Involves Metabolic Adaptation through Novel Regulators of Glycolysis and Lipolysis
Source: PLoS Genet. 2013 Jun 20;9(6):e1003556. doi: 10.1371/journal.pgen.1003556 (PMC3688504; doi:10.1371/journal.pgen.1003556)
Supplement: Text S1 — Methodology detailed in Supplemental Materials and Methods. Additional details are provided for microarray analysis, metabolic assays including measurements of TAG, trehalose, ATP, free fatty acid and glycerol, QPCR analysis, immunofluorescence microscopy, activity measurements for glycolytic enzymes, assays for mitochondrial oxidative phosphorylation complexes and feeding of Pseudomonas entomophila. (DOCX) [file pgen.1003556.s013.docx]

**SUPPLEMENTAL MATERIALS AND METHODS**

**Ceramide and Ceramide 1-phosphate estimation**

Sphingolipid enriched fractions were prepared from 100 flies in each batch and d14 sphingoid base containing ceramides and ceramide 1-phophate were estimated by mass spectrometry (Xevo TQ-S, Waters) following previously published protocols [1,2].

**Microarray analysis**

For analysis, RMA method in Affymetrix package from Bioconductor was used in R to summarize the probe level data and normalize the dataset to remove across array variation [3]. Moderated t-test in Limma package was used to determine whether a gene’s expression level differs between mutant and control animals [4]. Genes with adjusted p-value using B-H method were considered for follow-up study [5]. Log transformed data were used in subsequent analysis and plotting.

**Metabolic assays**

**TAG measurement**

For TAG assays, 10 flies were homogenized in 100–150 μl PBST (PBS, 0.1% Tween 20) on ice, heated at 70°C for 5 minutes to inactivate endogenous enzymes, and the homogenate was cleared by centrifugation for 5 min at 12,000 rpm. The triglycerides in the supernatant were estimated using a serum triglyceride determination kit (TR0100; Sigma, St. Louis, MO) following the protocol from manufacturer and measured spectrophotometrically at 540 nm. Each sample was normalized for total protein as determined by modified Lowry method.

**Trehalose measurement**

Trehalose was measured from 8-10 flies after a 5-h of starvation on water. Whole flies were homogenized in 100–150 μl PBST (PBS, 0.1% Tween 20) on ice, heated at 70°C for 5 minutes to inactivate endogenous enzymes, and the homogenate was cleared by centrifugation for 5 min at 12,000 rpm, and 10 μl of homogenate was used to first measure glucose with glucose reagent (GAGO20, Sigma). Total glucose was measured by reading absorbance at 340 nm after 10 min of incubation at room temperature. To measure trehalose in the samples, porcine kidney trehalase (Sigma, T8778) was added (final concentration of 0.05 units.ml^-1^), and the solution returned to 37°C overnight, followed by a second reading of absorbance at 340 nm. Trehalose levels were obtained by subtracting the amount of free glucose in the untreated sample from the total glucose present in the sample treated with trehalase. Each sample was normalized to total protein.

**ATP measurement**

A bioluminescence assay based on luciferase-catalyzed oxidation of luciferin was used to determine the amount of ATP present in mitochondria using an ATP assay kit following the manufacturer’s instruction (Calbiochem, EMD Biosciences). Bioluminescence was measured using a luminometer (BioTek Synergy HT). Samples were normalized to total protein. A standard curve using known ATP concentrations was plotted to allow calculation of nmoles of ATP / mg protein.

**Measurement of free fatty acid and glycerol**

Assessment of fatty acid is through a coupled reaction involving acyl-CoA synthetase and acyl-CoA oxidase by measuring optical density at 540-550nm (Zenbio, NC). Detection of free glycerol involves phosphorylation to glycerol phosphate, which is oxidized by glycerol phosphate oxidase. The hydrogen peroxide formed is converted to a quinoneimine dye and increase in absorbance at 540nm is measured (Zenbio, NC). Standard curves using known concentrations of free fatty acid and glycerol were plotted to allow calculation of fatty acid and glycerol /mg protein.

**QPCR analysis**

Total RNA was extracted from at least 20 adult flies, 40 guts or mouse tissues of each genotype using TRIzol reagent (Invitrogen) following manufacturer protocols. Sample concentrations were measured using a Nanodrop spectrophotometer and cDNA was prepared from 0.5 or 1 μg of RNA in a 20 μl reaction using the SuperScript III First–Strand Synthesis kit (invitrogen) following the supplier´s protocol. For real time PCR, the reaction consisted of cDNA template (aliquot of cDNA first strand reaction), forward and reverse primers (200 nM final concentration) and SYBR Green Supermix (Invitrogen) in a total volume of 20μl. Real time PCR was performed in the ABI PRISM 7000 Sequence Detection System (Applied Biosystems) using SYBR Green Supermix (Invitrogen). Reactions were normalized to RP49 levels. All assays were done in duplicate and three independent experiments were performed. Primers used for real-time PCR were as follows:

| Hex A | F- ATATCGGGCATGTATATGGG | R- CAATTTCGCTCACATACTTGG |
| --- | --- | --- |
| Hex C | F-GGCTATACTCAACGATACCAC | R- CGCAATAGGTCCACATTCTC |
| Pgi | F-ACTGTCAATCTGTCTGTCCA | R- GATAACAGGAGCATTCTTCTCG |
| Pfk | F-AGCTCACATTTCCAAACATCG | R- TTTGATCACCAGAATCACTGC |
| Ald | F-GGCAAGAAGGAGAACATTGC | R- CAACCAAACGCCTTAGTAGG |
| Tpi | F-AGATCAAGGACTGGAAGAACG | R- ACCTCCTTGGAGATGTTGTC |
| Gapdh | F-CTCGCATATAATCACGCGTC | R- CACCTTGCCATACTTCTTGTC |
| Pgk | F-CTGATTGAGAACCTTCTGGAC | R- CTTCTCCACCAGTTTCTCGA |
| Pglym | F-AGAACATCGTCAAGGATCCC | R- GGTTGTCTAAATGCTTGACGA |
| Eno | F-CAACATCCAGTCCAACAAGG | R- GTTCTTGAAGTCCAGATCGT |
| Pyk | F-GTGCCACTCATCTACAAGGA | R- ATGAAGCCGTTCTTCTTTCC |
| dCERK | F-CGGGCAGAAGTGGTGATATT | R- CCTGCCAAGCTGTAGTCCTC |
| BMM | F-TCCCTCCTTCAACATCCAG | R- TGTGCAGTCGTCCATTCAC |
| CG11055 | F-GATCCATTCCTGTCGCCGTA | R- GTTTGAAGCAACGTCTGCAGAGA |
| CG8552 | F-GCGGCACCCTGAATTCAATG | R- GAGTCTTGGTACGTTCCTTGA |
| CG6277 | F-GAACAGTGGATGGAAGCCCA | R- GAGGCGGTGATCTTCTTGC |
| CG8093 | F-GCACCGCATACCGTACTC | R- TCTGGCATTACCCATCCA |
| dFOXO | F-GACAATGCCTGGAGGTGCTCAAT | R-TGAGGTTGCTAATGTTGCTGATGC |
| RP49 | F-AAGCTGTCGCACAAATGGCG | R- GCACGTTGTGCACCAGGAAC |
| Mouse FOXO1a | F-CTACGAGTGGATGGTGAAGAGC | R-CCAGTTCCTTCATTCTGCACTCG |
| Mouse FOXO3a | F-CCTACTTCAAGGATAAGGGCGAC | R-GCCTTCATTCTGAACGCGCATG |
| Mouse FOXO4 | F-TCTACGAATGGATGGTCCGCAC | R-CTTGCTGTGCAAGGACAGGTTG |
| Mouse Hexokinase | F-GGGCATGAAGGGCGTGTCCC | R-TCTTCACCCTCGCAGCCGGA |
| Mouse phosphoglycerate  mutase | F-AAGCACGGGGAGGAGCAGG | R-TGCATAGCGGCGGTCCTTGC |
| Mouse pyruvate kinase | F-TCGCATGCAGCACCTGATT | R-CCTCGAATAGCTGCAAGTGGTA |
| Mouse actin | F-GGACTCCTATGTGGGTGACG | R-CTTCTCCATGTCGTCCCAGT |

**Immunofluorescent staining and microscopy**

For gut dissection, the entire gastrointestinal tract was pulled from the posterior end directly into fixation medium containing 1X PBS and 4% formaldehyde. Guts were fixed in this medium for 3 hours. Subsequent washes and incubations with primary and secondary antibodies were done in a solution containing 1X PBS, 0.5% BSA, 0.1% TritonX-100. Primary antibody used was mouse anti-Armadillo (N2 7A1,1:20) obtained from the Developmental Studies Hybridoma Bank (developed under the auspices of the NICHD and maintained by The University of Iowa, Department of Biology, Iowa City, IA). 25-30 guts were stained per treatment. Secondary antibody used was goat anti-mouse IgG conjugated to Alexa 568 (1:2000, Molecular Probes, Eugene, OR). DAPI (Vectorshield, Vector Lab, Burlingame, CA) was used at 1:1 dilution in PBS. Images were taken using a Nikon Spinning Disk confocal microscope.

**Activity measurements of glycolytic enzymes**

Phosphoglycerate mutase: For measuring Pglym activity, approximately 1000 flies per batch were ground in buffer (50 mM Tris, pH 7.4, 2 mM EDTA), using a mortar and pestle. The mixture was spun at 10,000RPM for 15 min at 4°C. The supernatant was recovered by filtration through Acrodisc syringe filter (0.45 μm pore size). The assay for Pglym was modified from a published protocol [6]. This assay measures formation of phosphoenolpyruvic acid in the presence of excess enolase. 50 μg of supernatant was added to 3 μl of 50 mM 3-phosphoglyceric acid trisodium salt, 10 μmol of MgSO_4_, and 10 units of enolase in a 1 cm light path quartz cell. The rate of increase in absorbance at 240 nm was measured using a Beckman Coulter DU 640 spectrophotometer, and specific activity of the enzyme was determined utilizing a molar extinction coefficient of 1.75 mM ^-1^cm^-1^.

Pyruvate kinase: For measuring Pyk activity, flies were washed with buffer (50 mM Tris pH 7.5,1 mM-EDTA, 2 mM-β-mercaptoethanol, 50 mM-KCl), and homogenized in the same buffer. The homogenate was clarified by a 15 min centrifugation at 12,000g, and then the supernatant was centrifuged 150,000g for 1 h at 4°C. Pyk activity was assayed spectrophotometrically by measuring the decrease in absorbance at 340nm [7]. The assay solution contained 30 mM Hepes buffer, pH 6.8, 5 mM MgCl_2_, 0.15 mM NADH, 2 mM ADP, 9 units of lactic dehydrogenase and 50 μg of extract. After measuring the endogenous rate of NADH oxidation, the reaction was initiated by the addition of 1 mM PEP. The specific activity was calculated using an extinction coefficient of 6.22 mM ^-1^cm^-1^.

Enolase: For estimating Eno activity, flies were crushed in buffer (15 mM sodium-phosphate pH 7.2, containing 4 mM MgSO_4_), using a mortar and pestle. The extract was clarified at 12,000g and the supernatant was recovered after ultracentrifugation at 100,000g for 1 h at 4°C. Eno activity was measured in reaction buffer containing 50 mM imidazole-HCl, pH 6.8, 2 mM MgSO_4_, 40 mM KCl, and 100 μg of high speed supernatant [8]. The assay was started by the addition of 1 mM-2-phosphoglycerate and monitored at 240nm. The specific activity was determined by using an extinction coefficient 1.25 mM^-1^cm^-1^.

Hexokinase: To measure Hex activity, flies were homogenized in extraction buffer containing 10 mM Tris pH 7.4, 250 mM sucrose, 1 mM dithiothreitol, 1 mM EDTA, 10 mM glucose, and 1 mM diisopropylfluorophosphate. The extract was centrifuged at 800g for 10 min at 4°C and the supernatant was again centrifuged at 48,000g for 20 min at 4°C. The activity was assayed based upon the reduction of NAD through a coupled reaction with glucose-6-phosphate dehydrogenase and was determined spectrophotometrically by measuring the increase in absorbance at 340 nm [9]. The assay mixture contained 50 mM Tris-HCl, pH 8.0, 13.3 mM MgCl_2_, 110 mM glucose, 0.55 mM ATP, 0.22 mM NAD and 1 unit of glucose-6-phosphate dehydrogenase. The reaction was initiated by adding 50 μg of extract. The specific activity was calculated using an extinction coefficient 6.22 mM^-1^cm^-1^.

Lactate dehydrogenase: To measure Ldh activity, crude extracts were prepared by homogenizing flies in 2 ml of 50 mM potassium phosphate buffer pH 7.4 and 0.1mM DTT. The homogenate was centrifuged at 100,000g for 30 min and passed through a Sephadex G–25 column at 4°C. The determination of Ldh activity with pyruvate as a substrate was conducted using a modification of the published method [10]. The composition of the standard reaction system was: 50 mM potassium phosphate buffer, pH 7.4; 0.5 mM NADH; 0.3 mM sodium pyruvate; and extract (usually 0.1 ml of a 1: 3 dilution of the original preparation), in a final volume of 3 ml. The activity was associated with a decrease in absorbance at 340 nm, which was measured spectrophotometrically during a 3 min period. Specific activity was calculated by using an extinction coefficient 6.22 mM^-1^cm^-1^.

**Assays for mitochondrial oxidative phosphorylation complexes**

Mitochondria were isolated from approximately 1000 flies per batch. Flies were homogenized in mitochondrial isolation buffer; 250 mM sucrose, 10 mM Tris pH 7.4, 0.15 mM MgCl_2_ using a mortar and pestle. The homogenate was centrifuged at 4,000 X g for 15 min at 4°C and passed through an acrodisc syringe filter (0.45 μm pore size). The supernatant was spun twice at 16,000 X g for 30 min. The mitochondria- enriched pellet, was washed and resuspended in isolation buffer. Mitochondrial enrichment was monitored by blotting for Porin, a mitochondrial marker.

**Complex I: NADH ubiquinone oxidoreductase**

Oxidation of NADH at 340 nm was followed using Beckman DU 640 spectrophotometer at room temperature. The reaction buffer contained 250 mM sucrose, 1 mM EDTA, 50 mM Tris-HCl pH 7.4, 2 mM KCN to which Decylubiquinone (10 µM) and 50 µg of the mitochondrial protein were added. The reaction was initiated by adding 50 µM NADH and monitored through the linear absorbance decrease for 3 min. Rotenone (10 µg) was then added and decline in absorbance was monitored for additional 3 min. The rotenone sensitive complex I activity was calculated by using an extinction coefficient of

6.22 mM^-1^cm^-1^ [11].

**Complex II: Succinate dehydrogenase**

Complex II activity was measured by following the decrease in absorbance at 600 nm because of the reduction of 2, 6-dichlorophenol indophenol. Mitochondrial protein (30 µg) was incubated for 10 min in a reaction mixture of 50 mM potassium phosphate, pH 7.4, 20 mM succinate and 2 mM KCN. This was followed by addition of antimycin A (2 µg/mL), rotenone (2 µg/mL) and 10µM decylubiquinone and blank recorded. The reaction was initiated by adding 50 µM DCPIP and change in absorbance monitored for 3 min. Complex II activity was calculated by using an extinction coefficient of 19.1 mM^-1^cm^-1^ [12].

**Complex III: Ubiquinol cytochrome c reductase**

Complex III activity was measured by following the increase in absorbance at 550 nm because of the reduction of cytochrome c. The reaction mixture containing 250 mM sucrose, 1 mM EDTA, 50 mM Tris-HCl, pH 7.4, 2 mM KCN, 50 µM cytochrome c, was incubated with 30 µg mitochondrial protein for 10 min. The reaction was initiated by adding 50 µM DBH2, and the increase in absorbance at 550 nm was monitored for 3 min. The experiment was repeated with 5 µg/mL antimycin A added to the incubation mixture to determine the antimycin-insensitive activity. Complex III activity was calculated by using extinction coefficient 19.0 mM^-1^cm^-1^ [13].

**Complex IV: Cytochrome c oxidase**

Cytochrome c oxidase activity was measured by following the oxidation of reduced cytochrome c at 550 nm. The reaction was initiated by adding 20 µM reduced cytochrome c to 20 µg of mitochondrial protein in10 mM potassium phosphate buffer pH 7.4, and decrease in absorbance was recorded for 3 min. Complex IV activity was calculated using extinction coefficient 19.0 mM^-1^cm^-1^ [12].

**Complex V: ATP synthase**

The assay relies on linking the ATP synthase activity to NADH oxidation via the conversion of phosphoenolpyruvate to pyruvate by pyruvate kinase and then pyruvate to lactate by lactate dehydrogenase. The reaction buffer contained 250 mM sucrose, 50 mM KCl, 5mM MgCl_2_, 2 mM KCN, and 20 µM Tris-HCl, pH 7.5. Before the test, 0.25 mM NADH, 1 mM phosphoenol pyruvate, 2.5 units/ml lactate dehydrogenase, and 2 units/ml pyruvate kinase were added to reaction buffer. The reaction was started by adding 40 µg mitochondrial protein. The change in absorbance was recorded over 3 minutes at 340 nm. To determine the oligomycin-sensitive activity the experiment was repeated with 6 µg/mL oligomycin. Complex V activity was calculated by using extinction coefficient 6.22 mM^-1^cm^-1^ [12].

**Feeding of *Pseudomonas entomophila***

Oral ingestion of bacteria was carried out as previously described [14]. Survival of flies was monitored at 24h intervals.

**REFERENCES**

1. Dasgupta U, Bamba T, Chiantia S, Karim P, Abou –Tayoun AN, et al. (2009) Ceramide kinase regulates phospholipase C and phosphatidylinositol 4, 5, bisphosphate in phototransduction. Proc. Natl. Acad. Sci. U.S.A. 106: 20063-20068.

2. Yonamine I, Bamba T, Nirala NK, Jasmin N, Kosakowska-Cholody T, et al. (2011) Sphingosine kinases and their metabolites modulate endolysosomal trafficking in photoreceptors. J Cell Biol 192: 557-567.

3. Irizarry RA, Hobbs B, Collin F, Beazer-Barclay YD, Antonellis KJ, et al. (2003) Exploration, Normalization, and Summaries of High Density Oligonucleotide Array Probe Level Data. Biostatistics 4: 249-264.

4. Smyth GK (2004) Linear models and empirical bayes methods for assessing differential expression in microarray experiments. Stat Appl in Genet Mol Biol 3: Article 3.

5. Benjamini Y, Hochberg Y (1995) Controlling the false discovery rate: a practical and powerful approach to multiple testing. J R.Statist Soc B 57: 289-300.

6. Grisolia S, Carreras J (1975) Phosphoglycerate mutase from yeast, chicken breast muscle and kidney (2,3-PGA-dependent). Methods Enzymol 42: 435-450.

7. Malcovati M, Valentini G, Kornberg HL (1973) Two forms of pyruvate kinase in E.coli : their properties and regulation. Acta Vitaminol Enzymol 27: 96-111.

8.Baranowski T, Wolna E (1975) Enolase from human muscle. Methods Enzymol 42: 335-338.

9. Beutler E (1975) Red Cell Metabolism: A manual of biochemical methods. Ed.

2 :38-70, Grune and Stratton, New York.

10. Burke WF, Gracy R, Harris W (1972) Studies on enzymes from parasitic helminthes. 3. purification and properties of lactate dehydogenase from the tape worm, Hymenolepis diminuta*. Comp Biochem Physiol* B 43: 345-359.

11. Birch-Machin MA, Turnbull DM (2001) Assaying mitochondrial respiratory complex activity in mitochondria isolated from human cells and tissues. Methods Cell Biol 65: 97-117.

12.Thorburn DR, Chow CW, Kirby DM (2004) Respiratory chain enzyme analysis in muscle and liver. Mitochondrion 4: 363-375.

13. Luo C, Long J, Liu J ( 2008) An improvement spectrophotometric method for a more specific and accurate assay of mitochondrial complex III activity. Clin Chim Acta 395: 38-41.

14. Chatterjee M, Ip YT (2009) Pathogenic stimulation of intestinal stem cell response in *Drosophila*. J Cell Physio 220: 664-671.
